# Structure-Function Analysis of STRUBBELIG, an Arabidopsis Atypical Receptor-Like Kinase Involved in Tissue Morphogenesis

**DOI:** 10.1371/journal.pone.0019730

**Published:** 2011-05-16

**Authors:** Prasad Vaddepalli, Lynette Fulton, Martine Batoux, Ram Kishor Yadav, Kay Schneitz

**Affiliations:** Entwicklungsbiologie der Pflanzen, Technische Universität München, Freising, Germany; Instituto de Biología Molecular y Celular de Plantas, Spain

## Abstract

Tissue morphogenesis in plants requires the coordination of cellular behavior across clonally distinct histogenic layers. The underlying signaling mechanisms are presently being unraveled and are known to include the cell surface leucine-rich repeat receptor-like kinase STRUBBELIG in Arabidopsis. To understand better its mode of action an extensive structure-function analysis of *STRUBBELIG* was performed. The phenotypes of 20 EMS and T-DNA-induced *strubbelig* alleles were assessed and homology modeling was applied to rationalize their possible effects on STRUBBELIG protein structure. The analysis was complemented by phenotypic, cell biological, and pharmacological investigations of a *strubbelig* null allele carrying genomic rescue constructs encoding fusions between various mutated STRUBBELIG proteins and GFP. The results indicate that *STRUBBELIG* accepts quite some sequence variation, reveal the biological importance for the STRUBBELIG N-capping domain, and reinforce the notion that kinase activity is not essential for its function in vivo. Furthermore, individual protein domains of STRUBBELIG cannot be related to specific *STRUBBELIG*-dependent biological processes suggesting that process specificity is mediated by factors acting together with or downstream of *STRUBBELIG*. In addition, the evidence indicates that biogenesis of a functional STRUBBELIG receptor is subject to endoplasmic reticulum-mediated quality control, and that an MG132-sensitive process regulates its stability. Finally, *STRUBBELIG* and the receptor-like kinase gene *ERECTA* interact synergistically in the control of internode length. The data provide genetic and molecular insight into how *STRUBBELIG* regulates intercellular communication in tissue morphogenesis.

## Introduction

Tissue morphogenesis depends on extensive intercellular signaling. In plants the situation is complicated by the fact that plant cells are encased by cell walls and do not move relative to each other. Thus, alterations in cell size and shape need to be coordinated between cells of a tissue and orchestrated with cell wall dynamics. It is a salient topic of plant biology to unravel the mechanistic basis of the necessary communication.

Intercellular signaling processes in plants depend on two basic types of mechanisms: a combination of small ligands, capable of moving through the cell wall, and their receptors and intercellular movement of molecules passing through plasmodesmata [1]–[3]. Cell surface receptor-like kinases (RLKs) naturally belong to the former class and are involved in many short-range intercellular signaling processes. The Arabidopsis genome encodes more than 600 RLK genes [4]. This large number may relate to the salient role RLKs play in plant immunity [5]–[7]. Several RLKs are known to be important for the control of organ size and shape [8]–[10]. Well-characterized examples include the brassinosteroid hormone receptor BRASSINOSTEROID INSENSITIVE 1 (BRI1) [11], [12], the organ shape regulator ERECTA (ER) [13]–[16], the stem cell regulator CLAVATA1 (CLV1) [17], [18], and ARABIDOPSIS CRINKLY 4 (ACR4) which is involved in epidermal differentiation and formative cell division control in the root pericycle [19]–[22]. ACR4 is the Arabidopsis homolog of maize CRINKLY 4 (CR4) [23], [24]. Except for ACR4 and CR4 these RLKs carry leucine-rich repeats (LRRs) in their extracellular domains and thus encode members of the large LRR-RLK subfamily of RLKs. ACR4 and CR4 feature TNFR-like cysteine-rich repeats and fall into a different family of RLKs [4].

*STRUBBELIG* (*SUB*) is another LRR-RLK gene with a role in tissue morphogenesis of many plant organs [25]. Originally identified in a screen for ovule mutants [26] *SUB* was shown to be important not just for the initiation and outgrowth of ovule integuments but also for floral organ shape, stem height and shape, leaf shape and root hair patterning [25], [27], [28]. *SUB* is a member of the small *STRUBBELIG RECEPTOR FAMILY* (*SRF*)/*LRRV* gene family [4], [29]. Another member, *SRF4*, affects leaf size [29] while *SRF3* plays a role in plant pathogen response and potentially in speciation [30]. For other *SRF* genes, such as *SRF4* or *SRF7*, a role in cell wall biology was proposed [29].

At the cellular level an important function of *SUB* relates to the control of cell division planes. Integument initiation relies on oriented cell divisions. Furthermore, division planes of L1 and L2 cells are frequently misoriented in floral meristems of *sub* mutants. To some extent *SUB* is also involved in the regulation of cell proliferation, as reduced cell numbers are observed in integuments and stems of *sub* mutants [25], [27]. *SUB* signaling appears to be important for the coordination of such cellular behavior across histogenic cell layers. Although *SUB* is expressed in a broad fashion in floral meristems and young ovules [25], expression of a functional SUB:EGFP fusion protein to the L1 layer is sufficient to rescue the L2 division plane defects in floral meristems [31]. In addition, SUB:EGFP expression in the distal nucellus of ovule primordia can rescue to a large extent defects in the integuments, tissue that originates from the central chalaza. Thus, it was proposed that *SUB* acts in a non cell-autonomous fashion and mediates inter-cell-layer signaling during floral development [31]. In this respect *SUB* may relate to *BRI1* [32], [33]. The mechanism of *SUB* signaling is presently being investigated and three additional genes with a role in this process have been identified [27]. *QUIRKY* (*QKY*) is one of them and encodes a putative membrane-bound protein with multiple C2 domains, a domain architecture that is analogous to known membrane trafficking proteins, such as ferlins and synaptotagmins [34], [35]. Thus, it was speculated that *SUB* signaling includes some sort of Ca^2+^-dependent membrane trafficking, a notion that would conveniently explain the non cell autonomy of *SUB* as well [27], [36].

Interestingly, SUB does not seem to depend on phospho-transfer activity of its kinase domain in vivo as evidenced by the absence of detectable in vitro kinase activity and the wild-type phenotype of *sub-1* plants carrying correspondingly mutated *SUB* cDNAs under the control of the strong and broadly expressed cauliflower mosaic virus 35S promoter (*35S::cSUB_K525E_ sub-1* or *35S::cSUB_E539A_ sub-1*) [25]. Thus, SUB likely represents a so-called atypical or dead kinase. A number of atypical kinases have been described in animals and plants and although their mode of action is still being investigated it is likely to include regulated protein-protein interactions [37]–[39]. Apart from SUB, plant examples include the maize RLK MARK that interacts with the functional GCN-like kinase MIK resulting in a stimulation of MIK activity [40] and AtCRR2, a homolog of ACR4 [41]. It is noteworthy that for some biochemically active RLKs, such as ACR4 or FEI1, kinase activity may not be functionally relevant [20], [42], an observation that was explained by a model where absence of kinase activity was complemented by redundant activities in a protein receptor complex [20].

In this study we performed a structure-function analysis to gain at a better molecular understanding of how the atypical RLK SUB regulates its various downstream signaling processes. Using a combination of genetic, cell biological and pharmacological approaches we provide evidence that *SUB* principally accepts sequence variability but that the N-capping domain in the extracellular domain of the SUB protein is important for its biological activity. In addition, the data indicate that delivery of functional SUB receptor to the plasma membrane is monitored by endoplasmic reticulum-mediated quality control. Furthermore, tissue-specific or cell-specific *SUB*-dependent processes do not appear to be integrated into the SUB mechanism by the receptor itself, through functionally differentiated protein domains, but likely via other components acting together with or downstream of SUB. One such component is encoded by *ERECTA*, a gene that synergistically interacts with *SUB* in the regulation of shoot internode length.

## Results and Discussion

### SUB structure prediction by homology modeling

**Figure 1:**
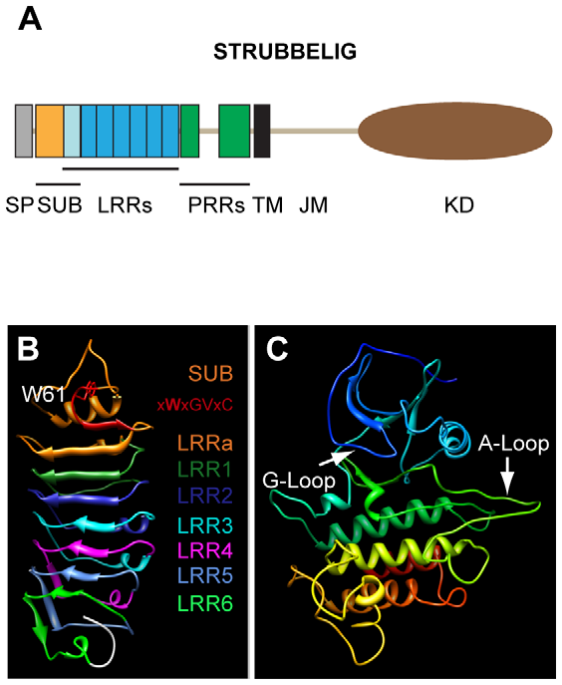
Predicted structure of the SUB protein. (A) Overview of the domain architecture of SUB. (B) Model of the extracellular domain encompassing the SUB-domain and the leucine-rich repeats. The SUB-domain is indicated by orange color. The imperfect CxWxGVxC motif with the conserved tryptophan is highlighted in red. Individual LRRs are marked by the respective colors. (C) Model of the kinase domain. Different colors arbitrarily denote distinct secondary structures to aid in visualization. The ATP-binding (G-loop) and substrate binding (A-loop) regions are marked. Abbreviations: JM, juxtamembrane domain; KD, kinase domain; LRR, leucine-rich repeat; PRR, proline-rich repeat; SP, signal peptide; SUB, SUB-domain; TM, transmembrane domain. Length of SUB protein: 768 amino acids.

The SUB protein was predicted to contain an extracellular domain (ECD) with a 24-aa signal peptide, an amino-terminal region of about 59 residues that is conserved between the LRRV/SRF members (termed SUB domain), six LRRs and a proline-rich region. The ECD is followed by a transmembrane domain (TM) and the intracellular juxtamembrane (JM) and the carboxy-terminally-located kinase domain (KD) [4], [25] (Figure 1) (an alignment of the Arabidopsis SRF protein sequences is given in Figure S1). It has a length of 768 amino acids and a calculated molecular weight of 84.5 kDa. Crystallographic information about the structure of SUB is presently lacking. To gain insights regarding the possible structure of SUB, which might help to rationalize the effect of some *sub* mutations (see below), we applied homology modeling using the Swiss-Model workspace [43]. The algorithms generated two models, one for the SUB/LRR region and one for the kinase domain (Figure 1B and 1C). The suggested template for the SUB/LRR region turned out to be polygalacturonase-inhibiting protein (PGIP2) from *Phaseolus vulgaris*, a leucine-rich repeat protein involved in plant defense [44]. The kinase domain was modeled after the tomato Pto kinase [45].

The model for the SUB/LRR region predicts that the SUB domain consists of a short amino-terminal α-helix, a structurally ill-defined region, a loop that is formed by a very highly conserved stretch of amino acids, a second loop carrying a strictly conserved tryptophan, a small β-strand, and finally a single imperfect LRR (termed LRRa) (Figure 1A, B; Figure S1). The structurally ill-defined region and the two loops fall into a region for which the prediction of the model may be less accurate than for the better supported LRRs (Figure S3). The SUB domain is then followed by another six LRRs (termed LRR1 to LRR6). Thus, it is likely that the ECD of SUB contains seven rather than the six LRRs originally identified. An imperfect CxWxGVxC motif, is located just before the LRRa region (Figure S1). This motif precedes the first LRR in many plant LRR-containing ECDs [46], [47]. The first half of the SUB domain thus likely represents an N-terminal capping domain thought to protect the hydrophobic core of the LRR in many plant extracellular LRR proteins [44], [47]–[50]. The model predicts that the LRRs form a curved structure with a slight right-handed twist, which carries eight β-strands located at its inner or concave side. In analogy to resolved structures of LRR proteins the β-strands are presumed to form an interface that can interact with other proteins [51] (sheet B1 in PGIP2 [44]). In addition, three additional β-strands form a second small β-sheet located at the bottom and to one side of the curved structure (Figure 1B). For PGIP2 it was proposed that this second β-sheet (sheet B2) may represent an additional protein-protein interaction site [44]. The model of the SUB kinase domain resembles a standard kinase structure with the smaller N-terminal and the bigger C-terminal lobes and shows no obvious structural peculiarities (Figure 1C, Figure S1) [52], [53].

### Identification and analysis of novel *sub* alleles

**Figure 2:**
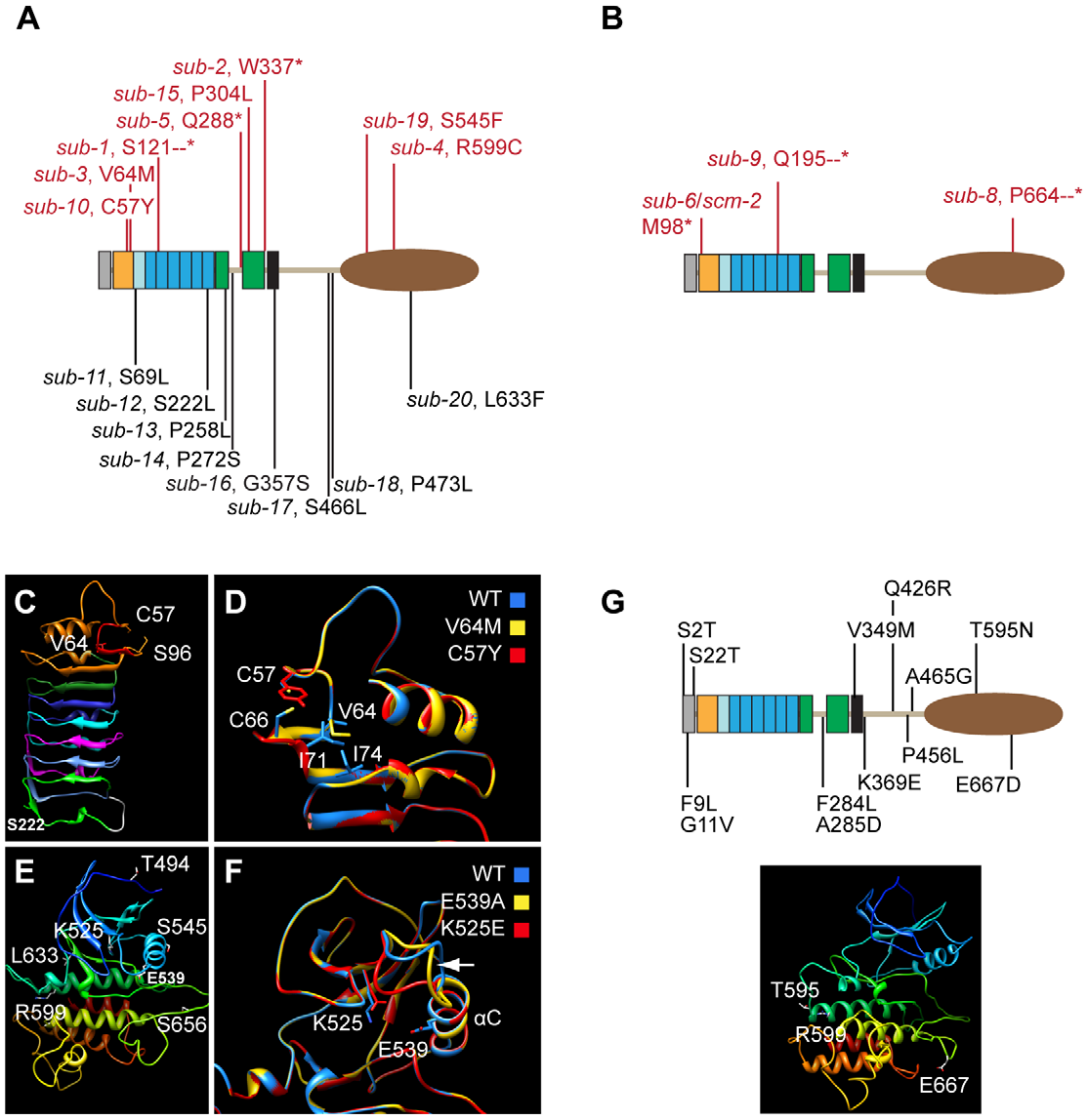
Molecular nature of mutations affecting SUB and homology models of mutant SUB variants. (A,B) Position of different *SUB* alleles. Phenotypic mutations are listed above the protein and depicted in red while aphenotypic mutations are listed below the protein. (A) EMS-induced point mutations. Stars denote premature stops. (B) T-DNA insertions. Dashes denote artificial amino acids (see also Table 1). Length of SUB protein: 768 amino acids. (C–F) Homology models of SUB variants. (C, D) SUB-domain plus leucine-rich repeats. Residues affected by mutation are highlighted. (C) Wild-type. The SUB-domain is indicated by the orange color. The imperfect CxWxGVxC motif is highlighted in red. Individual LRRs are marked by respective colors as in Figure 1B. (D) Overlay of wild-type and two mutant models. Focus resides on the region encompassing the SUB-domain and the first leucine-rich repeat. (E, F) Kinase domain. (E) Wild-type. Different colors arbitrarily denote distinct secondary structures to aid in visualization. (F) Overlay of wild-type and two mutant models. Focus is on the region encompassing the G-loop and the αC helix. The arrow marks the predicted structural variation in the loop that connects the β3 sheet with the αC helix. (G) Upper panel: position of different natural variation alleles. Lower panel: the T595 and E667 residues affected residues in the STRUBBELIG kinase domain are marked in the kinase domain homology model. The R599 residue mutated in *sub-4* is highlighted for orientation. Different colors arbitrarily denote distinct secondary structures to aid in visualization.

**Figure 3:**
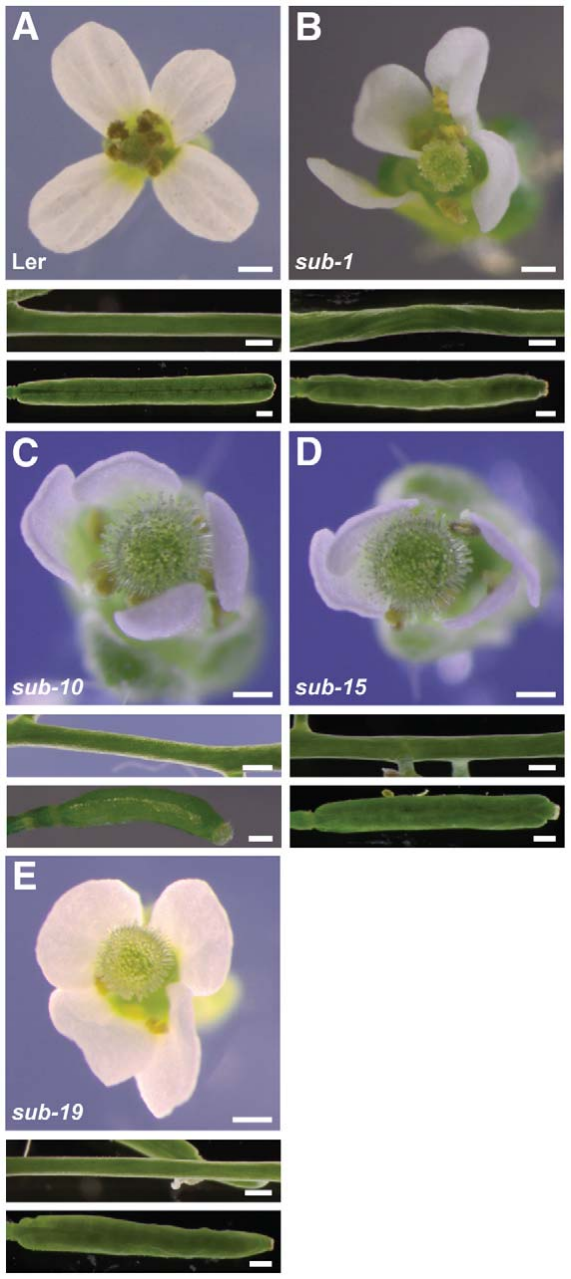
Comparative analysis of the *sub-10*, *sub-15* and *sub-19* phenotypes. Flower morphology and stem and silique shape. (A) Wild type (L*er*). (B) *sub-1*. (C) *sub-10*. Siliques are a bit shorter compared to *sub-1*. (D) *sub-15*. Stem twisting is not quite as strong as in *sub-1*. (E) *sub-19*. Resembles *sub-1*. Scale bars: 0.5 mm.

In previous work we identified five EMS-induced *sub* alleles (*sub-1* to *sub-5*) in different forward genetic screens using L*er* as a background [25], [26] (Figure 2A, Table 1, Figure S1). We also scanned public T-DNA collections and identified four insertions in *SUB* in either Col (*sub-6*, *sub-7*, *sub-9*) or Ws-2 (*sub-8*) background (Figure 2B). One line (*sub-7*) had a complex T-DNA integration pattern and was not analyzed further. To further elucidate structure-function relations of *SUB* additional EMS-induced alleles were identified in the Col *er-105* background using targeted-induced local lesions in genomes (TILLING) [54], in conjunction with the Seattle Arabidopsis TILLING facility (http://tilling.fhcrc.org/files/Welcome_to_ATP.html) [55]. A total of 26 lines with altered nucleotides in the *SUB* locus were identified. Of these 26 mutations 8 were located in introns and 7 were silent mutations (not shown). This left 11 mutations, named *sub-10* to *sub-20*, which resided in exons and were predicted to cause amino acid alterations in the SUB protein (Figure 2, Table 1, Figure S1). Interestingly, only three of these alleles resulted in a *sub* phenotype (*sub-10* (C57Y), *sub-15* (P304L), and *sub-19* (S545F)) (Figure 3). All EMS or T-DNA-induced *sub* alleles showing a mutant phenotype (phenotypic alleles) behaved recessively and segregated in a Mendelian fashion (not shown).

**Table 1:** Summary of *sub* alleles.

Allele	Mutagen	Mutation#	AA change	Background
*sub-1* §	EMS	G>A, 999	S121--*	L*er*
*sub-2* §	EMS	G>A, 2157	W337*	L*er*
*sub-3* §	EMS	G>A, 567	V64M	L*er*
*sub-4* §	EMS	C>T, 3127	R599C	L*er*
*sub-5* §	EMS	C>T, 2008	Q288*	L*er*
*sub-6*, SALK_086357	T-DNA	839/LB (intron 3)	M98*	Col
*sub-7*, GK-562F05-021689	T-DNA	complex insertion	N.D.	Col
*sub-8*, Wisconsin, T28P6.18	T-DNA	3478/LB	P664--*	Ws-2
*sub-9*, GARLIC_1158_D09	T-DNA	1548/LB	Q195--*	Col
*sub-10*	EMS	G>A, 547	C57Y	Col *er-105*
*sub-11* +	EMS	C>T, 583	S69L	Col *er-105*
*sub-12* +	EMS	C>T, 1728	S222L	Col *er-105*
*sub-13* +	EMS	C>T, 1916	P258L	Col *er-105*
*sub-14* +	EMS	C>T, 1960	P272S	Col *er-105*
*sub-15*	EMS	C>T, 2057	P304L	Col *er-105*
*sub-16* +	EMS	G>A, 2215	G357S	Col *er-105*
*sub-17* +	EMS	C>T, 2639	S466L	Col *er-105*
*sub-18* +	EMS	C>T, 2660	P473L	Col *er-105*
*sub-19*	EMS	C>T, 2966	S545F	Col *er-105*
*sub-20* +	EMS	C>T, 3302	L633F	Col *er-105*

§ previously described in Schneitz et.al. 1997 and/or Chevalier et.al. 2005.

+ aphenotypic mutations.

# the coordinates refer to the genomic sequence and relate to the ATG of *SUB* (At1g11130) of BAC T19D16 (see Chevalier et.al. 2005).

*premature stop.

–*premature stop preceded by artificial sequence of amino acids of variable length (*sub-1*: 2 aa; *sub-8*: 48 aa; *sub-9*: 8 aa).

Abbreviations: LB, Left border of T-DNA insertion; N.D., not determined.

In this section we discuss our results on the EMS-induced mutations. Our investigation of the T-DNA insertions is dealt with below. The *sub-1*, *sub-2*, and *sub-5* mutants are likely devoid of any *SUB* function as the mutations result in predictive shorter proteins that comprise part of the extracellular domain but lack the transmembrane and intracellular domains. Thus, the mutant proteins will not be able to transmit a signal across the plasma membrane [25] (Figure 2A). The phenotypes of several *sub* mutations have been described extensively. In short, *sub* mutants in the L*er* background show characteristic defects such as impaired integument development, twisted siliques, misshaped floral organs, and short and twisted stems (Figure 3) [25], [27].

We compared the above-ground morphology of all 16 EMS-induced *sub* alleles (Figure 2A, Table 1, Figure S1). Eight alleles exhibited a mutant phenotype with three predicted to be null alleles (the afore-mentioned *sub-1*, *sub-2*, *sub-5*) and five carrying amino acid substitutions (*sub-3* (V64M), *sub-4* (R599C), *sub-10* (C57Y), *sub-15* (P304L), *sub-19* (S545F)). The other eight alleles, all carrying amino acid substitutions, were aphenotypic. Morphological characteristics of the null allele *sub-1* and *sub-3* or *sub-4* (all in L*er*) were essentially identical suggesting that the latter two mutations result in amino acid changes that cause complete loss of SUB function [25]. Further, the three phenotypic TILLING alleles *sub-10*, *sub-15*, and *sub-19* (in Col *er-105*) also demonstrated *sub-1*-like phenotypes, although the alterations in floral morphology and stem shape of *sub-10* and *sub-15* were slightly less marked (Figure 3). Potentially, these two alleles could be hypomorphic or the somewhat milder phenotypes may relate to the presence of modifiers in the Col *er-105* background (see below). Overall, the analysis of the available EMS-induced mutations indicated that irrespective of their nature the phenotypic mutations all result in the loss of *SUB* function. *SUB* exhibits a different genetic behavior when compared to the *CLAVATA1* (*CLV1*) RLK gene. Interestingly, *clv1* null alleles show a weak phenotype whereas many *clv1* missense mutations lead to a strong phenotype [56]. It was reasoned that missense *clv1* alleles interfere with redundantly acting receptors, such as CLV2/CORYNE (CRN) and BAM1/2 [57]–[62]. A similar scenario does not seem to be the case for *SUB*. In addition, the results preclude the mapping of particular SUB domains to individual biological processes, such as stem or integument development. This suggests that organ or cell-specific aspects of *SUB* signaling may not be integrated at the level of the SUB receptor itself but involve other components that act together with or downstream of SUB. This notion is substantiated by additional genetic evidence involving *ERECTA* (see below).

Surprisingly, there was no strict correlation between degree of conservation of the altered residue throughout the Arabidopsis SRF family and presence of the *sub* phenotype. The five phenotypic amino acid substitutions affected either strictly conserved (*sub-4*, R599C; *sub-10*, C57Y), structurally conserved (*sub-3*, V64M), semi-conserved (*sub-19*, S545F), or nonconserved amino acids (*sub-15*, P304L). Furthermore, while many of the aphenotypic changes affect nonconserved residues (*sub-12* - *sub-14*, *sub-16* - *sub-18*), two aphenotypic mutations, *sub-11* (S69L) and *sub-20* (L633F), result in changes at amino acid positions that are strictly conserved [4], [29] (Figure S1). This finding indicates that *SUB* is able to accommodate a perhaps astonishing level of sequence variability even at conserved positions. Alternatively, aphentoypic alleles may affect a *SUB* function not revealed by our morphological analysis.

To investigate this issue further, we complemented our analysis on the effects of artificially induced *sub* mutations by an assessment of natural variation at the level of the SUB protein. We took advantage of the publically accessible 1001 genomes project (www.1001genomes.org) and analyzed the MPICao2010 dataset of full genome sequences produced by the Weigel laboratory at the Max Plank Institute for Developmental Biology. This dataset contains information from 80 wild-type Arabidopsis accessions. Twenty-four accessions were omitted from analysis due to sequencing-related uncertainties in the *SUB* sequence. The remaining 57 different SUB protein sequences, including the TAIR10 reference sequence for SUB, (At1g11130.1_REF) were investigated further revealing 13 distinct amino acid polymorphisms (Table 2, Figure S2). Eight accessions carried one polymorphism while six accessions carried either two or three alterations. There was no overlap between our set of artificially induced mutations and the naturally occurring alleles (Figure 2G). For the most part natural variation was observed at semi- or nonconserved residues (Figure S1). One notable exception was the T595N polymorphism in the kinase domain which occurred in several accessions (Table 2) and resided at a position that with the exception of the closely related SRF6 and SRF7, is usually occupied by either a threonine or a serine (Figure S1). Within the kinase homology model, T595 is predicted to reside at the end of a loop, yet precedes the crucial alpha helix in subdomain VIa (Figure 2G, see also below). We speculate that the type and position of the alteration may not noticeably interfere with the kinase domain structure. Alternatively, this polymorphism, as with some of the other naturally occurring polymorphisms, may be balanced by second-site mutations. For example, an altered site may have a biologically relevant negative effect on SUB conformation. However, it is conceivable that a second-site mutation in for example, a direct interactor of SUB may result in a protein that can still interact with the altered SUB protein and thus compensate for the principally deleterious effect. It is presently unclear if, and how often, this possibility actually occurs in the case of SUB in wild-type accessions. A different scenario, where accumulation of genetic incompatibilities between accessions can lead to reproductive isolation, has been described for SRF3 [30].

**Table 2:** Summary of SUB amino acid polymorphisms in different Arabidopsis accessions.

Position+	Change	Accession
2	S2T	ICE102, Qui-0, ICE61
9	F9L	ICE102, Qui-0
11	G11V	ICE102, Qui-0, ICE61
22	S22T	ICE102, Qui-0, ICE61
284	F284L	Pra-6
285	A285D	TueWa1-2, Vash-1
349	V349M	ICE120
369	K369E	Koch-1
426	Q426R	ICE61
456	P456L	ICE72
465	A465G	ICE120, Tuescha-9
595	T595N	Del-10, ICE107, Nie1-2, Ped-0, Koch-1
667	E667D	ICE72

+ Numbering starts at the N-terminal methionine of SUB.

Leaving such complications aside our assessment of natural variation in the SUB protein supports the sequence variability idea put forward earlier. Combining the artificially-induced mutations and natural variation polymorphisms we have now identified a total of 21 mutations scattered throughout the SUB protein, but noticeably absent from the LRR region, that seem to be of no obvious biological consequence for SUB activity. This corroborates the notion that SUB accepts a noticeable level of sequence variation. Interestingly, the extra-cellular LRR domain is largely untouched by aphenotypic mutations. The LRR domain is likely involved in the binding of the SUB ligand and thus seems an essential aspect of SUB activity, placing constraints on sequence variability.

### Homology modeling of mutant SUB/LRR and kinase domains

The biochemical and/or structural functions of the altered residues in the non-synonymous phenotypic *sub* alleles are presently unknown. We therefore took the SUB/LRR domain model and made predictions as to how different *sub* mutations may affect this domain. Our genetic analysis suggests that the conformation of the N-terminal capping domain appears to be critical for SUB activity as two phenotypic mutations, *sub-3* (V64M) and *sub-10* (C57Y) affect this domain. In the homology model V64 resides towards the top and at the beginning of the first small β-strand that is part of LRRa and that likely contributes to the potential ligand-binding interface (Figure 2C, D). However, the residue's side-chain points away from this interface, suggesting that V64 does not directly contribute to protein-protein interaction through this surface. In *sub-3* (V64M) the long side chain of the methionine may interfere with formation of a hydrophobic region that is generated by isoleucines 71 and 74 (Figure 2D), and affect the relative orientation of the first small β-strand and adjacent large β-strand of LRRa, and thus the architecture of LRRa per se. The *sub-10* (C57Y) mutation affects the first cysteine in the imperfect CxWxGVxC motif. The wild-type SUB model suggests that the strictly conserved C57 may contribute to folding or stabilization of the N-terminal capping domain via an intramolecular disulphide bond formation with C66 (Figure 2D). BRI1, for example, seems to carry such a disulphide bond at a related position [63] and a similar disulphide bond is critical for Cf-9 activity [47]. In *sub-10* this disulphide bond would not occur. In accordance with this notion data indicate C66 is also essential for SUB activity (see below). Alternatively, C57 may have a steric role independent of C66, which would be abolished in *sub-10*. Formally, as C57 and C66 are located at a solvent-exposed surface (Figure 2D), we also cannot rule out the possibility that C57 and C66 are required for intermolecular disulphide bridges. Given, however, their close proximity in the model and the importance of such cysteine pairs for the stabilization of LRR domains [50], we currently favor a role in the structure of the N-capping domain.

Two aphenotypic mutations also reside within the SUB/LRR domain, *sub-11* (S69L) and *sub-12* (S222L). The *sub-11* allele affects a strictly conserved serine at position 69 that localizes close to the two nearby cysteines C57 and C66. The model, however, predicts that S69 is located in the loop between the first and the second β-sheets of LRRa with the side-chain facing outwards and to the side of the protein (Figure 2C). This architecture and the nature of the side-chain exchange may explain the lack of a mutant phenotype in *sub-11*. The *sub-12* allele is characterized by a serine to leucine substitution at a non-conserved position (Figure 2C). The model predicts that S222 is located towards the end of the third small β-strand in sheet B2 with the side-chain pointing sideways and away from the protein. Thus, either the S222L substitution does not interfere with possible protein-protein interactions of sheet B2 or this β-sheet is not an interface for protein interaction in SUB and similar considerations as outlined for *sub-11* may apply.

The phenotypic *sub-4* and *sub-19* alleles hint at the importance of the kinase domain for SUB function. In *sub-4* a cysteine replaces the arginine at position 599. This residue is strictly conserved among the SRF members and is affected in, for example, the *bri1-8* and *bri1-108* alleles of BRI1 [64], [65]. The mutation resides in subdomain VIa. Conservation of an arginine at the equivalent position across many plant kinases implies an important function for this residue [25]. The model of the SUB kinase domain suggests that R599 is situated at end of the long alpha helix of subdomain VIa that runs through the back of the C-terminal lobe (Figure 2E). R599 may thus have a structural role. However, a KD model of SUB_R599C_ did not reveal obvious structural changes (not shown) and the exact role of this residue remains to be elucidated. The *sub-19* (S545F) mutation resides within the conserved αC helix, a mediator of conformational changes in the catalytic center [52], principally explaining its loss of function. Interestingly, however, another mutation in the αC helix (E539A) did not affect SUB activity (see below). Furthermore, *sub-20* (L633F), also situated in the kinase domain, was aphenotypic, indicating that the KD of SUB tolerates some sequence variability. The reason for this property of SUB awaits further investigation, as KD models of SUB_S545F_ and SUB_L633F_ were uninformative (not shown).

### Kinase activity is not essential for SUB function

**Figure 4:**
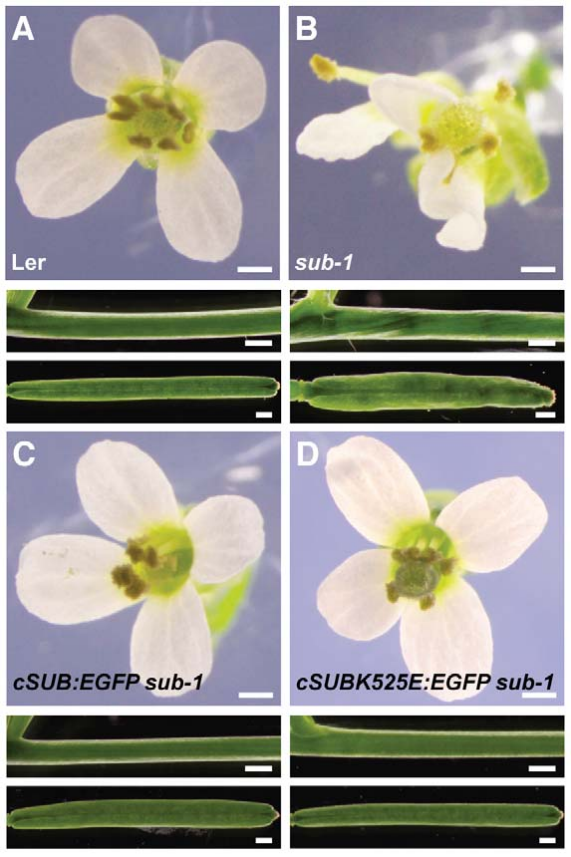
Genetic evidence that kinase activity is not essential for SUB function in vivo. Flower morphology and stem and silique shape of *sub-1* plants carrying *SUB::cSUB:EGFP* and *SUB::cSUB_K525E_:EGFP* reporter constructs. (A) Wild type (L*er*). (B) *sub-1*. (C) *SUB::cSUB:EGFP sub-1*. A functional construct whereby the *sub-1* phenotype is rescued. (D) *SUB::cSUB_K525E_:EGFP sub-1*. A functional construct. Plant appears wild type. Scale bars: 0.5 mm.

SUB is likely an atypical or dead kinase as several substitutions in the small lobe known to eliminate kinase activity, such as the well-known K525E substitution or the E539A alteration [66], [67], are tolerated in vivo. This was demonstrated by the rescued wild-type phenotype of *sub-1* plants expressing *35S::cSUB_K525E_* or *35S::cSUB_E539A_* transgenes [25]. The K525 resides in a β-strand (normally classified as β3) preceding a loop connecting β3 with the conserved αC helix, a mediator of conformational changes in the catalytic center [52], while residue E539 is part of the αC helix (Figure 2F). Interestingly, homology modeling predicts that both mutations result in different conformations for the loop that connects β3 and the αC helix (Figure 2F). However, our previous genetic results indicate that these conformational changes either do not occur or are irrelevant in vivo. To exclude that the use of the 35S promoter weights these results we generated *sub-1* plants that carried constructs in which the mutated *SUB* cDNA-based constructs were under control of endogenous *SUB* genomic fragments. Previous work established that reporter constructs that include genomic sequences 3.5 kb upstream, and 0.4 kb downstream, of *SUB*, recapitulate the spatial pattern of the *SUB* transcription [31]. Similar results were obtained with slightly larger upstream and downstream genomic sequences [28]. In addition, a *SUB* cDNA-based reporter construct under the control of the endogenous promoter, encoding a translational fusion between SUB and an enhanced version of GFP (SUB:EGFP) (*SUB::cSUB:EGFP*), was able to rescue all above-ground aspects of the *sub-1* mutant phenotype [31]. Using in vitro mutagenesis we introduced K525E and E539A mutations into the reporter (*SUB::cSUBmut:EGFP*). The *SUB::cSUB_K525E_:EGFP sub-1* and the *SUB::cSUB_E539A_:EGFP sub-1* plants exhibited a wild-type phenotype as well (Figure 4). These findings demonstrate that the previous use of the 35S promoter did not cause noteworthy artifacts and reinforces the notion that SUB is an atypical or dead kinase.

Although the SUB kinase domain is not essential for its function in vivo it is possible that phosphorylation of SUB by as yet unknown kinases is important. To test this possibility we altered two semi-conserved threonines (T486A/E and T494A) in the juxtamembrane and kinase domains, respectively. In addition, we changed the single serine in the activation loop (S656A) (Figure S1). Correspondingly, all three *35S::cSUBmut* constructs rescued the *sub-1* phenotype (not shown). This finding indicates that phosphorylation of these residues is not required for SUB function.

### Nonfunctional *SUB::cSUBmut:EGFP* reporters fail to express detectable signals

**Figure 5:**
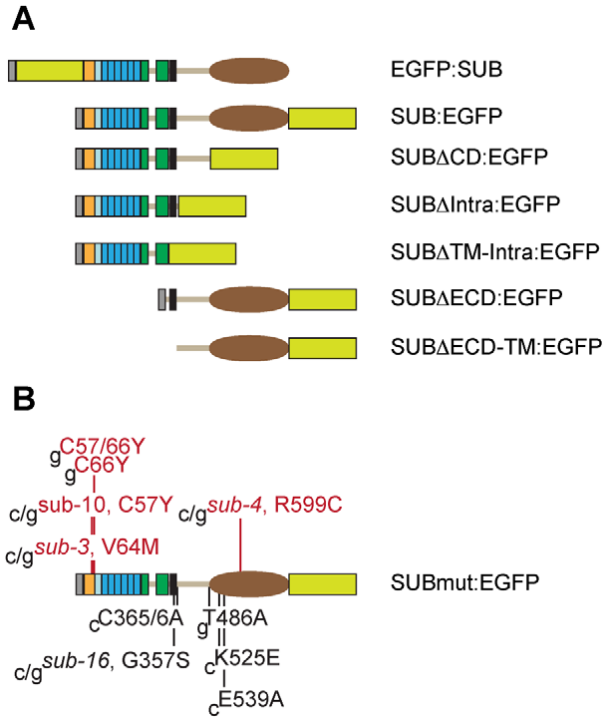
Synopsis of SUB:EGFP variants generated by in vitro mutagenesis. The domain architecture of SUB is depicted as in Figure 1. All constructs were tested for rescue of the *sub-1* phenotype. The EGFP tag is indicated by a yellow/green box. (A) N- and C-terminal fusions of EGFP to wild-type SUB and C-terminal fusions of EGFP to various SUB deletions. All constructs included endogenous *SUB* promoter elements and the *SUB* cDNA. All the deletions were unable to rescue the *sub-1* phenotype. (B) Point mutations. Mutations resulting in a failure to rescue the *sub-1* phenotype are listed above the protein and depicted in red while mutations that still rescued the *sub-1* phenotype are listed below the protein and shown in black. Mutant constructs were driven by endogenous *SUB* promoter elements and included *SUB* coding sequences derived from cDNA (c) or genomic DNA (g, including all *SUB* introns). Length of SUB protein: 768 amino acids. Abbreviations: CD, kinase domain; ECD, extra-cellular domain; Intra, intracellular domain; TM, transmembrane domain.

Analysis of different *SUB::cSUBmut:EGFP sub-1* plants and the rationalization by homology modeling of the effects of individual mutations on a protein also depend on the correct cellular and subcellular localization of the mutated protein. To address this issue we generated by in vitro mutagenesis a set of reporters encoding mutant SUB:EGFP fusion proteins that carried either deletions or individual point mutations (Figure 5). We tested the capability of the individual mutant constructs to restore *SUB* function in *sub-1* plants by analyzing the phenotype of *SUB::cSUBmut:EGFP sub-1* plants. Simultaneously, we also assayed the EGFP signal in these plants to assess the cellular and subcellular distribution of the mutant fusion protein.

**Figure 6:**
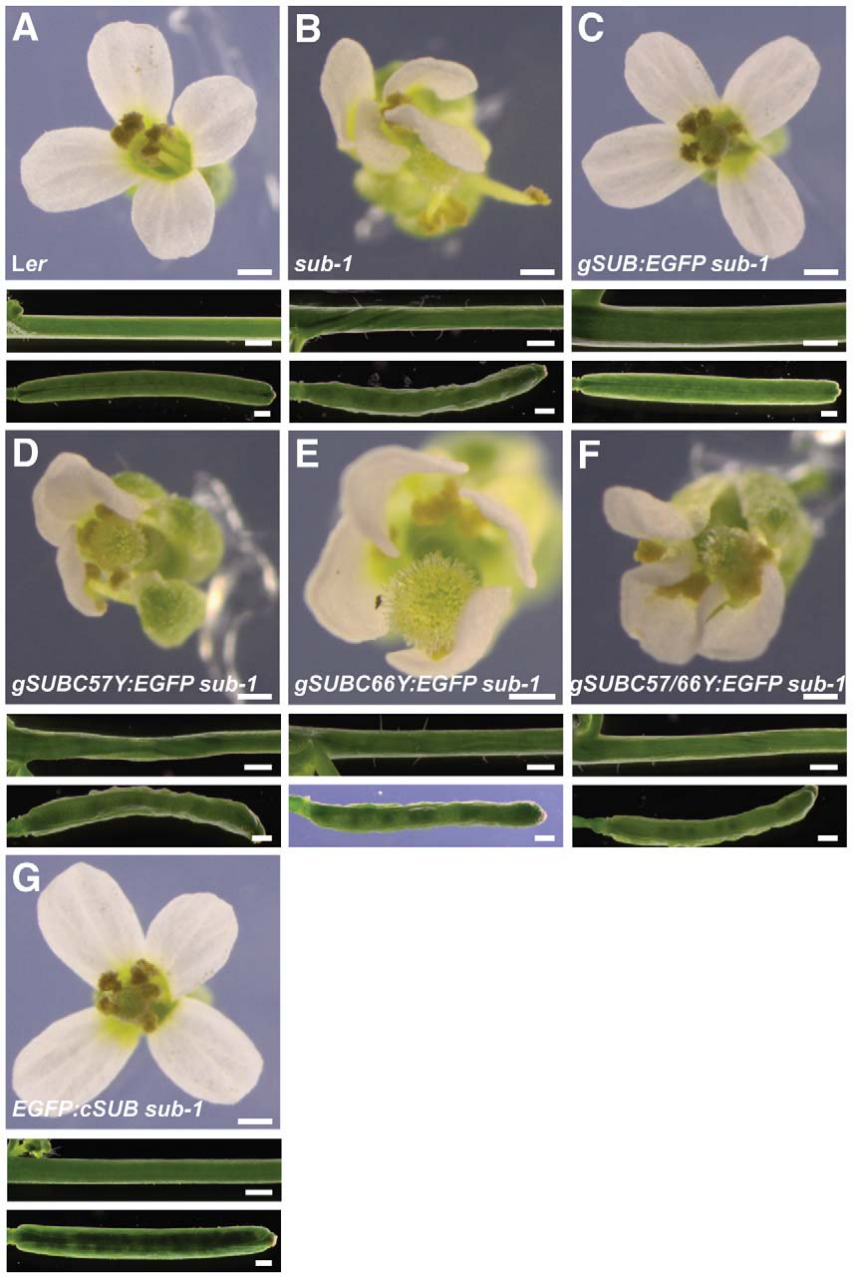
Functional analysis of different *SUB::SUB:EGFP*-based constructs. Flower morphology and stem and silique shape of *sub-1* plants carrying various reporter constructs. (A) Wild type (L*er*). (B) *sub-1*. (C) *SUB::gSUB:EGFP sub-1*. The plant appears wild type. (D) *SUB::gSUB_C57Y_:EGFP sub-1*. No rescue of the *sub-1* phenotype. (E) *SUB::gSUB_C66Y_:EGFP sub-1*. No rescue of the *sub-1* phenotype. (F) *SUB::gSUB_C57Y/C66Y_:EGFP sub-1*. No rescue of the *sub-1* phenotype. (G) *SUB::EGFP:cSUB sub-1*. A cDNA-based construct encoding a fusion of EGFP to the N-terminus of SUB. The plant looks wild type. Scale bars: 0.5 mm.

As expected, in vitro generated mutant constructs recapitulating the *sub-3* (V64M), *sub-4* (R599C) and *sub-10* (C57Y) mutations failed to rescue the *sub-1* mutant phenotype [25] (Figure 6D). Furthermore, *sub-1* plants carrying different deletion constructs (Figure 5A) all remained *sub-1* in appearance, indicating that each deletion eliminates *SUB* function (not shown). We also tested additional mutations. As outlined above the SUB/LRR structure model suggests that the C57 affected in *sub-10* and C66 in the SUB domain might form a disulphide bridge important for N-capping domain tertiary structure. We therefore tested if a corresponding substitution at C66 (C66Y) also impairs SUB function. To this end we used a tester construct that included genomic *SUB* coding sequence (see below). We found that the *SUB::gSUB_C66Y_:EGFP* construct failed to rescue *sub-1* plants (Figure 6E). Similarly, simultaneous alteration of both cysteines did not result in a functional *SUB::gSUB_C57/66Y_:EGFP* construct either (Figure 6F). The results suggest that C57 and C66 indeed play important roles for SUB function and are compatible with the hypothesis that the two cysteines participate in a critical disulphide bridge required for proper N-capping domain architecture. In this regard SUB appears to differ from BRI1, where mutating the equivalent two cysteines resulted in a functional protein [63], but behaves similarly to Cf-9, where equivalent mutations caused absence of Cf-9 activity [47].

In the ECDs of many LRR-RLKs, a cysteine pair is found proximal to the LRRs that appear to be functionally relevant, possibly for heterodimerization or as a component of a C-terminal capping domain involved in structural stabilization of the LRR domain [46], [49], [50]. While SUB lacks such a cysteine pair in its ECD it carries two neighboring cysteines just proximal to the transmembrane domain (C365/6) (Figure 5B). Transgenic *SUB::cSUB_C365/6A_:EGFP sub-1* plants, however, appeared wild type, indicating these cysteines do not contribute to SUB function (not shown).

**Figure 7:**
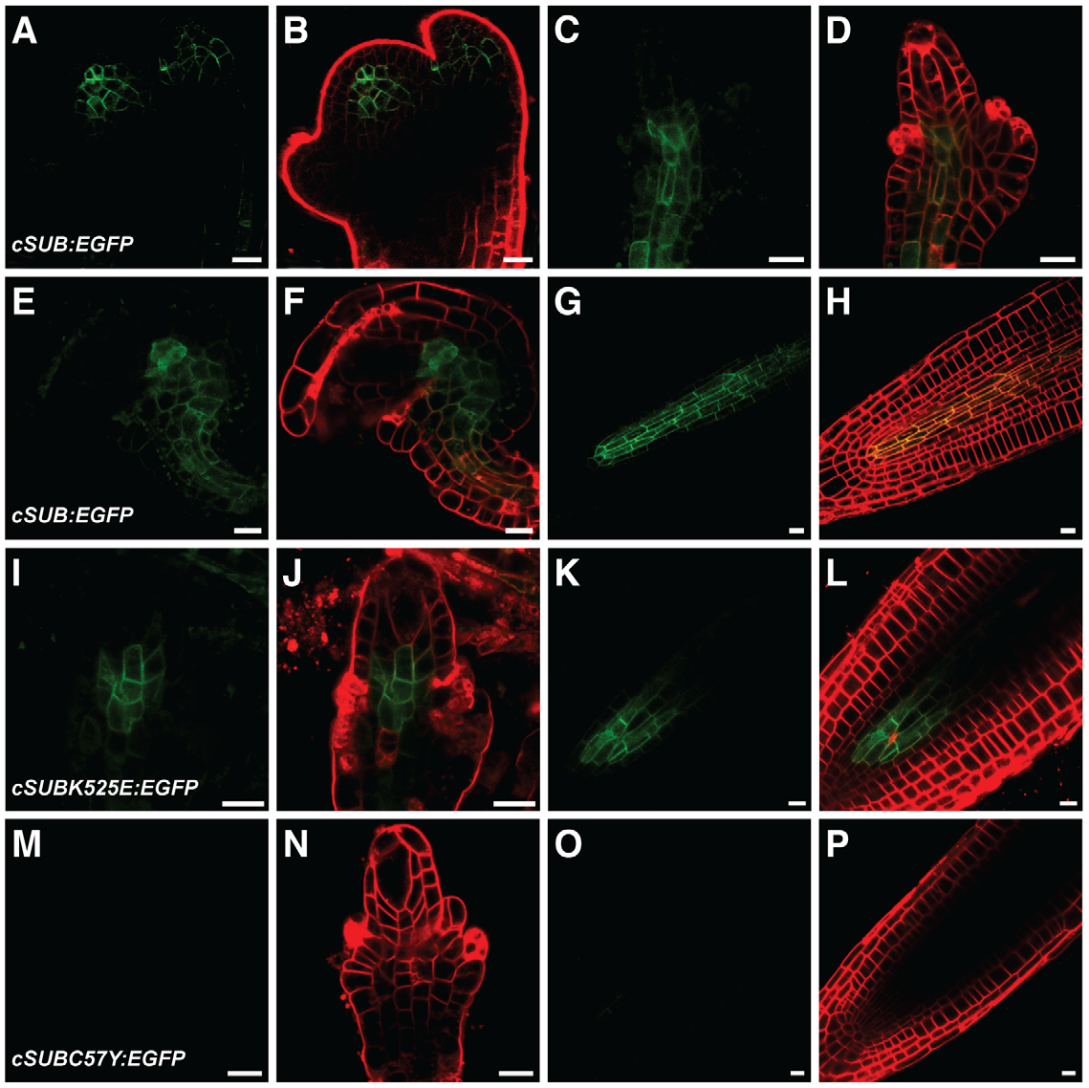
Expression analysis of different *SUB::cSUB:EGFP*-based reporters. Live confocal microscopy images obtained from L*er* plants carrying various cDNA-derived SUB:EGFP reporters. The FM4-64 stain was used to mark the outline of all cells in a tissue. Signals from the EGFP and FM4-64 channels are shown in green and red, respectively. Stage 3 floral meristems (A–B), stage 2-III (C–D, I–J, M–N) and 3-V/VI ovules (E–F), and roots from 4-day old seedlings (G–H, K–L, O–P) are depicted. (A–H) *SUB::cSUB:EGFP*. Weak signals are only detected in the center of the different organs. (I–L) *SUB::cSUB_K525E_:EGFP*. Weak signal that is noticeably restricted to the interior part of ovules and roots. (M–P) *SUB::cSUB_C57Y_:EGFP*. No detectable signal in ovules or roots. Scale bars: 10 µm.

All functional *SUB::cSUBmut:EGFP* reporters exhibited the expected signal strength and distribution (Figure 7 A–L). Surprisingly, however, and although we screened at least 100 primary transformants for each construct, we were unable to detect an EGFP signal in nonfunctional *SUB::cSUBmut:EGFP sub-1* plants (Figure 7M–P). This interesting finding could principally provide a coherent explanation for the observed homogeneity of the mutant phenotypes among the different *sub* alleles. In all tested alleles no mutant SUB protein would be present and thus all would exhibit a null phenotype. Further analysis, however, did not support this notion.

### *SUB* intronic sequences positively influence *SUB::SUB:EGFP* signal strength

The results presented above would provide a convenient explanation for the similar appearances of phenotypic *sub* alleles. Nevertheless, the findings also raise the question why no SUBmut:EGFP signal is detected. One explanation relates to a possible regulation of *SUB* expression by an autoregulatory feedback loop. Furthermore, the *SUB::cSUB:EGFP* reporter may not properly reflect the endogeneous SUB protein levels. Finally, since SUB has to pass through the secretory pathway, it is also conceivable that mutant SUB proteins get eliminated by the endoplasmic reticulum-mediated quality control (ERQC) system which disposes of misfolded and/or unassembled proteins by endoplasmic reticulum-associated degradation (ERAD) [68]–[71]. Recent reports provided compelling evidence that the bri1-5 and bri1-9 variants of the brassinosteroid receptor BRI1, carrying substitutions in their ECD domains, are retained in the ER and degraded by the ERAD system [63], [72], [73]. Another well-characterized example is the LRR-RLK EFR, a plant innate immune receptor involved in the perception of the bacterial translation elongation factor EF-Tu [74], [75].

**Figure 8:**
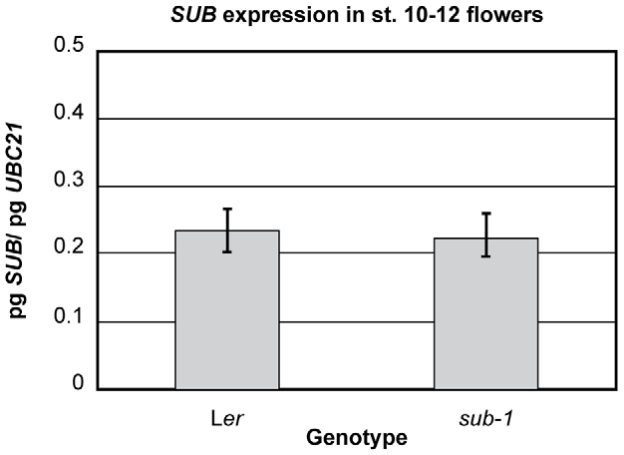
Quantitative expression analysis of *SUB* in *sub-1* flowers. Steady-state mRNA levels were measured in flower stage 10 to 12 tissue by quantitative real-time PCR using *SUB*-specific primers. Three biological replicates were used. *UBC21* mRNA was amplified in parallel and used for normalization.

First we tested if floral *SUB* expression in flowers is under the control of an autoregulatory feedback loop. To this end we performed quantitative real-time PCR (qRT-PCR) experiments using RNA isolated from wild-type and *sub-1* mutant flowers. As can be seen in Figure 8 we detected no evidence for a feedback loop regulating *SUB* transcription in flowers.

**Figure 9:**
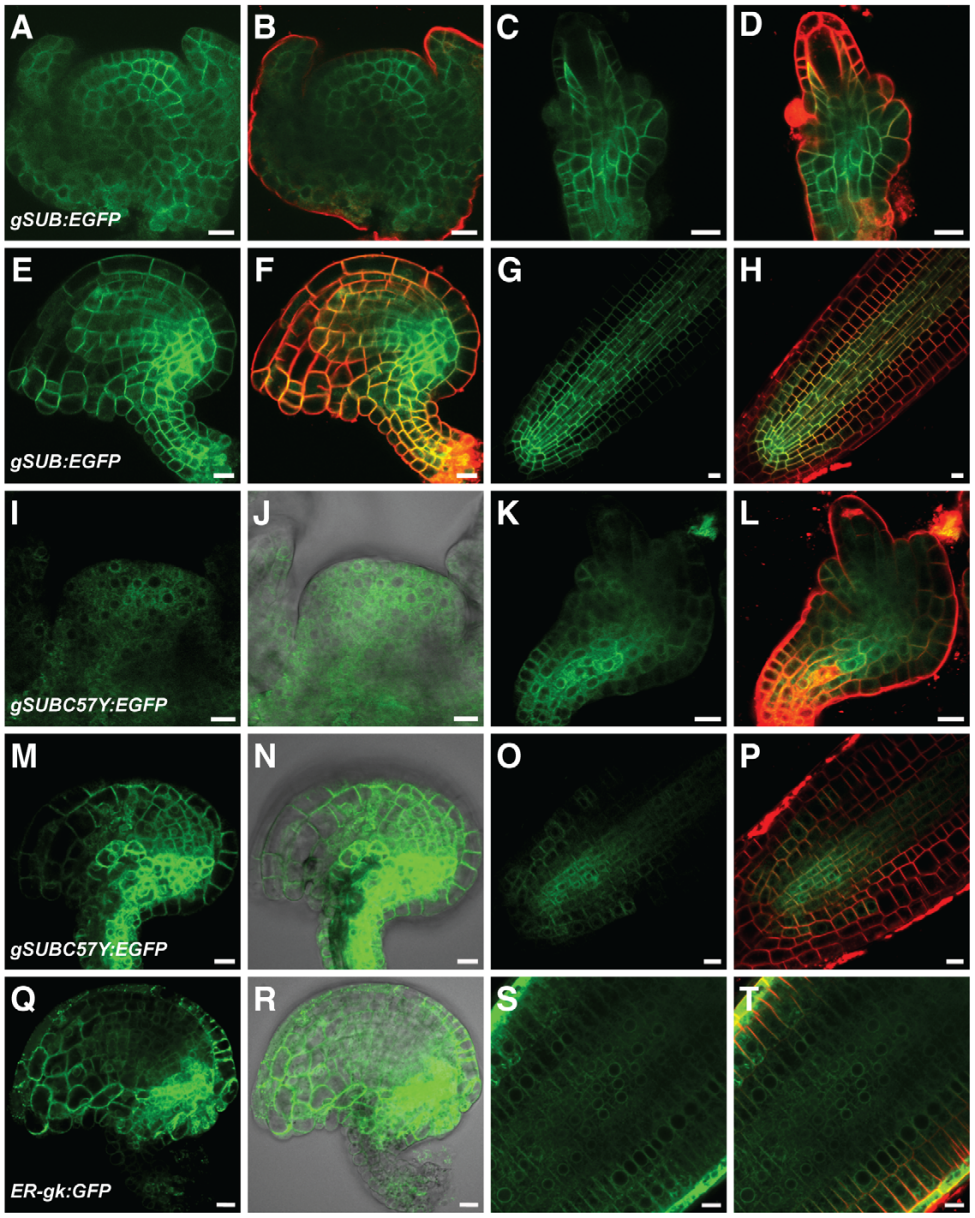
Expression analysis of *SUB::gSUB:EGFP*-based reporters. Live confocal microscopy images obtained from L*er* plants carrying various genomic DNA-derived SUB:EGFP reporters. The FM4-64 stain was used to mark the outline of all cells in a tissue. Signals from the EGFP and FM4-64 channels are shown in green and red, respectively. Differential interference contrast (DIC) photomicrographs are shown to outline the tissue (J, N, R). Stage 3 floral meristems (A–B, I–J), stage 2-III (C–D) stage 2-IV (K–L) and 3-V/VI ovules (E–F, M–N, Q–R), and roots from 4-day old seedlings (G–H, O–P, S–T) are depicted. Note the broad expression pattern, which includes the epidermis, in all examined tissues and with all tested reporter constructs. Signal tends to be stronger in interior tissues. (A–H) *SUB::gSUB:EGFP*. (I–P) *SUB::gSUB_C57Y_:EGFP*. Signal remains broadly detectable in tested tissues. Note the ER-like sub-cellular distribution (compare with Q–T). (Q–T) Line ER-gk CS16251 (Col) carrying plasmid ER-gk CD3-955. Control reporter exhibiting expression in the ER [98]. Scale bars: 10 µm.

Next we investigated the possibility of insufficient signal strength exhibited by the *SUB::cSUB:EGFP* and *SUB::cSUBmut:EGFP* reporters. As mentioned earlier the promoter elements present in these reporters correctly reflect the spatial expression pattern of *SUB* and the *SUB::cSUB:EGFP* construct can rescue the above-ground *sub* phenotype [28], [31]. Interestingly, however, while the spatial expression domain of *SUB* transcription extends to the periphery of several organs, such as ovules, floral meristems, and roots [25], [28], the *SUB::cSUB:EGFP* reporter only exhibits a weak signal in interior cells of those organs [31] (Figure 7A–H). In addition, a similar construct failed to rescue the root phenotype of *sub* mutants [76]. Thus, as previously suggested, *SUB* expression may be subject to complex control [31] and the findings raise the possibility that intronic sequences of *SUB* influence transcriptional or post-transcriptional processes. Thus, we generated a genomic *SUB* DNA construct that shares identical promoter elements with the cDNA-based reporter but included all *SUB* introns (*SUB::gSUB:EGFP*). Similar to its cDNA-based variant this construct could also rescue the *sub-1* phenotype (Figure 6C). Moreover, the new reporter indeed exhibited a broad signal that was detectable in the center and at the periphery of ovules and floral meristems (Figure 9A–F) (30/50 independent T1 lines). A similar staining pattern was also observed in roots (Figure 9G and H) confirming results obtained with a related construct [76]. The *SUB::gSUB:EGFP* reporter expression thus mimicked the *SUB* expression pattern as observed by *in situ* hybridization [25] and *SUB::GUS* studies [28], [31]. The signal tended to be somewhat stronger in internal tissues compared to more peripheral cell layers. These results support the notion that the limited spatial extension of detectable signal in *SUB::cSUB:EGFP* reporter lines is due to lower overall SUB:EGFP protein levels in comparison to the *SUB::gSUB:EGFP* lines. Hence, the generally stronger EGFP signals of *SUB::gSUB:EGFP* transgenes allows the monitoring of the relatively weaker signals exhibited by the peripheral cell layers of the assayed organs. Why *SUB::gSUB:EGFP* signal levels are higher remains to be determined but increased signal strength could be due to transcriptional or post-transcriptional effects. For example, the introns could carry one or several cis-acting elements positively regulating overall *SUB* transcript levels. Alternatively, an intron-dependent post-transcriptional mechanism could regulate SUB protein levels. It is known that introns can influence protein expression levels [77]–[79]. One explanation put forward suggests that upon splicing of an intron some factors remain bound to the exon-exon junction of the mRNA and the composition of such an mRNP may influence translation [77].

### Equivalent *SUB::cSUBmut:EGFP* and *SUB::gSUBmut:EGFP* constructs behave in a genetically identical manner

To examine if the observed differences in SUB:EGFP signal strengths between the *SUB::cSUB:EGFP* and *SUB::gSUB:EGFP* reporters could influence our genetic analysis we introduced into the *SUB::gSUB:EGFP* reporter by in vitro mutagenesis many of the different point mutations that are predicted to allow the translation of a full-length SUB protein but to result in either a functional or nonfunctional *SUB::cSUBmut:EGFP* constructs (Figure 5B). Subsequently, we assayed the ability of the different *SUB::gSUBmut:EGFP* reporters to rescue the *sub-1* phenotype. In all tested cases we analyzed at least 50 independent primary transformants. In summary, it was found that mutations rendering the *SUB::cSUBmut:EGFP* construct nonfunctional also resulted in nonfunctional *SUB::gSUBmut:EGFP* constructs, as corresponding *SUB::gSUBmut:EGFP sub-1* plants showed no rescue of the *sub* mutant phenotype (an example is given in Figure 6D). A similarly coherent relationship was observed for mutations that retained functionality of *SUB::cSUBmut:EGFP*. With G357S (*sub-16*) or T486A substitutions, both still resulted in corresponding functional genomic or cDNA-derived reporters (not shown). Thus, in terms of genetic complementation of *sub-1* plants the *SUB::cSUBmut:EGFP* and *SUB::gSUBmut:EGFP* constructs yielded identical results demonstrating that choice of construct did not influence the functional analysis in a noticeable manner.

Next we assayed signal strength and distribution of different *SUB::gSUBmut:EGFP* reporters. We analyzed at least 50 independent primary transformants for each construct in wild-type and *sub-1* backgrounds and continued with lines that showed detectable root signal for further analysis. Fewer lines exhibited detectable signal when compared to the wild-type *SUB::gSUB:EGFP* reporter (about 10/50 independent T1 lines vs 30/50) indicating overall weaker expression of the mutant reporters. In positive lines, signal could easily be detected in floral meristems, ovules, and roots, and with the expected spatial distribution at the organ level (an example is given in Figure 9I–P). However, the sub-cellular localization of the signal became broader (see below). These findings indicate that absence of an EGFP signal in *SUB::cSUBmut:EGFP* reporter lines indeed relates to the weaker overall expression levels of the cDNA-based reporter construct.

Interestingly, not all *sub-1* T1 lines carrying the *SUB::gSUB:EGFP* reporter exhibited detectable expression (20/50). Of these 20 T1 lines without apparent expression 15 still showed partial to complete rescue of the *sub-1* phenotype (not shown). This result indicates that very low levels of *SUB* expression provide sufficient *SUB* activity (see also below). Furthermore, it was previously shown that *SUB* acts in a non-cell-autonomous manner and regulates inter-cell-layer communication [31]. For example, specifically expressing *cSUB:EGFP* under the control of the epidermis-specific *ML1* promoter rescued the sub-epidermal defects in floral meristems of *sub-1* mutants. In addition, restricting *cSUB:EGFP* expression to the nucellus still allowed partial development of the integuments. The broad expression pattern of the *SUB::gSUB:EGFP* reporter was perhaps not to be expected in the light of those results. However, *BRI1* represents another example for a broadly expressed gene with a histogenic-layer-specific role in the regulation of cellular behavior at the tissue level [33]. Moreover, the *SUB::gSUB:EGFP* expression pattern provides a convenient explanation for the previously puzzling observation that *ML1::cSUB:EGFP* could also rescue the integument defects of *sub-1* [31]. Thus, an easy explanation for all observations is to propose that *SUB* regulates the behavior of cells within an L1-derived cell layer, such as the integuments of ovules [80], and between histogenic cell layers. One way this could be achieved is through the regulation of cell wall biology [27].

### Various mutant SUB variants are retained in the endoplasmic reticulum and degraded by ERAD

**Figure 10:**
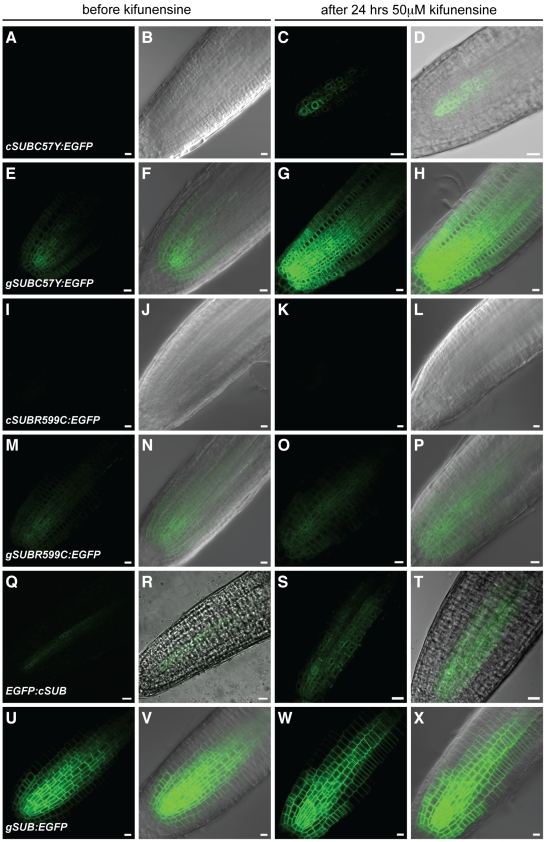
Effects of kifunensine treatments on the expression of different *SUB::c/gSUB:EGFP* reporters. Live confocal microscopy images from roots were generated using 4-day old Arabidopsis seedlings (L*er*) carrying different SUB:EGFP reporters. Signals from the EGFP channel are shown in green. Differential interference contrast (DIC) or brightfield photomicrographs are shown to outline root tissue (B, D, F, H, J, L, N, P, R, T, V, X). The same root before (A–B, E–F, I–J, M–N, Q–R, U–V) and after (C–D, G–H, K–L, O–P, S–T, W–X) 24-hrs treatment with 50 µM kifunensine. (A–D) *SUB::cSUB_C57Y_:EGFP*. Signal becomes detectable upon kifunensine treatment. Note ER-like pattern (compare with Figure 9S–T). (E–H) *SUB::gSUB_C57Y_:EGFP*. Signal becomes stronger upon kifunensine treatment. (I–L) *SUB::cSUB_R599C_:EGFP*. Absence of signal, irrespective of kifunensine treatment. (M–P) *SUB::gSUB_R599C_:EGFP*. Signal is easily detectable and not noticeably influenced by kifunensine treatment. (Q–T) *SUB::EGFP:cSUB*. Note the ER-like pattern (compare with Figure 9S–T). No change in signal intensity was observed upon kifunensine treatment. (U–X) *SUB::gSUB:EGFP*. The reporter signal does not change detectably upon treatment with kifunensine. Scale bars: 10 µm.

The detectable *SUB::gSUBmut:EGFP* reporter signals allowed the ready analysis of the subcellular distribution of the SUBmut:EGFP fusion proteins. In all instances, and irrespective of mutations in the extracellular or intracellular domains of SUB, a reticulated signal distribution typical for an ER-like distribution was observed, although minor signal was present at the plasma membrane as well (Figure 9I–T and Figure 10). Subcellular signal distribution was essentially identical to a functional reporter carrying an N-terminal fusion of EGFP to SUB (Figure 6G and Figure 10 Q–T) or to a bri1-5-GFP reporter [63]. The ER-related signal was never observed in wild-type *SUB::gSUB:EGFP* reporter lines (Figure 9A–H). Interestingly, the mutant *SUB::cSUBK525E:EGFP* reporter, which rescues the *sub-1* phenotype (Figure 4D), exhibited low expression levels analogous to *SUB::cSUB:EGFP* and showed no hint of ER localization (Figure 7I–L). Conversely, the overall lower signal strength of *SUB::gSUBmut:EGFP* reporters and their ER-like subcellular signal distribution are compatible with the notion that nonfunctional mutant SUB variants are partially retained in the ER by ERQC and eventually eliminated by ERAD. Similar scenario have been proposed for mutant variants of BRI1 and EFR [72], [74], [75].

To corroborate the notion that SUB receptors can be subject to ERQC/ERAD we treated wild-type or *sub-1* seedlings carrying the *sub-3* and *sub-10* mutations in the ECD of SUB:EGFP with kifunensine (Kif). We investigated the reporters *SUB::cSUB_V64M_:EGFP*, *SUB::gSUB_V64M_:EGFP*, *SUB::cSUB_C57Y_:EGFP*, and *SUB::gSUB_C57Y_:EGFP*. Furthermore, we included in our analysis a reporter corresponding to the *sub-4* mutation in the intracellular kinase domain (*SUB::cSUB_R599C_:EGFP*, *SUB::gSUB_R599C_:EGFP*). Kif is a potent inhibitor of glycoprotein processing mannosidase I in the ER and prevents ERAD of many terminally misfolded proteins [81], [82]. Expression analysis of the mutated SUB_mut_:EGFP fusion proteins in roots (three independent lines, 10 individual seedlings each) revealed that indeed signals could be observed for the cDNA-based ECD mutational variants *SUB::cSUB_V64M_:EGFP* and *SUB::cSUB_C57Y_:EGFP* upon Kif treatment (Figure 10A–D). The expression patterns were comparable to the related wild-type *SUB::cSUB:EGFP* reporter (Figure 7G and H) [31] and were irrespective of the genetic background (wild-type versus *sub-1*). The data suggest that *SUB::cSUBmut:EGFP* transgenes are principally transcribed (despite the absence of an EGFP-signal), that mutant variants of SUB:EGFP fusion proteins are subject to ERAD and that this process contributes to undetectable levels of fusion proteins derived from *SUB::cSUBmut:EGFP* reporters. In addition, the results provide indirect evidence that mature SUB receptor is glycosylated at the ECD. We also examined the roots of seedlings carrying genomic reporter variants (*SUB::gSUB_V64M_:EGFP*, *SUB::gSUB_C57Y_:EGFP*). The addition of Kif to seedling growth medium resulted in increased signal intensity in root tips (Figure 10E–H) substantiating the results obtained with the cDNA-based reporters.

As described, the C57Y and V64M variants carry alterations in the ECD of SUB. What happens to variants with a mutation in the intracellular domain? To address this question we assessed reporter lines carrying either *SUB::cSUB_R599C_:EGFP* or *SUB::gSUB_R599C_:EGFP* (*sub-4*-derived) reporters. No signal could be detected in roots of *sub-1* or wild-type plants carrying the *SUB::cSUB_R599C_:EGFP* reporter, irrespective of the addition of Kif (Figure 10I–L) (8 independent T1 lines tested). Individual seedlings of three independent lines (10 seedlings per line) carrying the genomic *SUB::gSUB_R599C_:EGFP* variant exhibited a signal in root tips that stayed constant upon Kif treatment (Figure 10M–P). Interestingly, signals of the *SUB::c/gSUB_R599C_:EGFP* reporters exhibited a similar sub-cellular distribution to the one exhibited by SUBmut:EGFP fusion proteins with defects in their ECDs. The results suggest that the *sub-4* variant of SUB, which carries an altered cytoplasmic kinase domain, is not measurably affected by a Kif-dependent process. Still, the undetectable signal of the *SUB::cSUB_R599C_:EGFP* reporter in the absence of Kif and the ER-like distribution of the *SUB::gSUB_R599C_:EGFP* signal suggests that a Kif-independent mechanism of ERQC/ERAD is involved in limiting the amount of *sub-4*-like SUB variants on the cell surface. The process likely depends on the recognition of the misfolded kinase domain by cytoplasmic chaperones involved in ERQC/ERAD, such as certain 70 kDa heat-shock proteins (Hsp70s), and associated factors [70].

**Figure 11:**
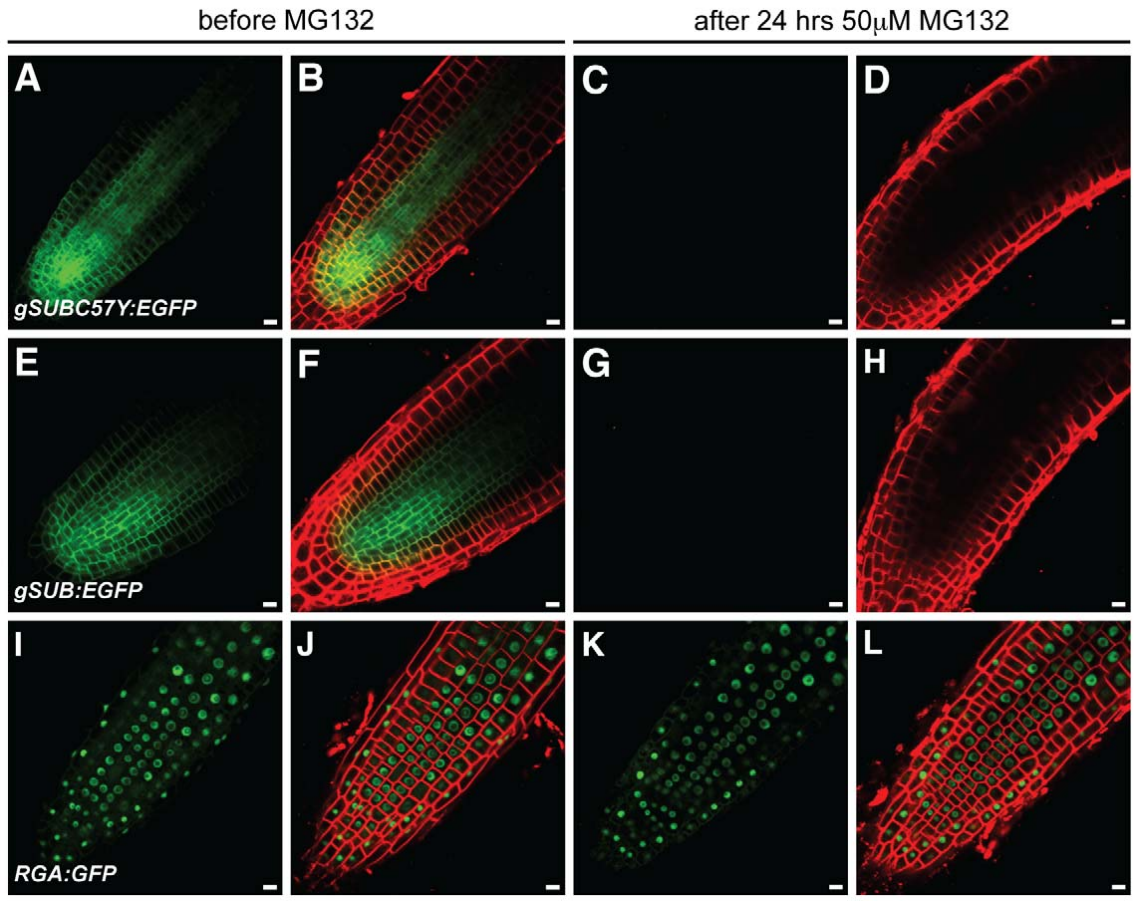
Effects of MG132 treatments on the expression of different *SUB::gSUB:EGFP* reporters. Live confocal microscopy images from roots were generated using 4-day old Arabidopsis seedlings (L*er*) carrying different SUB:EGFP reporters. The same root is shown before (A–B, E–F, I–J) and after (C–D, G–H, K–L) 24-hrs treatment with 50 µM MG132. The FM4-64 stain was used to mark the outline of all cells in a tissue. The signals from the EGFP and FM4-64 channels are shown in green and red, respectively. (A–D) *SUB::gSUB_C57Y_:EGFP*. (E–H) *SUB::gSUB:EGFP*. (I–L) A *RGA::RGA:GFP* reporter that served as control [93]. Note that signal persisted after MG132 treatment (K–L). Scale bars: 10 µm.

Unfortunately, we could not test the involvement of the proteasome in SUB-related ERAD by applying the proteasome inhibitor MG132 as *SUB* undergoes MG132-sensitive posttranscriptional regulation in root tips [31]. Seedlings carrying *cSUB:EGFP* reporters start to lose detectable SUB:EGFP signal after three hours of treatment with MG132 [31]. This phenomenon is also observed for *gSUB:EGFP*-based reporter constructs and is irrespective of whether wild-type or mutant SUB:EGFP fusion proteins are examined (Figure 11).

Taken together the combined data suggest that the SUB receptor is subject to ERQC similar to other plant receptor kinases, such as BRI1 or ERF. The results further indicate that the phenotypic similarity of different *sub* alleles is not due to absence of mutant SUB protein from cells. Rather, different tested phenotypic mutations all result in mutant SUB proteins that are likely present at the plasma membrane but lack SUB activity. It is formally possible that at least some of the defective SUB proteins have residual activity but are present at insufficient levels at the cell surface. Although we cannot exclude this possibility we deem it unlikely as, for example, reporter lines expressing a N-terminal fusion of EGFP to SUB (*SUB::EGFP:cSUB sub-1*) show rescue of the *sub-1* phenotype but still weak signal of the EGFP:SUB fusion protein (4/18 T1 lines showed signal, 16 lines showed phenotypic rescue), which presented subcellular distribution pattern similar to the various SUBmut:EGFP fusion proteins (Figure 10Q–T). In addition, we have observed effective rescue of transgenic *sub-1* plants carrying alternatively *35S::SUB:EGFP*, *SUB::c/gSUB:*EGFP or functional *SUB::c/gSUBmut:EGFP* constructs, which showed no apparent signal (not shown, see above). These findings indicate that several types of functional transgenes with either likely altered ECDs or undetectable expression levels can provide sufficient SUB activity.

The experiments outlined above suggest a complex control of SUB protein levels. First, a mechanism is in place that regulates the spatial and temporal transcription pattern of *SUB*. The different results obtained with various *SUB:EGFP* and *SUBmut:EGFP* reporter constructs imply that additional processes regulate overall SUB protein accumulation. One mechanism depends on the presence of *SUB* intronic sequences and regulates SUB levels either in a transcriptional or post-transcriptional fashion, by for example influencing *SUB* mRNA stability and/or translation. During their passage through the secretory pathway SUB proteins are subject to ERQC. Finally, in roots there is evidence for a feedback mechanism regulating differential cell-type-specific SUB accumulation in the root epidermis [76]. We could confirm cell-type specific differences in SUB:EGFP accumulation in the root epidermis (not shown), however, in all investigated lines *SUB::gSUB:EGFP*-derived signals appeared uniform across cells within cell layers in floral meristems and ovules (Figures 7 and 9). Furthermore, assessment of overall *SUB* expression levels in *sub* flowers via qRT-PCR did not provide evidence for a feedback loop regulation *SUB* transcription (Figure 8). The combined results indicate that cell-type-specific feedback mechanisms regulating SUB accumulation may be specific to the root.

### *ERECTA* influences the *sub* phenotype

**Figure 12:**
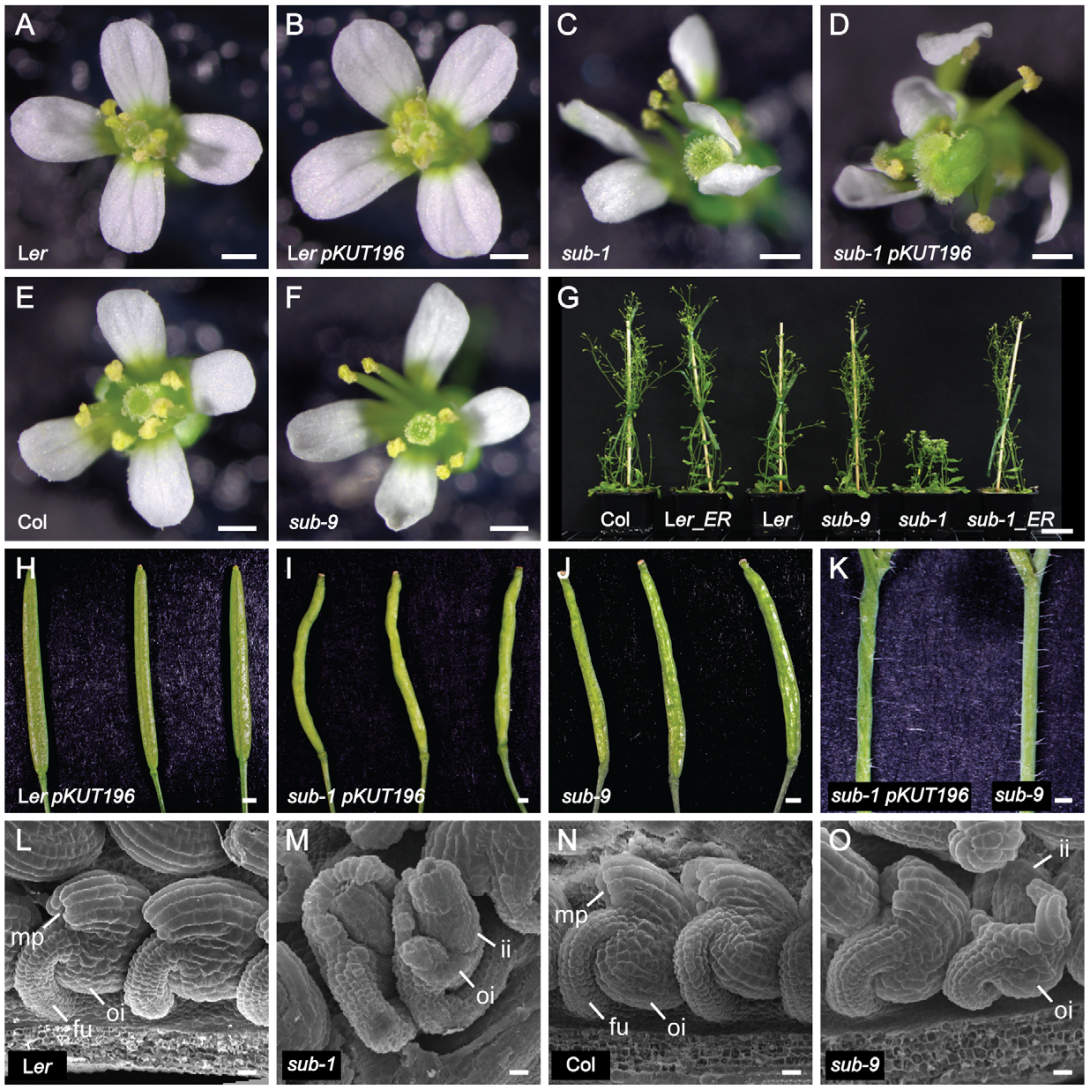
Analysis of *sub* above-ground morphology in the presence of functional *ERECTA*. Phenotypic comparison of wild-type, *sub* mutant and Col *ER*-containing pKUT196 transgenic plants. (A–F) Flower morphologies. (A) Wild-type L*er*. (B) Transgenic L*er* pKUT196. (C) *sub-1* mutant. (D) Transgenic *sub-1* pKUT196. Note the irregular *sub*-like appearance of floral organs. (E) Col wild-type. (F) Col *sub-9* mutant. Note the near wild-type appearance of floral organs. (G) Plant height comparisons of pKUT196 transgenic plants in comparison to wild-type and mutant reference lines. (H–J) Morphology of maturing siliques. Three different specimens per genotype are shown. (H) L*er* pKUT196. (I) *sub-1* pKUT196. (J) Col *sub-9* mutant. (K) Stem segments from *sub-1* pKUT196 and *sub-9* plants. (L–O) Comparison of ovule morphology by scanning electron microscopy. (L) L*er*. (M) *sub-1*. (N) Col wildtype. (O) Col *sub-9*. Abbreviations: fu, funiculus; ii, inner integument; mp, micropyle; oi, outer integument. Scale bars: (A–F, H–K) 0.5 mm, (G) 3 cm, (L–O) 20 µm.

Certain aspects of the *sub* phenotype depend on the genetic background [25]. For example, internode elongation and stem morphology is essentially normal in the null alleles *sub-6* and *sub-9* (T-DNA insertions in Col background) compared to the marked effects seen in null alleles *sub-1* or *sub-2* (in L*er* background). By contrast, *sub*-related defects in ovule development and root hair patterning are comparable in the L*er* and Col backgrounds (Figure 12) [27], [28]. The laboratory strain L*er* is characterized by a large number of polymorphisms when compared to other regularly used accessions such as Col [83], [84] (www.1001genomes.org). Segregation analysis in *sub-1* L*er*/Col mapping populations corroborated that *ERECTA* (*ER*) or a gene closely linked to *ER* could influence the *sub* phenotype (not shown).

To test if *ER* is responsible for the phenotypic differences between *sub* null alleles in the L*er* and Col backgrounds we transformed *sub-1* plants with pKUT196, a plasmid carrying 9.3 kb of genomic Col-0 DNA spanning the entire *ER* locus [16], [85], and asked how the addition of a functional *ER* copy altered the *sub-1* phenotype. As can be seen in Figure 12 *sub-1* plants carrying the *ER* transgene showed essentially normal internode length and accordingly, plant height. The *ER* transgene, however, failed to rescue other characteristics of *sub-1* mutants, such as defective flower and silique morphology, ovule development and stem twisting. These results show that the strong reduction in plant height of *sub* alleles in the L*er* background is caused by a synergistic effect between the *sub* and *er* mutations.

We cannot rule out that the observed synergism is a simple side-effect of the cellular *er* phenotype. However, it seems unlikely given that other aspects of the *sub* phenotype, such as integument or flower morphology, are insensitive to *ER*. Thus, we currently favor the notion that ER and SUB influence each other during stem development, although how remains to be determined. It is conceivable that the two RLKs converge in their signaling, either directly at the receptor level, with for example SUB and ER being part of the same protein complex, or at one or several steps further downstream in the signaling mechanism.

It was previously assumed that the reduction in plant height of *sub* mutants was at least in part due to stem twisting. Our data show that the control of internode length and stem shape by *SUB* can be genetically uncoupled indicating that *SUB* regulates the two processes through separate mechanisms. This raises the possibility that other aspects of the *sub* phenotype may also have a more complex basis than originally appreciated. Finally, given that *sub-9* (Col) stems appear wild type, and *sub-9* flower and silique defects are greatly reduced, the data also indicate that Col carries additional modifiers affecting the *SUB*-dependent regulation of stem, flower, and silique form. Future genetic and molecular analysis of these modifiers will likely identify interesting novel components involved in *SUB* signaling.

## Materials and Methods

### Plant work

*Arabidopsis thaliana* (L.) Heynh. var. Columbia (Col-0) and var. Landsberg (*erecta* mutant) (L*er*) were used as wild-type strains. The *sub-1* to *sub-5* mutants (L*er* background) were described previously [25] as was *sub-6* (Col background) [28]. Plants were grown in a greenhouse under Philips SON-T Plus 400 Watt fluorescent bulbs on a long day cycle (16 hrs light). Dry seeds were sown on soil (Patzer Einheitserde, extra-gesiebt, Typ T, Patzer GmbH & Co. KG, Sinntal-Jossa, Germany) situated above a layer of perlite, stratified for 4 days at 4°C and then placed in the greenhouse. The plants were kept under a lid for 7–8 days to increase humidity and support equal germination. The EMS-induced mutations *sub-10* to *sub-20* were identified in conjunction with the Seattle Arabidopsis TILLING facility (http://tilling.fhcrc.org/files/Welcome_to_ATP.html/) [55]. Tilling was performed in a Col line that carries the fast-neutron-induced *er-105* mutation [16]. Three different 0.8 to 1 kb genomic regions spanning the SUB/LRR, PRR, and kinase domains were screened. The mutations in homozygous form were confirmed in M3 plants by sequencing. Mutant plants were outcrossed to L*er* before analysis. Several T-DNA insertion lines were received from the SALK collection [86] (*sub-6*, SALK_011495, Col), the Wisconsin collection [87] (T28P6.18, *sub-8*, Ws-2, gift of F. Tax, University of Arizona), and the Syngenta Arabidopsis Insertion Library (SAIL) [88] (*sub-9*, SAIL_1158_D09, Col).

### Recombinant DNA work

For DNA and RNA work standard molecular biology techniques were used [89]. PCR-fragments used for cloning were obtained using either PfuUltra high-fidelity DNA polymerase (Stratagene) or TaKaRa PrimeSTAR HS DNA polymerase (Lonza, Basel, Switzerland). PCR fragments were subcloned into pJET1.2 using the CloneJET PCR cloning kit (Fermentas) or into pCRII-TOPO (Invitrogen). All PCR-based constructs were sequenced. The plasmid pCAMBIA2300 was used as binary vector (www.cambia.org). Information regarding the primers is given in Table S1.

#### Wild-type SUB::c/gSUB:EGFP reporter constructs

The SUB::cSUB:EGFP reporter construct was described previously [31]. To generate the SUB::gSUB:EGFP construct L*er* genomic DNA was used as template and amplified with primers SUB-Genomic2/F and SUB-Genomic2/R. The PCR fragment was reamplified by using primers SUB_cmyc_F, SUB_cmyc_R and cloned into pJET1.2 by blunt end cloning generating pJET1.2gSUB. The insert was released by an *Asc*I/*Aat*II restriction digestion and subcloned into *Asc*I/*Aat*II digested SUB::cSUB:EGFP (in pCAMBIA2300), thereby replacing cSUB with gSUB and generating SUB::gSUB:EGFP. The vector 35S::SUB:3xmyc pCAMBIA2300 was generated as follows. To clone the 35S promoter adjacent to SUB:3×myc plasmid SUB:3×myc pCAMBIA2300 was used. The 35S fragment was obtained by digesting vector pART-7 first with *Not*I and, then blunt ending using T4 DNA polymerase followed by digestion after gel purification with *Xba*I. To generate compatible end for the 35S insert vector SUB:3×myc pCAMBIA2300 was digested first with *Bam*HI, made blunt with T4 DNA polymerase, and subsequently gel purified and digested with *Spe*I generating 35S::SUB:3×myc pCAMBIA2300.

#### Wild-type N-terminal tagged SUB::EGFP:cSUB fusion construct

The DNA fragments representing the signal peptide (SP) sequence of SUB and the coding sequence of EGFP were fused via overlapping PCR. The resulting SP:EGFP fragment was cloned into cSUB:3×myc (lacking the SP) in pCRII-TOPO by *Bam*HI digestion resulting in SP:EGFP:cSUB:3×myc pCRII-TOPO. The SP:EGFP:cSUB fragment was amplified using primers SUB_cmyc_F and Sig:SUB_Xba1_R and subcloned into binary vector cSUB:EGFP pCAMBIA2300 [31] replacing SUB:EGFP by *Asc*I/*Xba*I restriction digestion. Then the 3.5 kb *SUB* promoter fragment was subcloned from SUB::cSUB:EGFP by *Kpn*I/*Asc*I digestion resulting in SUB::SP:EGFP:cSUB in pCAMBA2300 (SUB::EGFP:cSUB).

#### Mutant SUB::c/gSUBmut:EGFP reporter constructs

To design the five truncated versions of *SUB*, a PCR amplification based approach was used. The plasmid pCRII *SUB*:3×myc [31] served as a template. The 35S::SUB:3×Cmyc pCAMBIA2300 [31] plasmid used as a backbone. Full length *SUB* was replaced by truncated versions of *SUB* using *Asc*I and *Aat*II sites. For the SUBΔTM–Intra primers SUB-Cmyc-F, and 35S-extra-myc-rev were used. To construct 35S::SUBΔIntra:3×myc primers SUB-Cmyc-F and 35S-TMmyc-rev were used. The 35S::SUBΔCD:3×Cmyc plasmid was constructed using primers SUB:3×myc-F and JuxtraAatII-R. PCR fragments were treated with *Asc*I and *Aat*II and cloned into correspondingly digested 35S::SUB:3×myc pCAMBIA2300. To generate SUBΔECD:3×Cmyc primers SUB-Cmyc-F and Alalinksignal-rev were used to amplify the signal sequence of *SUB*. Primers Alalink-TM-intra-for and SUB-Cmyc-R were used to amplify the TM-intracellular domain fragment. After gel purification an overlap PCR was setup to generate a fragment carrying the signal peptide and the TM-intracellular domain but lacking the ECD. This fragment was digested with *Asc*I and *Aat*II and cloned into 35S::SUB:3×myc pCAMBIA 2300. To generate SUBΔECD-TM:3xCmyc the entire intracellular region was amplified using primers AscIntra-F and SUB-Cmyc-R pair, digested with *Asc*I and *Aat*II, and cloned into 35S::SUB:3×myc pCAMBIA 2300. To clone the truncated SUB versions into a SUB promoter plasmid, the five truncations were digested with *Asc*I/*Aat*II respectively and cloned into *Asc*I/*Aat*II digested vector pSUB::SUB:EGFP [31].

All point mutations were generated using the QuikChange II XL site-directed mutagenesis kit according to the manufacturer's recommendations (Agilent Technologies). For the cDNA-based cSUBmut versions 35S::SUB:3×myc pART7 was used as template [25] while for the genomic gSUBmut versions, pJET 1.2 gSUB was employed as template. The sequence of the mutagenized constructs was verified by sequence analysis. The cSUBmut variants were amplified from in vitro mutagenized 35S::SUB:3xmyc pART7 plasmids using primers SUB_cmyc_F, SUB_cmyc_R and subcloned into SUB::cSUBΔECD:EGFP (in pCAMBIA 2300 binary vector), thereby replacing the cSUBΔECD fragment, by *Asc*I/*Aat*II restriction digestion. The gSUBmut variants were subcloned from in vitro mutagenized pJET1.2gSUB into SUB::cSUB:EGFP using *Asc*I/*Aat*II restriction digestion, replacing cSUB with gSUBmut.

### Generation of transgenic plants

Wild-type and *sub-1* plants were transformed with different constructs using Agrobacterium strain GV3101/pMP90 [90] and the floral dip method [91]. Transgenic T1 plants were selected on Kanamycin plates (50 µg/ml) and subsequently transferred to soil for further inspection.

### Quantitative real-time PCR analysis

Tissue preparation, RNA isolation, and quantitative real-time PCR on a Roche LightCycler using the SYBR Green I detection kit from Roche was performed as described previously [27] with three biological replicates. Amplification of *UBC21*/At5g25760 served as a normalization control [92]. Using the comparative Ct method, all gene expression levels were calculated relative to *UBC21*.

### Kifunensine and MG132 treatments

Transgenic seeds containing various *SUB::SUB:EGFP* or *SUB::SUBmut:EGFP* transgenes were germinated on vertical minimal media plates. After five days whole seedlings were transferred to 24-well suspension-culture-plates (Cellstar®, Greiner Bio-one GmbH, Frickenhausen, Germany), the bottom of the wells coated with full-strength MS agar containing 50 µM kifunensine (Enzo Life Sciences, Lausen, Switzerland), and incubated at standard growth conditions for 24 hours. Reporter expression was subsequently assayed as described below. Seedlings were placed in 24-well culture plates and treated for 24 hours with 50 µM MG132 in liquid full-strength MS medium as outlined previously [31]. The *RGA::RGA:GFP* control line was described earlier [93].

### Homology modeling

Homology modeling was made by submitting the entire SUB protein sequence to the web-based Swiss-Model workspace (http://swissmodel.expasy.org/workspace/) [43] using automated mode and default settings. The algorithms generated two models, one for the LRRs and one for the kinase domain. The templates were 1ogqA and 2qkwB for the LRRs and the kinase domain, respectively. The 1ogqA protein data bank (PDB, http://www.rcsb.org/pdb/home/home.do) entry corresponds to the structure of polygalacturonase-inhibiting protein 2 (PGIP2), a leucine-rich repeat protein involved in plant defense [44]. Sequence identity was 24% with an E value of 3.3E-32. The 2qkwB entry relates to tomato Pto kinase [45]. Sequence identity was 28.3% with an E value of 0. Identical results were obtained by submitting just the LRR and kinase domain sequences to the Swiss-Model website. Models were saved as protein data bank (.pdb) files and molecular graphics images were produced using the UCSF Chimera package [94]. PDB files of the two homology models are given in Datasets S1 and S2. Quality assessment of the models was done using ANOLEA [95], QMEAN [96] and DFire [97] using the structure assessment tools of the Swiss-Model workspace website. The results are given in Figures S3 and S4.

### Analysis of natural variation at the STRUBBELIG protein level

We downloaded the TAIR10 genome matrix containing 80 *Arabidopsis thaliana* accessions (MPICao2010) from the http://1001genomes.org/ website. These sequence data were produced by the Weigel laboratory at the Max Planck Institute for Developmental Biology. We extracted and translated the corresponding *STRUBBELIG* (At1g11130) sequences by loci using in-house software. The protein alignment was computed by ClustalW (http://www.ebi.ac.uk/Tools/msa/clustalw2/).

### Microscopy and art work

Confocal laser scanning microscopy using EGFP and the stain FM4-64 was performed as reported previously [27], [31].

## Supporting Information

Figure S1 **Protein sequence alignment of the Arabidopsis SRF family.** Highlights the different predicted structural motifs of SUB, the positions of the *sub* mutations described in this paper, and the positions of amino acid substitutions in SUB found in some naturally occurring Arabidopsis accessions (underlined).
(PDF)

Figure S2 **SUB protein sequence alignment from 57 different Arabidopsis accessions.** ClustalW alignment. Depicts some of the natural variation in SUB. At1g11130.1_REF corresponds to the TAIR10 reference sequence of SUB.
(PDF)

Figure S3 **Quality assessment of the SUB LRR homology model.**
(PDF)

Figure S4 **Quality assessment of the SUB kinase domain homology model.**
(PDF)

Table S1 **Primers used in this study.**
(DOC)

Dataset S1 **PDB file of the homology model of the SUB LRR region.**
(PDB)

Dataset S2 **PDB file of the homology model of the SUB kinase domain region.**
(PDB)
