# Supplementary material for: Structure-Function Analysis of STRUBBELIG, an Arabidopsis Atypical Receptor-Like Kinase Involved in Tissue Morphogenesis
Source: PLoS One. 2011 May 16;6(5):e19730. doi: 10.1371/journal.pone.0019730 (PMC3095605; doi:10.1371/journal.pone.0019730)
Supplement: Figure S1 — Protein sequence alignment of the Arabidopsis SRF family. Highlights the different predicted structural motifs of SUB, the positions of the sub mutations described in this paper, and the positions of amino acid substitutions in SUB found in some naturally occurring Arabidopsis accessions (underlined). (PDF) [file pone.0019730.s001.pdf]

```
                            |--------- SUB domain ----
           |----------- SP -----------|    |-ααααααααααα------- loop -
                                           sub-6/T-DNA
                F9L G11V          S22T     R29*
                 |   |              |       |
SUB/SRF9    1 MSFTRWE---VFFG-LSVLALTMP-FSAGVTNLRDVSAINNLYITLGAP-SLHHWLAFGG
SRF1        1 MRSGRDN-NICFLGFLSFALISLPSLSLALTNPDDVAAINSLFLALESP-LLPGWVASGG
SRF3        1 MAAKRSIYCLLLLPLLLSLLIWIPSISLAATNPDDVAAINGLFAALGAP-VLPGWIASGG
SRF6        1 ----MRENWAVVALFTLCIVGFELRFIHGATDASDTSALNTLFSGMHSPAQLTQWTAAAG
SRF7        1 ----MTENRVVLALLILCIVGFEPSFIHGATDSSDTSALNIMFSSMNSPGQLSQWTASGG
SRF8        1 MAIGDRAMFTVLLLFIASISGFS--VVRCVTDPSDVQALQVLYTSLNSPSQLTNWKNGGG
SRF4        1 ----MGPNLQRIVLVFIACFGIFTSVVLAKTDSQDVSALNDAYKSMNSPSKLKGWSSSGG
SRF5        1 -------MTQKLVRLVIVSLAITVTLLQAKTDNQEVSALNVMFTSLNSPSKLKGWKANGG
SRF2        1 ----MKTKQQLRFLATILLTTILFVLAKTDTDPLEVLALQDLYKSLRNPEQLRGWRLEGG

           -- SUB domain -------------------|
           ---------βββ|--------- LRRa ---------||-------- LRRI -------
             sub-10    sub-3  sub-11
             C57Y      V64M   S69L
              |         |      |
              |   xWxGVxC      |
SUB/SRF9   55 DPCGEKWQGVVCDSSNITEIRIPGMKVGGGLS-DTLADFSSIQVMDFSSNHISGTIPQAL
SRF1       59 DPCGESWQGVLCNASQVETIILISANLGGELG-VGLNMFTSLKAMDFSNNHIGGSIPSTL
SRF3       60 DPCGEAWQGIICNVSDIISITVNAANLQGELG-DNLAKFTSIRGIDFSNNRIGGSIPSTL
SRF6       57 DPCGQNWRGVTCSGSRVTQIKLSGLELSGTLGGYMLDKLTSLTELDLSSNNLGGDLPYQF
SRF7       57 DPCGQNWKGITCSGSRVTQIKLPSLGLSGSLG-FMLDKLTSVTEFDMSNNNLGGDLPYQL
SRF8       59 DPCGESWKGITCEGSAVVTIDISDLGVSGTLG-YLLSDLKSLRKLDVSGNSIHDTLPYQL
SRF4       57 DPCGDSWDGITCKGSSVTEIKVSGRGLSGSLG-YQLGNLKSLTYLDVSKNNLNGNLPYQL
SRF5       54 DPCEDSWEGVKCKGSSVTELQLSGFELGGSRG-YLLSNLKSLTTFDLSKNNLKGNIPYQL
SRF2       57 DPCGEAWLGISCSGSSIVDLQLRELKLLGSLG-NQLQHLHNLKILDVSFNNLEGEIPFGL

           -||------- LRRII ------||------- LRRIII -------||----- LRRIV
                sub-1
                S121--*
                 |
SUB/SRF9  114 PSSIRNLSLSSNRFTGNIPFTLSFLSDLSELSLGSNLLSGEIPDYFQQLSKLTKLDLSSN
SRF1      117 PVSLQNLFLSGNNFTGTIPESLSSLKSLSVMSLNNNLLSGKIPDVFQDLGLMINIDLSSN
SRF3      118 PVTLQHFFLSANQFTGSIPESLGTLSFLNDMSLNDNLLSGELPDVFQNLVGLINLDISSN
SRF6      116 PPNLQRLNLANNQFTGAASYSLSQITPLKYLNLGHNQFKGQIAIDFSKLDSLTTLDFSFN
SRF7      115 PPNLERLNLANNQFTGSAQYSISMMAPLKYLNLAHNQLK-QLAIDFTKLTSLSILDLSSN
SRF8      117 PPNLTSLNLARNNLSGNLPYSISAMGSLSYMNVSGNSLTMSIGDIFADHKSLATLDLSHN
SRF4      115 PDKLTYLDGSENDFNGNVPYSVSLMNDLSYLNLGRNNLNGELSDMFQKLPKLETIDLSSN
SRF5      112 PPNIANLDFSENELDGNVPYSLSQMKNLQSINLGQNKLNGELPDMFQKLSKLETLDFSLN
SRF2      115 PPNATHINMAYNNLTQSIPFSLPLMTSLQSLNLSHNSLSGPLGNVFSGLQ-IKEMDLSFN

           -----------||-------- LRRV -------||----- LRRVI ---------|
                            sub-9/T-DNA                 sub-12
                            D196--*                     S222L
                             |                            |
SUB/SRF9  174 ILEGHLPSSMGDLASLKILYLQDNKLTGTLDVIEDL-FLTDLNVENNLFSGPIPPNLLKI
SRF1      177 NLSGPLPPSMQNLSTLTSLLLQNNHLSGELDVLQDL-PLKDLNVENNLFNGPIPEKLLSI
SRF3      178 NISGTLPPSMENLLTLTTLRVQNNQLSGTLDVLQGL-PLQDLNIENNLFSGPIPDKLLSI
SRF6      176 SFTNSLPATFSSLTSLKSLYLQNNQFSGTVDVLAGL-PLETLNIANNDFTGWIPSSLKGI
SRF7      174 AFIGSLPNTCSSLTSAKSIYLQNNQFSGTIDILATL-PLENLNIANNRFTGWIPDSLKGI
SRF8      177 NFSGDLPSSLSTVSTLSVLYVQNNQLTGSIDVLSGL-PLKTLNVANNHFNGSIPKELSSI
SRF4      175 QLTGKLPQSFANLTGLKTLHLQENQFKGSINALRDLPQIDDVNVANNQFTGWIPNELKNI
SRF5      172 KLSGKLPQSFANLTSLKKLHLQDNRFTGDINVLRNL-AIDDLNVEDNQFEGWIPNELKDI
SRF2      174 NLTGDLPSSFGTLMNLTSLYLQNNRLTGSVIYLADL-PLADLNIEDNQFSGIIPSHFQSI
```

```
                                            sub-13        sub-14     A285D
                                            P258L         P272S     F284L|
                                            |             |            ||
SUB/SRF9  233 PNFKKDGTPFN-----TSIITPPPPPVVDPPPATHRAPPVPRIPPVSGVPPAPFAPFAPL
SRF1      236 PNFIKGGNLFN------VTIAPSPSPETPPSPTSPKRP-------FFGPP----SPNASA
SRF3      237 PKFLHEGNPFNATMINSTSTAPSLSPSLSPTKPAPTRP-------FSGVP----PPPNER
SRF6      235 T-LIKDGNSFN------TGPAPPPPPGTPPIR-GSPSR-------KSGGR----------
SRF7      233 N-LQKDGNLLN------SGPAPPPPPGTPPISKSSPTP-------KSGNR----------
SRF8      236 QTLIYDGNSFD------NVPASPQPE-----RPGKKET-------PSGSK----------
SRF4      235 GNLETGGNKWS------SGRAPSPPPG---------------------------------
SRF5      231 DSLLTGGNDWS------TETAPPPPPG---------------------------------
SRF2      233 PHLWIWGNKFH--------VEPNYKPWKFPLDVDRPLIQN------DTGYP----------
```

```
                                                                 |----
              sub-5           sub-15                          sub-2
              Q288*           P304L                           W337*
              |               |                                |
SUB/SRF9  288 QPQQHPPPSPPLVWSPPSSDNGGGDPWNSVSGQPTLQISPPSGSGSGKFWSTQRIILVVS
SRF1      279 GHGQAHVRSPPSDHHPSRPTPQGKE--DSFTSKRIIWISILG------------AFSFVV
SRF3      286 NRGK--VADGPSDSEGSSSENSKGK--NSSHTKKIILIAFAG------------VLVFII
SRF6      270 --------ESRSSDESTRNGDSKKS---GIGAGAIAGIIISL---------------LVV
SRF7      269 --------GNRSNGDSSNSKDSSKS---GLGAGGVAGIVISL---------------IVV
SRF8      268 --------KPKIGSEE-KSSDSGK----GLSGGVVTGIVFGS---------------LFV
SRF4      256 --------TRHIDRNSSGGGGGSSK---ALTLGVIIAVSSIG--------------GLIL
SRF5      252 --------VKYG-RKSSGSKDGGGI---TAGTGMVIAGACLG--------------VLVL
SRF2      269 -----TTESSAIMNFPRPETQKVKKKKKGIGAG-STFLLVGG---------------LAL
```

```
              --- TM --------|
                       sub-16
               V349M   G357S C365/6Y  K369E
               |       |       ||  |
SUB/SRF9  348 SVAIIVLVSGLCVTLWRCCRSKIYNRYYSGARKDLQRPYFNKPPSQPTPTMGKVSREPMV
SRF1      325 LALVCLLCGRKCLRKREDSEQLSKPHLTSEYGRAREGSRSNASMLPPSNTF---NKDKEA
SRF3      330 LVLAILLLLPKCARRREHANRVFKPHQVGADRGSRENALENGTPVLPPPGR---SEKVQR
SRF6      304 TALLVAFFLFRRKKSKRS----------SPMDIEKTDN---QPFTLASNDFH-ENNSIQS
SRF7      303 TA-VIAFFLIKRKRSKRS----------SSTDIEKTDNNINQPIILASNDFHQENKSVQN
SRF8      300 AG-IIALVLYLCLHKKKR----------KVRGSTRASQ---RSLPLSG-----------T
SRF4      291 FAGLIALISRRKNSNDSSHFFDDEK--GTNRSKPLFTPQSSQMLQFDNMEEFKNQKTVDS
SRF5      286 IIVLIALVSKKKSS-LSPHFIDEDNSHHTPKFKSLTSHGSAQELRVDFGNDYKDGKSGDS
SRF2      308 LGTFFALFAVRMNHRRAQN----------LAAIHRSNNSIAYSLPVSTGREYP-------
```

```
                                Q426R                           P456L A465G
                                |                               |        |
SUB/SRF9  408 KPFDGYGAGDRKYGYPMPQRAEE--SRRAMPPTSYYNKDVNTPQKPLQQPPRQFQSNDTA
SRF1      382 RPKERVGGASKLHGGAERSVGSE--SKQESHEIDMNGNAMDLMHPSSIPPIKRVIAKATE
SRF3      387 EPFKKAGEEPKVLHDLERLRRPAPISRQESQDIDFSMLMPPPPPPPPPPPPPPLDEKVTV
SRF6      350 SSSV------------------------ETKKLDTSLSINLRPPPIDRNKSFDDEDS-TR
SRF7      352 PPLV------------------------ETKKLDTSLSMNLRPPPSERHKSFDDDDSTMR
SRF8      335 PEVQ------------------------EQRVKSVASVADLKSSPAEKVT----VDRVMK
SRF4      349 NTSL-----------------------ETKPSVKRTSSVSFKNSPTFHLIPSTQVAATPD
SRF5      345 GD----------------------------ENIHRIGSKGLKHYVSSRVMSFTDTEFANK
SRF2      351 ---------------------------VATEDNPQIKRFQPPPAPQLRHLPSPPVRIDKS
```

```
                                                     |--  I
                 sub-17    sub-18                 T486E
                 S466L     P473L                  T486A      T494A
                 |         |                      |          |          *  *   *  **
SUB/SRF9  466  SKRAAHFPPGLNSSS------SATVFTIASLQQYTNNFSEENIIGEGSIGNVYRAELRHG
SRF1      440  PAEASLKRTTSKSHG---PLTAVKHFTVASLQQHTNSFSHENLIGTGMLGSVYRAELPGG
SRF3      447  MPIISPERPVKKTSPKRLPLTSVKHYSIASLQQYTESFAQENLIGSGMLGSVYRARLPNG
SRF6      385  KPIAVKKSTVVVPSN-------VRLYSVADLQIATGSFSVDNLLGEGTFGRVYRAEFDDG
SRF7      388  KPIVAKKAAVVVPSN-------VNTYTVSDLQVATNSFSVDNLLGEGTFGRVYRAQFEDG
SRF8      367  NGSISRIRSPITAS----------QYTVSSLQVATNSFSQENIIGEGSLGRVYRAEFPNG
SRF4      386  RSSTSQDSPDTRGVK---------AFSLADLQNTASCFSPNRLLGEGTIGRVYKAKFQDG
SRF5      377  LNAKRTTS--TRSAV---------EFELSDLQSATANFSPGNLLGEGSIGRVYRAKYSDG
SRF2      384  ARRKSFSATCQYPSF-------AKLFSAAELQLATNCFSEENLLGEGPLGSVYRAKLPDG

                        II             III               IV                  V
                                    sub-19
                   K525E          E539A  S545F
                 **|              |   *  |
SUB/SRF9  520  KFLAVKKLSNTINRTQSDGEFLNLVSNVLKLKRGHILELLGYCNEFGQRLLVYEYCPNGS
SRF1      497  KLFAVRKLDKKSPNHEEEGKFLELVNNIDRIRHANIVQLVGFCSEHSQRLLIHEYCRNGT
SRF3      507  KLFAVKKLDKRASEQQQDHEFIELVNNIDMIRHSNIVELVGYCAEHDQRLLVYEYCSNGT
SRF6      438  KVLAVKKIDSSALPHGMTDDFIEMVSKIANLDHPNVTKLVGYCAEHGQHLVVYEFHKNGS
SRF7      441  KVLAVKKIDSSALPTDTADDFTEIVSKIAHLDHENVTKLDGYCSEHGQHLVVYEFHRNGS
SRF8      417  KIMAIKKIDNAALSLQEEDNFLEAVSNMSRLRHPNIVPLAGYCTEHGQRLLVYEYVGNGN
SRF4      437  RKFAVKEIDSSLLGKGNPEEFSHIVSSISSIHHKNMAELVGYCSEQGRNMLVYEYFTSGS
SRF5      426  RTLAVKKIDSTLFDSGKSEGITPIVMSLSKIRHQNIAELVGYCSEQGHNMLVYEYFRNGS
SRF2      437  QFAVVRNIPMSSLSLHEEEQFTEVLQTASKLRHPNIVTLLGFCIENGEHLLVYEYVGHLS

                                      VIa                             VIb
                                     sub-4                               sub-20
                             T595N   R599C                               L633F
                                 *|*   *   *       *   ***          *  **  *   |
SUB/SRF9  580  LQDALHLDRKLHKKLTWNVRINIALGASKALQFLHEVCQPPVVHQNFKSSKVLLDGKLSV
SRF1      557  LHDLLHIDDRLKIELSWNVRVRIALEAAKALEYLHEICDPPSIHRNFKSANILLDDDIRV
SRF3      567  LQDGLHSDDEFKKKLSWNTRVSMALGAARALEYLHEVCEPPIIHRNFKSANVLLDDDLSV
SRF6      498  LHDFLHLSEEESKALVWNSRVKIALGTARALEYLHEVCSPSIVDKNIKSANILLDSELNP
SRF7      501  LHDFLHLAEEESKPLIWNPRVKIALGTARALEYLHEVCSPSIVHKNIKSANILLDSELNP
SRF8      477  LDDTLHTNDDRSMNLTWNARVKVALGTAKALEYLHEVCLPSIVHRNFKSANILLDEELNP
SRF4      497  LHRFLHLSDDFSKPLTWNTRIRIALGTAKAIEYLHETCSPPLVHKNIKSSNILLDNELNP
SRF5      486  LHEFLHLSDCFSKPLTWNTRVRIALGTARAVEYLHEACSPSVMHKNIKSSNILLDADLNP
SRF2      497  LYNAMH--DEVYKPLSWGLRLRIAIGVARALDYLHSSFCPPIAHSDLKATNILLDEELTP

                   |------ activation segment -----|
                   VII                 VIII                             IX
                                                      E667D
                                          sub-8/T-DNA |
                                  S656A              E665--*
                   *  *           |               ** ***  |
SUB/SRF9  640  RVADSGLAYMLPPRPTSQMAG----------YAAPEVEY-GSYTCQSDVFSLGVVMLELL
SRF1      617  HVSDCGLAPLISSGAVSQLSGQ---LLAAYGYGAPEFEY-GIYTMKCDVYSFGVVMLELL
SRF3      627  LVSDCGLAPLISSGSVSQLSGQ---LLAAYGYGAPEFDS-GIYTWQSDVYSFGVVMLELL
SRF6      558  HLSDSGLASFLPT-ANELLNQT---DE---GYSAPEVSMSGQYSLKSDIYSFGVVMLELL
SRF7      561  HLSDSGLASFLPT-ANELLNQN---DE---GYSAPETSMSGQYSLKSDVYSFGVVMLELL
SRF8      537  HLSDSGLAALTPN-TERQVSTQ---VVGSFGYSAPEFALSGIYTVKSDVYTFGVVMLELL
SRF4      557  RLSDYGLANFHHRTSQNLGVG----------YNAPECTDPSAYTQKSDVYSFGVVMLELL
SRF5      546  RLSDYGLSKFYLRTSQNLGEG----------YNAPEARDPSAYTPKSDVYSFGVVMLELL
SRF2      555  RIADCGLASLRPLTSNSVKLRASEIAIQNTGYIAPEHGQPGSSGTKSDTYALGVLLLELL
```

```
                                X

SUB/SRF9  689 TGRRPFDRTRPRGHQTLAQWAIPRLHDIDALTRMVDPSLHGAYPMKSLSRFADIISRSLQ
SRF1      673 TGRKSYDKKRDRGEQFLVRWAIPQLHDIDALAKMVDPSLKGDYPAKSLSHFADVISRCVQ
SRF3      683 TGRMSYDRDRSRGEQFLVRWAIPQLHDIDALGKMVDPSLNGQYPAKSLSHFADIISRCVQ
SRF6      611 TGRKPFDSTRSRSEQSLVRWATPQLHDIDALAKMVDPALKGLYPVKSLSRFADVIALCVQ
SRF7      614 TGRKPFDSTRSRSEQSLVRWATPQLHDIDALGKMVDPALKGLYPVKSLSRFADVIALCVQ
SRF8      593 TGRKPLDSSRTRAEQSLVRWATPQLHDIDALSKMVDPSLNGMYPAKSLSRFADIIALCIQ
SRF4      607 TGRKPYDSGRPKAEQSLVRWAKPQLKDMDTLDEMVDPALCGLYAPESVSSFADIVSICVM
SRF5      596 TGRVPFDGEKPRPERSLVRWATPQLHDIDALSNIADPALHGLYPPKSLSRFADIIALCVQ
SRF2      615 TGRKAFDSSRPRGEQLLVKWASTRLHDRRSLEQMIDGGIAGTFSSRVASQYADIISLCTQ

               XI          --|

                   *
SUB/SRF9  749 MEPGFRPPISEIVQDLQHMI----------------------------------------
SRF1      733 SEPEYRPLMSEVVQDLSDMIQREHRRN-DSNGDNQYTGRR--------------------
SRF3      743 SEPEFRPLMSEVVQDLLDMIRRERHGSGDSTAD---------------------------
SRF6      671 PEPEFRPPMSEVVQALVVLVQRANMSKRTVGVDPSQRAGSADTTSDYM------------
SRF7      674 PEPEFRPPMSEVVQALVVLVQRANMSKRTVGVG----SGSSGVN-DYM------------
SRF8      653 PEPEFRPPMSEVVQQLVRLVQRASVVKRRSSDD----TGFSYRTPEHEHVDISF------
SRF4      667 TEPGLRPPVSNVVEALKRLV----------------------------------------
SRF5      656 VEPEFRPPMSEVVEALVRMVQRSSMKLKDDLSSSYRAHDDYDY-----------------
SRF2      675 AEKEFRPPVSEIVEALTALIQKQNKEASSSVADKTDPFSKSFCSTRTRFISSPTFSYLSS
```
