# Supplementary material for: Structure-Function Analysis of STRUBBELIG, an Arabidopsis Atypical Receptor-Like Kinase Involved in Tissue Morphogenesis
Source: PLoS One. 2011 May 16;6(5):e19730. doi: 10.1371/journal.pone.0019730 (PMC3095605; doi:10.1371/journal.pone.0019730)
Supplement: Figure S2 — SUB protein sequence alignment from 57 different Arabidopsis accessions. ClustalW alignment. Depicts some of the natural variation in SUB. At1g11130.1_REF corresponds to the TAIR10 reference sequence of SUB. (PDF) [file pone.0019730.s002.pdf]

CLUSTAL 2.1 multiple sequence alignment

```
AT1G11130.1_Acc_Del-10          MSFTRWEVFFGLSVLALTMPFSAGVTNLRDVSAINNLYITLGAPSLHHWL 50
AT1G11130.1_Acc_ICE107          MSFTRWEVFFGLSVLALTMPFSAGVTNLRDVSAINNLYITLGAPSLHHWL 50
AT1G11130.1_Acc_Nie1-2          MSFTRWEVFFGLSVLALTMPFSAGVTNLRDVSAINNLYITLGAPSLHHWL 50
AT1G11130.1_Acc_Ped-0           MSFTRWEVFFGLSVLALTMPFSAGVTNLRDVSAINNLYITLGAPSLHHWL 50
AT1G11130.1_Acc_Koch-1          MSFTRWEVFFGLSVLALTMPFSAGVTNLRDVSAINNLYITLGAPSLHHWL 50
AT1G11130.1_Acc_TueSB30-3       MSFTRWEVFFGLSVLALTMPFSAGVTNLRDVSAINNLYITLGAPSLHHWL 50
AT1G11130.1_Acc_Yeg-1           MSFTRWEVFFGLSVLALTMPFSAGVTNLRDVSAINNLYITLGAPSLHHWL 50
AT1G11130.1_Acc_ICE216          MSFTRWEVFFGLSVLALTMPFSAGVTNLRDVSAINNLYITLGAPSLHHWL 50
AT1G11130.1_Acc_ICE72           MSFTRWEVFFGLSVLALTMPFSAGVTNLRDVSAINNLYITLGAPSLHHWL 50
AT1G11130.1_Acc_ICE50           MSFTRWEVFFGLSVLALTMPFSAGVTNLRDVSAINNLYITLGAPSLHHWL 50
AT1G11130.1_Acc_ICE60           MSFTRWEVFFGLSVLALTMPFSAGVTNLRDVSAINNLYITLGAPSLHHWL 50
AT1G11130.1_Acc_Mer-6           MSFTRWEVFFGLSVLALTMPFSAGVTNLRDVSAINNLYITLGAPSLHHWL 50
AT1G11130.1_Acc_Nemrut-1        MSFTRWEVFFGLSVLALTMPFSAGVTNLRDVSAINNLYITLGAPSLHHWL 50
AT1G11130.1_Acc_Sha             MSFTRWEVFFGLSVLALTMPFSAGVTNLRDVSAINNLYITLGAPSLHHWL 50
AT1G11130.1_Acc_Rue3-1-31       MSFTRWEVFFGLSVLALTMPFSAGVTNLRDVSAINNLYITLGAPSLHHWL 50
AT1G11130.1_Acc_Leo-1           MSFTRWEVFFGLSVLALTMPFSAGVTNLRDVSAINNLYITLGAPSLHHWL 50
AT1G11130.1_Acc_Istisu-1        MSFTRWEVFFGLSVLALTMPFSAGVTNLRDVSAINNLYITLGAPSLHHWL 50
AT1G11130.1_Acc_ICE98           MSFTRWEVFFGLSVLALTMPFSAGVTNLRDVSAINNLYITLGAPSLHHWL 50
AT1G11130.1_Acc_ICE97           MSFTRWEVFFGLSVLALTMPFSAGVTNLRDVSAINNLYITLGAPSLHHWL 50
AT1G11130.1_Acc_ICE92           MSFTRWEVFFGLSVLALTMPFSAGVTNLRDVSAINNLYITLGAPSLHHWL 50
AT1G11130.1_Acc_ICE79           MSFTRWEVFFGLSVLALTMPFSAGVTNLRDVSAINNLYITLGAPSLHHWL 50
AT1G11130.1_Acc_ICE75           MSFTRWEVFFGLSVLALTMPFSAGVTNLRDVSAINNLYITLGAPSLHHWL 50
AT1G11130.1_Acc_ICE71           MSFTRWEVFFGLSVLALTMPFSAGVTNLRDVSAINNLYITLGAPSLHHWL 50
AT1G11130.1_Acc_ICE7            MSFTRWEVFFGLSVLALTMPFSAGVTNLRDVSAINNLYITLGAPSLHHWL 50
AT1G11130.1_Acc_ICE49           MSFTRWEVFFGLSVLALTMPFSAGVTNLRDVSAINNLYITLGAPSLHHWL 50
AT1G11130.1_Acc_ICE36           MSFTRWEVFFGLSVLALTMPFSAGVTNLRDVSAINNLYITLGAPSLHHWL 50
AT1G11130.1_Acc_ICE33           MSFTRWEVFFGLSVLALTMPFSAGVTNLRDVSAINNLYITLGAPSLHHWL 50
AT1G11130.1_Acc_ICE228          MSFTRWEVFFGLSVLALTMPFSAGVTNLRDVSAINNLYITLGAPSLHHWL 50
AT1G11130.1_Acc_ICE213          MSFTRWEVFFGLSVLALTMPFSAGVTNLRDVSAINNLYITLGAPSLHHWL 50
AT1G11130.1_Acc_ICE212          MSFTRWEVFFGLSVLALTMPFSAGVTNLRDVSAINNLYITLGAPSLHHWL 50
AT1G11130.1_Acc_ICE21           MSFTRWEVFFGLSVLALTMPFSAGVTNLRDVSAINNLYITLGAPSLHHWL 50
AT1G11130.1_Acc_ICE173          MSFTRWEVFFGLSVLALTMPFSAGVTNLRDVSAINNLYITLGAPSLHHWL 50
AT1G11130.1_Acc_ICE169          MSFTRWEVFFGLSVLALTMPFSAGVTNLRDVSAINNLYITLGAPSLHHWL 50
AT1G11130.1_Acc_ICE153          MSFTRWEVFFGLSVLALTMPFSAGVTNLRDVSAINNLYITLGAPSLHHWL 50
AT1G11130.1_Acc_ICE150          MSFTRWEVFFGLSVLALTMPFSAGVTNLRDVSAINNLYITLGAPSLHHWL 50
AT1G11130.1_Acc_ICE138          MSFTRWEVFFGLSVLALTMPFSAGVTNLRDVSAINNLYITLGAPSLHHWL 50
AT1G11130.1_Acc_ICE134          MSFTRWEVFFGLSVLALTMPFSAGVTNLRDVSAINNLYITLGAPSLHHWL 50
AT1G11130.1_Acc_ICE130          MSFTRWEVFFGLSVLALTMPFSAGVTNLRDVSAINNLYITLGAPSLHHWL 50
AT1G11130.1_Acc_ICE119          MSFTRWEVFFGLSVLALTMPFSAGVTNLRDVSAINNLYITLGAPSLHHWL 50
AT1G11130.1_Acc_ICE111          MSFTRWEVFFGLSVLALTMPFSAGVTNLRDVSAINNLYITLGAPSLHHWL 50
AT1G11130.1_Acc_ICE106          MSFTRWEVFFGLSVLALTMPFSAGVTNLRDVSAINNLYITLGAPSLHHWL 50
AT1G11130.1_Acc_ICE104          MSFTRWEVFFGLSVLALTMPFSAGVTNLRDVSAINNLYITLGAPSLHHWL 50
AT1G11130.1_Acc_ICE1            MSFTRWEVFFGLSVLALTMPFSAGVTNLRDVSAINNLYITLGAPSLHHWL 50
AT1G11130.1_Acc_Fei-0           MSFTRWEVFFGLSVLALTMPFSAGVTNLRDVSAINNLYITLGAPSLHHWL 50
AT1G11130.1_Acc_Dog-4           MSFTRWEVFFGLSVLALTMPFSAGVTNLRDVSAINNLYITLGAPSLHHWL 50
AT1G11130.1_Acc_Cdm-0           MSFTRWEVFFGLSVLALTMPFSAGVTNLRDVSAINNLYITLGAPSLHHWL 50
AT1G11130.1_Acc_Bak-2           MSFTRWEVFFGLSVLALTMPFSAGVTNLRDVSAINNLYITLGAPSLHHWL 50
AT1G11130.1_Acc_Agu-1           MSFTRWEVFFGLSVLALTMPFSAGVTNLRDVSAINNLYITLGAPSLHHWL 50
AT1G11130.1_REF                 MSFTRWEVFFGLSVLALTMPFSAGVTNLRDVSAINNLYITLGAPSLHHWL 50
AT1G11130.1_Acc_ICE120          MSFTRWEVFFGLSVLALTMPFSAGVTNLRDVSAINNLYITLGAPSLHHWL 50
AT1G11130.1_Acc_Tuescha-9       MSFTRWEVFFGLSVLALTMPFSAGVTNLRDVSAINNLYITLGAPSLHHWL 50
AT1G11130.1_Acc_TueWa1-2        MSFTRWEVFFGLSVLALTMPFSAGVTNLRDVSAINNLYITLGAPSLHHWL 50
AT1G11130.1_Acc_Vash-1          MSFTRWEVFFGLSVLALTMPFSAGVTNLRDVSAINNLYITLGAPSLHHWL 50
AT1G11130.1_Acc_Pra-6           MSFTRWEVFFGLSVLALTMPFSAGVTNLRDVSAINNLYITLGAPSLHHWL 50
AT1G11130.1_Acc_ICE102          MTFTRWEVLFVLSVLALTMPFTAGVTNLRDVSAINNLYITLGAPSLHHWL 50
AT1G11130.1_Acc_Qui-0           MTFTRWEVLFVLSVLALTMPFTAGVTNLRDVSAINNLYITLGAPSLHHWL 50
AT1G11130.1_Acc_ICE61           MTFTRWEVFFVLSVLALTMPFTAGVTNLRDVSAINNLYITLGAPSLHHWL 50
                                *:******:* **********:****************************

AT1G11130.1_Acc_Del-10          AFGGDPCGEKWQGVVCDSSNITEIRIPGMKVGGGLSDTLADFSSIQVMDF 100
AT1G11130.1_Acc_ICE107          AFGGDPCGEKWQGVVCDSSNITEIRIPGMKVGGGLSDTLADFSSIQVMDF 100
AT1G11130.1_Acc_Nie1-2          AFGGDPCGEKWQGVVCDSSNITEIRIPGMKVGGGLSDTLADFSSIQVMDF 100
AT1G11130.1_Acc_Ped-0           AFGGDPCGEKWQGVVCDSSNITEIRIPGMKVGGGLSDTLADFSSIQVMDF 100
AT1G11130.1_Acc_Koch-1          AFGGDPCGEKWQGVVCDSSNITEIRIPGMKVGGGLSDTLADFSSIQVMDF 100
AT1G11130.1_Acc_TueSB30-3       AFGGDPCGEKWQGVVCDSSNITEIRIPGMKVGGGLSDTLADFSSIQVMDF 100
AT1G11130.1_Acc_Yeg-1           AFGGDPCGEKWQGVVCDSSNITEIRIPGMKVGGGLSDTLADFSSIQVMDF 100
AT1G11130.1_Acc_ICE216          AFGGDPCGEKWQGVVCDSSNITEIRIPGMKVGGGLSDTLADFSSIQVMDF 100
AT1G11130.1_Acc_ICE72           AFGGDPCGEKWQGVVCDSSNITEIRIPGMKVGGGLSDTLADFSSIQVMDF 100
AT1G11130.1_Acc_ICE50           AFGGDPCGEKWQGVVCDSSNITEIRIPGMKVGGGLSDTLADFSSIQVMDF 100
AT1G11130.1_Acc_ICE60           AFGGDPCGEKWQGVVCDSSNITEIRIPGMKVGGGLSDTLADFSSIQVMDF 100
AT1G11130.1_Acc_Mer-6           AFGGDPCGEKWQGVVCDSSNITEIRIPGMKVGGGLSDTLADFSSIQVMDF 100
AT1G11130.1_Acc_Nemrut-1        AFGGDPCGEKWQGVVCDSSNITEIRIPGMKVGGGLSDTLADFSSIQVMDF 100
AT1G11130.1_Acc_Sha             AFGGDPCGEKWQGVVCDSSNITEIRIPGMKVGGGLSDTLADFSSIQVMDF 100
AT1G11130.1_Acc_Rue3-1-31       AFGGDPCGEKWQGVVCDSSNITEIRIPGMKVGGGLSDTLADFSSIQVMDF 100
AT1G11130.1_Acc_Leo-1           AFGGDPCGEKWQGVVCDSSNITEIRIPGMKVGGGLSDTLADFSSIQVMDF 100
AT1G11130.1_Acc_Istisu-1        AFGGDPCGEKWQGVVCDSSNITEIRIPGMKVGGGLSDTLADFSSIQVMDF 100
AT1G11130.1_Acc_ICE98           AFGGDPCGEKWQGVVCDSSNITEIRIPGMKVGGGLSDTLADFSSIQVMDF 100
AT1G11130.1_Acc_ICE97           AFGGDPCGEKWQGVVCDSSNITEIRIPGMKVGGGLSDTLADFSSIQVMDF 100
AT1G11130.1_Acc_ICE92           AFGGDPCGEKWQGVVCDSSNITEIRIPGMKVGGGLSDTLADFSSIQVMDF 100
AT1G11130.1_Acc_ICE79           AFGGDPCGEKWQGVVCDSSNITEIRIPGMKVGGGLSDTLADFSSIQVMDF 100
AT1G11130.1_Acc_ICE75           AFGGDPCGEKWQGVVCDSSNITEIRIPGMKVGGGLSDTLADFSSIQVMDF 100
AT1G11130.1_Acc_ICE71           AFGGDPCGEKWQGVVCDSSNITEIRIPGMKVGGGLSDTLADFSSIQVMDF 100
AT1G11130.1_Acc_ICE7            AFGGDPCGEKWQGVVCDSSNITEIRIPGMKVGGGLSDTLADFSSIQVMDF 100
AT1G11130.1_Acc_ICE49           AFGGDPCGEKWQGVVCDSSNITEIRIPGMKVGGGLSDTLADFSSIQVMDF 100
AT1G11130.1_Acc_ICE36           AFGGDPCGEKWQGVVCDSSNITEIRIPGMKVGGGLSDTLADFSSIQVMDF 100
AT1G11130.1_Acc_ICE33           AFGGDPCGEKWQGVVCDSSNITEIRIPGMKVGGGLSDTLADFSSIQVMDF 100
AT1G11130.1_Acc_ICE228          AFGGDPCGEKWQGVVCDSSNITEIRIPGMKVGGGLSDTLADFSSIQVMDF 100
AT1G11130.1_Acc_ICE213          AFGGDPCGEKWQGVVCDSSNITEIRIPGMKVGGGLSDTLADFSSIQVMDF 100
AT1G11130.1_Acc_ICE212          AFGGDPCGEKWQGVVCDSSNITEIRIPGMKVGGGLSDTLADFSSIQVMDF 100
AT1G11130.1_Acc_ICE21           AFGGDPCGEKWQGVVCDSSNITEIRIPGMKVGGGLSDTLADFSSIQVMDF 100
AT1G11130.1_Acc_ICE173          AFGGDPCGEKWQGVVCDSSNITEIRIPGMKVGGGLSDTLADFSSIQVMDF 100
```

```
AT1G11130.1_Acc_ICE169        AFGGDPCGEKWQGVVCDSSNITEIRIPGMKVGGGLSDTLADFSSIQVMDF 100
AT1G11130.1_Acc_ICE153        AFGGDPCGEKWQGVVCDSSNITEIRIPGMKVGGGLSDTLADFSSIQVMDF 100
AT1G11130.1_Acc_ICE150        AFGGDPCGEKWQGVVCDSSNITEIRIPGMKVGGGLSDTLADFSSIQVMDF 100
AT1G11130.1_Acc_ICE138        AFGGDPCGEKWQGVVCDSSNITEIRIPGMKVGGGLSDTLADFSSIQVMDF 100
AT1G11130.1_Acc_ICE134        AFGGDPCGEKWQGVVCDSSNITEIRIPGMKVGGGLSDTLADFSSIQVMDF 100
AT1G11130.1_Acc_ICE130        AFGGDPCGEKWQGVVCDSSNITEIRIPGMKVGGGLSDTLADFSSIQVMDF 100
AT1G11130.1_Acc_ICE119        AFGGDPCGEKWQGVVCDSSNITEIRIPGMKVGGGLSDTLADFSSIQVMDF 100
AT1G11130.1_Acc_ICE111        AFGGDPCGEKWQGVVCDSSNITEIRIPGMKVGGGLSDTLADFSSIQVMDF 100
AT1G11130.1_Acc_ICE106        AFGGDPCGEKWQGVVCDSSNITEIRIPGMKVGGGLSDTLADFSSIQVMDF 100
AT1G11130.1_Acc_ICE104        AFGGDPCGEKWQGVVCDSSNITEIRIPGMKVGGGLSDTLADFSSIQVMDF 100
AT1G11130.1_Acc_ICE1          AFGGDPCGEKWQGVVCDSSNITEIRIPGMKVGGGLSDTLADFSSIQVMDF 100
AT1G11130.1_Acc_Fei-0         AFGGDPCGEKWQGVVCDSSNITEIRIPGMKVGGGLSDTLADFSSIQVMDF 100
AT1G11130.1_Acc_Dog-4         AFGGDPCGEKWQGVVCDSSNITEIRIPGMKVGGGLSDTLADFSSIQVMDF 100
AT1G11130.1_Acc_Cdm-0         AFGGDPCGEKWQGVVCDSSNITEIRIPGMKVGGGLSDTLADFSSIQVMDF 100
AT1G11130.1_Acc_Bak-2         AFGGDPCGEKWQGVVCDSSNITEIRIPGMKVGGGLSDTLADFSSIQVMDF 100
AT1G11130.1_Acc_Agu-1         AFGGDPCGEKWQGVVCDSSNITEIRIPGMKVGGGLSDTLADFSSIQVMDF 100
AT1G11130.1_REF               AFGGDPCGEKWQGVVCDSSNITEIRIPGMKVGGGLSDTLADFSSIQVMDF 100
AT1G11130.1_Acc_ICE120        AFGGDPCGEKWQGVVCDSSNITEIRIPGMKVGGGLSDTLADFSSIQVMDF 100
AT1G11130.1_Acc_Tuescha-9     AFGGDPCGEKWQGVVCDSSNITEIRIPGMKVGGGLSDTLADFSSIQVMDF 100
AT1G11130.1_Acc_TueWa1-2      AFGGDPCGEKWQGVVCDSSNITEIRIPGMKVGGGLSDTLADFSSIQVMDF 100
AT1G11130.1_Acc_Vash-1        AFGGDPCGEKWQGVVCDSSNITEIRIPGMKVGGGLSDTLADFSSIQVMDF 100
AT1G11130.1_Acc_Pra-6         AFGGDPCGEKWQGVVCDSSNITEIRIPGMKVGGGLSDTLADFSSIQVMDF 100
AT1G11130.1_Acc_ICE102        AFGGDPCGEKWQGVVCDSSNITEIRIPGMKVGGGLSDTLADFSSIQVMDF 100
AT1G11130.1_Acc_Qui-0         AFGGDPCGEKWQGVVCDSSNITEIRIPGMKVGGGLSDTLADFSSIQVMDF 100
AT1G11130.1_Acc_ICE61         AFGGDPCGEKWQGVVCDSSNITEIRIPGMKVGGGLSDTLADFSSIQVMDF 100
                              **************************************************

AT1G11130.1_Acc_Del-10        SSNHISGTIPQALPSSIRNLSLSSNRFTGNIPFTLSFLSDLSELSLGSNL 150
AT1G11130.1_Acc_ICE107        SSNHISGTIPQALPSSIRNLSLSSNRFTGNIPFTLSFLSDLSELSLGSNL 150
AT1G11130.1_Acc_Nie1-2        SSNHISGTIPQALPSSIRNLSLSSNRFTGNIPFTLSFLSDLSELSLGSNL 150
AT1G11130.1_Acc_Ped-0         SSNHISGTIPQALPSSIRNLSLSSNRFTGNIPFTLSFLSDLSELSLGSNL 150
AT1G11130.1_Acc_Koch-1        SSNHISGTIPQALPSSIRNLSLSSNRFTGNIPFTLSFLSDLSELSLGSNL 150
AT1G11130.1_Acc_TueSB30-3     SSNHISGTIPQALPSSIRNLSLSSNRFTGNIPFTLSFLSDLSELSLGSNL 150
AT1G11130.1_Acc_Yeg-1         SSNHISGTIPQALPSSIRNLSLSSNRFTGNIPFTLSFLSDLSELSLGSNL 150
AT1G11130.1_Acc_ICE216        SSNHISGTIPQALPSSIRNLSLSSNRFTGNIPFTLSFLSDLSELSLGSNL 150
AT1G11130.1_Acc_ICE72         SSNHISGTIPQALPSSIRNLSLSSNRFTGNIPFTLSFLSDLSELSLGSNL 150
AT1G11130.1_Acc_ICE50         SSNHISGTIPQALPSSIRNLSLSSNRFTGNIPFTLSFLSDLSELSLGSNL 150
AT1G11130.1_Acc_ICE60         SSNHISGTIPQALPSSIRNLSLSSNRFTGNIPFTLSFLSDLSELSLGSNL 150
AT1G11130.1_Acc_Mer-6         SSNHISGTIPQALPSSIRNLSLSSNRFTGNIPFTLSFLSDLSELSLGSNL 150
AT1G11130.1_Acc_Nemrut-1      SSNHISGTIPQALPSSIRNLSLSSNRFTGNIPFTLSFLSDLSELSLGSNL 150
AT1G11130.1_Acc_Sha           SSNHISGTIPQALPSSIRNLSLSSNRFTGNIPFTLSFLSDLSELSLGSNL 150
AT1G11130.1_Acc_Rue3-1-31     SSNHISGTIPQALPSSIRNLSLSSNRFTGNIPFTLSFLSDLSELSLGSNL 150
AT1G11130.1_Acc_Leo-1         SSNHISGTIPQALPSSIRNLSLSSNRFTGNIPFTLSFLSDLSELSLGSNL 150
AT1G11130.1_Acc_Istisu-1      SSNHISGTIPQALPSSIRNLSLSSNRFTGNIPFTLSFLSDLSELSLGSNL 150
AT1G11130.1_Acc_ICE98         SSNHISGTIPQALPSSIRNLSLSSNRFTGNIPFTLSFLSDLSELSLGSNL 150
AT1G11130.1_Acc_ICE97         SSNHISGTIPQALPSSIRNLSLSSNRFTGNIPFTLSFLSDLSELSLGSNL 150
AT1G11130.1_Acc_ICE92         SSNHISGTIPQALPSSIRNLSLSSNRFTGNIPFTLSFLSDLSELSLGSNL 150
AT1G11130.1_Acc_ICE79         SSNHISGTIPQALPSSIRNLSLSSNRFTGNIPFTLSFLSDLSELSLGSNL 150
AT1G11130.1_Acc_ICE75         SSNHISGTIPQALPSSIRNLSLSSNRFTGNIPFTLSFLSDLSELSLGSNL 150
AT1G11130.1_Acc_ICE71         SSNHISGTIPQALPSSIRNLSLSSNRFTGNIPFTLSFLSDLSELSLGSNL 150
AT1G11130.1_Acc_ICE7          SSNHISGTIPQALPSSIRNLSLSSNRFTGNIPFTLSFLSDLSELSLGSNL 150
AT1G11130.1_Acc_ICE49         SSNHISGTIPQALPSSIRNLSLSSNRFTGNIPFTLSFLSDLSELSLGSNL 150
AT1G11130.1_Acc_ICE36         SSNHISGTIPQALPSSIRNLSLSSNRFTGNIPFTLSFLSDLSELSLGSNL 150
AT1G11130.1_Acc_ICE33         SSNHISGTIPQALPSSIRNLSLSSNRFTGNIPFTLSFLSDLSELSLGSNL 150
AT1G11130.1_Acc_ICE228        SSNHISGTIPQALPSSIRNLSLSSNRFTGNIPFTLSFLSDLSELSLGSNL 150
AT1G11130.1_Acc_ICE213        SSNHISGTIPQALPSSIRNLSLSSNRFTGNIPFTLSFLSDLSELSLGSNL 150
AT1G11130.1_Acc_ICE212        SSNHISGTIPQALPSSIRNLSLSSNRFTGNIPFTLSFLSDLSELSLGSNL 150
AT1G11130.1_Acc_ICE21         SSNHISGTIPQALPSSIRNLSLSSNRFTGNIPFTLSFLSDLSELSLGSNL 150
AT1G11130.1_Acc_ICE173        SSNHISGTIPQALPSSIRNLSLSSNRFTGNIPFTLSFLSDLSELSLGSNL 150
AT1G11130.1_Acc_ICE169        SSNHISGTIPQALPSSIRNLSLSSNRFTGNIPFTLSFLSDLSELSLGSNL 150
AT1G11130.1_Acc_ICE153        SSNHISGTIPQALPSSIRNLSLSSNRFTGNIPFTLSFLSDLSELSLGSNL 150
AT1G11130.1_Acc_ICE150        SSNHISGTIPQALPSSIRNLSLSSNRFTGNIPFTLSFLSDLSELSLGSNL 150
AT1G11130.1_Acc_ICE138        SSNHISGTIPQALPSSIRNLSLSSNRFTGNIPFTLSFLSDLSELSLGSNL 150
AT1G11130.1_Acc_ICE134        SSNHISGTIPQALPSSIRNLSLSSNRFTGNIPFTLSFLSDLSELSLGSNL 150
AT1G11130.1_Acc_ICE130        SSNHISGTIPQALPSSIRNLSLSSNRFTGNIPFTLSFLSDLSELSLGSNL 150
AT1G11130.1_Acc_ICE119        SSNHISGTIPQALPSSIRNLSLSSNRFTGNIPFTLSFLSDLSELSLGSNL 150
AT1G11130.1_Acc_ICE111        SSNHISGTIPQALPSSIRNLSLSSNRFTGNIPFTLSFLSDLSELSLGSNL 150
AT1G11130.1_Acc_ICE106        SSNHISGTIPQALPSSIRNLSLSSNRFTGNIPFTLSFLSDLSELSLGSNL 150
AT1G11130.1_Acc_ICE104        SSNHISGTIPQALPSSIRNLSLSSNRFTGNIPFTLSFLSDLSELSLGSNL 150
AT1G11130.1_Acc_ICE1          SSNHISGTIPQALPSSIRNLSLSSNRFTGNIPFTLSFLSDLSELSLGSNL 150
AT1G11130.1_Acc_Fei-0         SSNHISGTIPQALPSSIRNLSLSSNRFTGNIPFTLSFLSDLSELSLGSNL 150
AT1G11130.1_Acc_Dog-4         SSNHISGTIPQALPSSIRNLSLSSNRFTGNIPFTLSFLSDLSELSLGSNL 150
AT1G11130.1_Acc_Cdm-0         SSNHISGTIPQALPSSIRNLSLSSNRFTGNIPFTLSFLSDLSELSLGSNL 150
AT1G11130.1_Acc_Bak-2         SSNHISGTIPQALPSSIRNLSLSSNRFTGNIPFTLSFLSDLSELSLGSNL 150
AT1G11130.1_Acc_Agu-1         SSNHISGTIPQALPSSIRNLSLSSNRFTGNIPFTLSFLSDLSELSLGSNL 150
AT1G11130.1_REF               SSNHISGTIPQALPSSIRNLSLSSNRFTGNIPFTLSFLSDLSELSLGSNL 150
AT1G11130.1_Acc_ICE120        SSNHISGTIPQALPSSIRNLSLSSNRFTGNIPFTLSFLSDLSELSLGSNL 150
AT1G11130.1_Acc_Tuescha-9     SSNHISGTIPQALPSSIRNLSLSSNRFTGNIPFTLSFLSDLSELSLGSNL 150
AT1G11130.1_Acc_TueWa1-2      SSNHISGTIPQALPSSIRNLSLSSNRFTGNIPFTLSFLSDLSELSLGSNL 150
AT1G11130.1_Acc_Vash-1        SSNHISGTIPQALPSSIRNLSLSSNRFTGNIPFTLSFLSDLSELSLGSNL 150
AT1G11130.1_Acc_Pra-6         SSNHISGTIPQALPSSIRNLSLSSNRFTGNIPFTLSFLSDLSELSLGSNL 150
AT1G11130.1_Acc_ICE102        SSNHISGTIPQALPSSIRNLSLSSNRFTGNIPFTLSFLSDLSELSLGSNL 150
AT1G11130.1_Acc_Qui-0         SSNHISGTIPQALPSSIRNLSLSSNRFTGNIPFTLSFLSDLSELSLGSNL 150
AT1G11130.1_Acc_ICE61         SSNHISGTIPQALPSSIRNLSLSSNRFTGNIPFTLSFLSDLSELSLGSNL 150
                              **************************************************

AT1G11130.1_Acc_Del-10        LSGEIPDYFQQLSKLTKLDLSSNILEGHLPSSMGDLASLKILYLQDNKLT 200
AT1G11130.1_Acc_ICE107        LSGEIPDYFQQLSKLTKLDLSSNILEGHLPSSMGDLASLKILYLQDNKLT 200
AT1G11130.1_Acc_Nie1-2        LSGEIPDYFQQLSKLTKLDLSSNILEGHLPSSMGDLASLKILYLQDNKLT 200
AT1G11130.1_Acc_Ped-0         LSGEIPDYFQQLSKLTKLDLSSNILEGHLPSSMGDLASLKILYLQDNKLT 200
AT1G11130.1_Acc_Koch-1        LSGEIPDYFQQLSKLTKLDLSSNILEGHLPSSMGDLASLKILYLQDNKLT 200
AT1G11130.1_Acc_TueSB30-3     LSGEIPDYFQQLSKLTKLDLSSNILEGHLPSSMGDLASLKILYLQDNKLT 200
AT1G11130.1_Acc_Yeg-1         LSGEIPDYFQQLSKLTKLDLSSNILEGHLPSSMGDLASLKILYLQDNKLT 200
AT1G11130.1_Acc_ICE216        LSGEIPDYFQQLSKLTKLDLSSNILEGHLPSSMGDLASLKILYLQDNKLT 200
```

```
AT1G11130.1_Acc_ICE72          LSGEIPDYFQQLSKLTKLDLSSNILEGHLPSSMGDLASLKILYLQDNKLT 200
AT1G11130.1_Acc_ICE50          LSGEIPDYFQQLSKLTKLDLSSNILEGHLPSSMGDLASLKILYLQDNKLT 200
AT1G11130.1_Acc_ICE60          LSGEIPDYFQQLSKLTKLDLSSNILEGHLPSSMGDLASLKILYLQDNKLT 200
AT1G11130.1_Acc_Mer-6          LSGEIPDYFQQLSKLTKLDLSSNILEGHLPSSMGDLASLKILYLQDNKLT 200
AT1G11130.1_Acc_Nemrut-1       LSGEIPDYFQQLSKLTKLDLSSNILEGHLPSSMGDLASLKILYLQDNKLT 200
AT1G11130.1_Acc_Sha            LSGEIPDYFQQLSKLTKLDLSSNILEGHLPSSMGDLASLKILYLQDNKLT 200
AT1G11130.1_Acc_Rue3-1-31      LSGEIPDYFQQLSKLTKLDLSSNILEGHLPSSMGDLASLKILYLQDNKLT 200
AT1G11130.1_Acc_Leo-1          LSGEIPDYFQQLSKLTKLDLSSNILEGHLPSSMGDLASLKILYLQDNKLT 200
AT1G11130.1_Acc_Istisu-1       LSGEIPDYFQQLSKLTKLDLSSNILEGHLPSSMGDLASLKILYLQDNKLT 200
AT1G11130.1_Acc_ICE98          LSGEIPDYFQQLSKLTKLDLSSNILEGHLPSSMGDLASLKILYLQDNKLT 200
AT1G11130.1_Acc_ICE97          LSGEIPDYFQQLSKLTKLDLSSNILEGHLPSSMGDLASLKILYLQDNKLT 200
AT1G11130.1_Acc_ICE92          LSGEIPDYFQQLSKLTKLDLSSNILEGHLPSSMGDLASLKILYLQDNKLT 200
AT1G11130.1_Acc_ICE79          LSGEIPDYFQQLSKLTKLDLSSNILEGHLPSSMGDLASLKILYLQDNKLT 200
AT1G11130.1_Acc_ICE75          LSGEIPDYFQQLSKLTKLDLSSNILEGHLPSSMGDLASLKILYLQDNKLT 200
AT1G11130.1_Acc_ICE71          LSGEIPDYFQQLSKLTKLDLSSNILEGHLPSSMGDLASLKILYLQDNKLT 200
AT1G11130.1_Acc_ICE7           LSGEIPDYFQQLSKLTKLDLSSNILEGHLPSSMGDLASLKILYLQDNKLT 200
AT1G11130.1_Acc_ICE49          LSGEIPDYFQQLSKLTKLDLSSNILEGHLPSSMGDLASLKILYLQDNKLT 200
AT1G11130.1_Acc_ICE36          LSGEIPDYFQQLSKLTKLDLSSNILEGHLPSSMGDLASLKILYLQDNKLT 200
AT1G11130.1_Acc_ICE33          LSGEIPDYFQQLSKLTKLDLSSNILEGHLPSSMGDLASLKILYLQDNKLT 200
AT1G11130.1_Acc_ICE228         LSGEIPDYFQQLSKLTKLDLSSNILEGHLPSSMGDLASLKILYLQDNKLT 200
AT1G11130.1_Acc_ICE213         LSGEIPDYFQQLSKLTKLDLSSNILEGHLPSSMGDLASLKILYLQDNKLT 200
AT1G11130.1_Acc_ICE212         LSGEIPDYFQQLSKLTKLDLSSNILEGHLPSSMGDLASLKILYLQDNKLT 200
AT1G11130.1_Acc_ICE21          LSGEIPDYFQQLSKLTKLDLSSNILEGHLPSSMGDLASLKILYLQDNKLT 200
AT1G11130.1_Acc_ICE173         LSGEIPDYFQQLSKLTKLDLSSNILEGHLPSSMGDLASLKILYLQDNKLT 200
AT1G11130.1_Acc_ICE169         LSGEIPDYFQQLSKLTKLDLSSNILEGHLPSSMGDLASLKILYLQDNKLT 200
AT1G11130.1_Acc_ICE153         LSGEIPDYFQQLSKLTKLDLSSNILEGHLPSSMGDLASLKILYLQDNKLT 200
AT1G11130.1_Acc_ICE150         LSGEIPDYFQQLSKLTKLDLSSNILEGHLPSSMGDLASLKILYLQDNKLT 200
AT1G11130.1_Acc_ICE138         LSGEIPDYFQQLSKLTKLDLSSNILEGHLPSSMGDLASLKILYLQDNKLT 200
AT1G11130.1_Acc_ICE134         LSGEIPDYFQQLSKLTKLDLSSNILEGHLPSSMGDLASLKILYLQDNKLT 200
AT1G11130.1_Acc_ICE130         LSGEIPDYFQQLSKLTKLDLSSNILEGHLPSSMGDLASLKILYLQDNKLT 200
AT1G11130.1_Acc_ICE119         LSGEIPDYFQQLSKLTKLDLSSNILEGHLPSSMGDLASLKILYLQDNKLT 200
AT1G11130.1_Acc_ICE111         LSGEIPDYFQQLSKLTKLDLSSNILEGHLPSSMGDLASLKILYLQDNKLT 200
AT1G11130.1_Acc_ICE106         LSGEIPDYFQQLSKLTKLDLSSNILEGHLPSSMGDLASLKILYLQDNKLT 200
AT1G11130.1_Acc_ICE104         LSGEIPDYFQQLSKLTKLDLSSNILEGHLPSSMGDLASLKILYLQDNKLT 200
AT1G11130.1_Acc_ICE1           LSGEIPDYFQQLSKLTKLDLSSNILEGHLPSSMGDLASLKILYLQDNKLT 200
AT1G11130.1_Acc_Fei-0          LSGEIPDYFQQLSKLTKLDLSSNILEGHLPSSMGDLASLKILYLQDNKLT 200
AT1G11130.1_Acc_Dog-4          LSGEIPDYFQQLSKLTKLDLSSNILEGHLPSSMGDLASLKILYLQDNKLT 200
AT1G11130.1_Acc_Cdm-0          LSGEIPDYFQQLSKLTKLDLSSNILEGHLPSSMGDLASLKILYLQDNKLT 200
AT1G11130.1_Acc_Bak-2          LSGEIPDYFQQLSKLTKLDLSSNILEGHLPSSMGDLASLKILYLQDNKLT 200
AT1G11130.1_Acc_Agu-1          LSGEIPDYFQQLSKLTKLDLSSNILEGHLPSSMGDLASLKILYLQDNKLT 200
AT1G11130.1_REF                LSGEIPDYFQQLSKLTKLDLSSNILEGHLPSSMGDLASLKILYLQDNKLT 200
AT1G11130.1_Acc_ICE120         LSGEIPDYFQQLSKLTKLDLSSNILEGHLPSSMGDLASLKILYLQDNKLT 200
AT1G11130.1_Acc_Tuescha-9      LSGEIPDYFQQLSKLTKLDLSSNILEGHLPSSMGDLASLKILYLQDNKLT 200
AT1G11130.1_Acc_TueWa1-2       LSGEIPDYFQQLSKLTKLDLSSNILEGHLPSSMGDLASLKILYLQDNKLT 200
AT1G11130.1_Acc_Vash-1         LSGEIPDYFQQLSKLTKLDLSSNILEGHLPSSMGDLASLKILYLQDNKLT 200
AT1G11130.1_Acc_Pra-6          LSGEIPDYFQQLSKLTKLDLSSNILEGHLPSSMGDLASLKILYLQDNKLT 200
AT1G11130.1_Acc_ICE102         LSGEIPDYFQQLSKLTKLDLSSNILEGHLPSSMGDLASLKILYLQDNKLT 200
AT1G11130.1_Acc_Qui-0          LSGEIPDYFQQLSKLTKLDLSSNILEGHLPSSMGDLASLKILYLQDNKLT 200
AT1G11130.1_Acc_ICE61          LSGEIPDYFQQLSKLTKLDLSSNILEGHLPSSMGDLASLKILYLQDNKLT 200
                               **************************************************

AT1G11130.1_Acc_Del-10         GTLDVIEDLFLTDLNVENNLFSGPIPPNLLKIPNFKKDGTPFNTSIITPP 250
AT1G11130.1_Acc_ICE107         GTLDVIEDLFLTDLNVENNLFSGPIPPNLLKIPNFKKDGTPFNTSIITPP 250
AT1G11130.1_Acc_Nie1-2         GTLDVIEDLFLTDLNVENNLFSGPIPPNLLKIPNFKKDGTPFNTSIITPP 250
AT1G11130.1_Acc_Ped-0          GTLDVIEDLFLTDLNVENNLFSGPIPPNLLKIPNFKKDGTPFNTSIITPP 250
AT1G11130.1_Acc_Koch-1         GTLDVIEDLFLTDLNVENNLFSGPIPPNLLKIPNFKKDGTPFNTSIITPP 250
AT1G11130.1_Acc_TueSB30-3      GTLDVIEDLFLTDLNVENNLFSGPIPPNLLKIPNFKKDGTPFNTSIITPP 250
AT1G11130.1_Acc_Yeg-1          GTLDVIEDLFLTDLNVENNLFSGPIPPNLLKIPNFKKDGTPFNTSIITPP 250
AT1G11130.1_Acc_ICE216         GTLDVIEDLFLTDLNVENNLFSGPIPPNLLKIPNFKKDGTPFNTSIITPP 250
AT1G11130.1_Acc_ICE72          GTLDVIEDLFLTDLNVENNLFSGPIPPNLLKIPNFKKDGTPFNTSIITPP 250
AT1G11130.1_Acc_ICE50          GTLDVIEDLFLTDLNVENNLFSGPIPPNLLKIPNFKKDGTPFNTSIITPP 250
AT1G11130.1_Acc_ICE60          GTLDVIEDLFLTDLNVENNLFSGPIPPNLLKIPNFKKDGTPFNTSIITPP 250
AT1G11130.1_Acc_Mer-6          GTLDVIEDLFLTDLNVENNLFSGPIPPNLLKIPNFKKDGTPFNTSIITPP 250
AT1G11130.1_Acc_Nemrut-1       GTLDVIEDLFLTDLNVENNLFSGPIPPNLLKIPNFKKDGTPFNTSIITPP 250
AT1G11130.1_Acc_Sha            GTLDVIEDLFLTDLNVENNLFSGPIPPNLLKIPNFKKDGTPFNTSIITPP 250
AT1G11130.1_Acc_Rue3-1-31      GTLDVIEDLFLTDLNVENNLFSGPIPPNLLKIPNFKKDGTPFNTSIITPP 250
AT1G11130.1_Acc_Leo-1          GTLDVIEDLFLTDLNVENNLFSGPIPPNLLKIPNFKKDGTPFNTSIITPP 250
AT1G11130.1_Acc_Istisu-1       GTLDVIEDLFLTDLNVENNLFSGPIPPNLLKIPNFKKDGTPFNTSIITPP 250
AT1G11130.1_Acc_ICE98          GTLDVIEDLFLTDLNVENNLFSGPIPPNLLKIPNFKKDGTPFNTSIITPP 250
AT1G11130.1_Acc_ICE97          GTLDVIEDLFLTDLNVENNLFSGPIPPNLLKIPNFKKDGTPFNTSIITPP 250
AT1G11130.1_Acc_ICE92          GTLDVIEDLFLTDLNVENNLFSGPIPPNLLKIPNFKKDGTPFNTSIITPP 250
AT1G11130.1_Acc_ICE79          GTLDVIEDLFLTDLNVENNLFSGPIPPNLLKIPNFKKDGTPFNTSIITPP 250
AT1G11130.1_Acc_ICE75          GTLDVIEDLFLTDLNVENNLFSGPIPPNLLKIPNFKKDGTPFNTSIITPP 250
AT1G11130.1_Acc_ICE71          GTLDVIEDLFLTDLNVENNLFSGPIPPNLLKIPNFKKDGTPFNTSIITPP 250
AT1G11130.1_Acc_ICE7           GTLDVIEDLFLTDLNVENNLFSGPIPPNLLKIPNFKKDGTPFNTSIITPP 250
AT1G11130.1_Acc_ICE49          GTLDVIEDLFLTDLNVENNLFSGPIPPNLLKIPNFKKDGTPFNTSIITPP 250
AT1G11130.1_Acc_ICE36          GTLDVIEDLFLTDLNVENNLFSGPIPPNLLKIPNFKKDGTPFNTSIITPP 250
AT1G11130.1_Acc_ICE33          GTLDVIEDLFLTDLNVENNLFSGPIPPNLLKIPNFKKDGTPFNTSIITPP 250
AT1G11130.1_Acc_ICE228         GTLDVIEDLFLTDLNVENNLFSGPIPPNLLKIPNFKKDGTPFNTSIITPP 250
AT1G11130.1_Acc_ICE213         GTLDVIEDLFLTDLNVENNLFSGPIPPNLLKIPNFKKDGTPFNTSIITPP 250
AT1G11130.1_Acc_ICE212         GTLDVIEDLFLTDLNVENNLFSGPIPPNLLKIPNFKKDGTPFNTSIITPP 250
AT1G11130.1_Acc_ICE21          GTLDVIEDLFLTDLNVENNLFSGPIPPNLLKIPNFKKDGTPFNTSIITPP 250
AT1G11130.1_Acc_ICE173         GTLDVIEDLFLTDLNVENNLFSGPIPPNLLKIPNFKKDGTPFNTSIITPP 250
AT1G11130.1_Acc_ICE169         GTLDVIEDLFLTDLNVENNLFSGPIPPNLLKIPNFKKDGTPFNTSIITPP 250
AT1G11130.1_Acc_ICE153         GTLDVIEDLFLTDLNVENNLFSGPIPPNLLKIPNFKKDGTPFNTSIITPP 250
AT1G11130.1_Acc_ICE150         GTLDVIEDLFLTDLNVENNLFSGPIPPNLLKIPNFKKDGTPFNTSIITPP 250
AT1G11130.1_Acc_ICE138         GTLDVIEDLFLTDLNVENNLFSGPIPPNLLKIPNFKKDGTPFNTSIITPP 250
AT1G11130.1_Acc_ICE134         GTLDVIEDLFLTDLNVENNLFSGPIPPNLLKIPNFKKDGTPFNTSIITPP 250
AT1G11130.1_Acc_ICE130         GTLDVIEDLFLTDLNVENNLFSGPIPPNLLKIPNFKKDGTPFNTSIITPP 250
AT1G11130.1_Acc_ICE119         GTLDVIEDLFLTDLNVENNLFSGPIPPNLLKIPNFKKDGTPFNTSIITPP 250
AT1G11130.1_Acc_ICE111         GTLDVIEDLFLTDLNVENNLFSGPIPPNLLKIPNFKKDGTPFNTSIITPP 250
AT1G11130.1_Acc_ICE106         GTLDVIEDLFLTDLNVENNLFSGPIPPNLLKIPNFKKDGTPFNTSIITPP 250
AT1G11130.1_Acc_ICE104         GTLDVIEDLFLTDLNVENNLFSGPIPPNLLKIPNFKKDGTPFNTSIITPP 250
AT1G11130.1_Acc_ICE1           GTLDVIEDLFLTDLNVENNLFSGPIPPNLLKIPNFKKDGTPFNTSIITPP 250
```

```
AT1G11130.1_Acc_Fei-0         GTLDVIEDLFLTDLNVENNLFSGPIPPNLLKIPNFKKDGTPFNTSIITPP 250
AT1G11130.1_Acc_Dog-4         GTLDVIEDLFLTDLNVENNLFSGPIPPNLLKIPNFKKDGTPFNTSIITPP 250
AT1G11130.1_Acc_Cdm-0         GTLDVIEDLFLTDLNVENNLFSGPIPPNLLKIPNFKKDGTPFNTSIITPP 250
AT1G11130.1_Acc_Bak-2         GTLDVIEDLFLTDLNVENNLFSGPIPPNLLKIPNFKKDGTPFNTSIITPP 250
AT1G11130.1_Acc_Agu-1         GTLDVIEDLFLTDLNVENNLFSGPIPPNLLKIPNFKKDGTPFNTSIITPP 250
AT1G11130.1_REF               GTLDVIEDLFLTDLNVENNLFSGPIPPNLLKIPNFKKDGTPFNTSIITPP 250
AT1G11130.1_Acc_ICE120        GTLDVIEDLFLTDLNVENNLFSGPIPPNLLKIPNFKKDGTPFNTSIITPP 250
AT1G11130.1_Acc_Tuescha-9     GTLDVIEDLFLTDLNVENNLFSGPIPPNLLKIPNFKKDGTPFNTSIITPP 250
AT1G11130.1_Acc_TueWa1-2      GTLDVIEDLFLTDLNVENNLFSGPIPPNLLKIPNFKKDGTPFNTSIITPP 250
AT1G11130.1_Acc_Vash-1        GTLDVIEDLFLTDLNVENNLFSGPIPPNLLKIPNFKKDGTPFNTSIITPP 250
AT1G11130.1_Acc_Pra-6         GTLDVIEDLFLTDLNVENNLFSGPIPPNLLKIPNFKKDGTPFNTSIITPP 250
AT1G11130.1_Acc_ICE102        GTLDVIEDLFLTDLNVENNLFSGPIPPNLLKIPNFKKDGTPFNTSIITPP 250
AT1G11130.1_Acc_Qui-0         GTLDVIEDLFLTDLNVENNLFSGPIPPNLLKIPNFKKDGTPFNTSIITPP 250
AT1G11130.1_Acc_ICE61         GTLDVIEDLFLTDLNVENNLFSGPIPPNLLKIPNFKKDGTPFNTSIITPP 250
                              **************************************************

AT1G11130.1_Acc_Del-10        PPPVVDPPPATHRAPPVPRIPPVSGVPPAPFAPFAPLQPQQHPPPSPPLV 300
AT1G11130.1_Acc_ICE107        PPPVVDPPPATHRAPPVPRIPPVSGVPPAPFAPFAPLQPQQHPPPSPPLV 300
AT1G11130.1_Acc_Nie1-2        PPPVVDPPPATHRAPPVPRIPPVSGVPPAPFAPFAPLQPQQHPPPSPPLV 300
AT1G11130.1_Acc_Ped-0         PPPVVDPPPATHRAPPVPRIPPVSGVPPAPFAPFAPLQPQQHPPPSPPLV 300
AT1G11130.1_Acc_Koch-1        PPPVVDPPPATHRAPPVPRIPPVSGVPPAPFAPFAPLQPQQHPPPSPPLV 300
AT1G11130.1_Acc_TueSB30-3     PPPVVDPPPATHRAPPVPRIPPVSGVPPAPFAPFAPLQPQQHPPPSPPLV 300
AT1G11130.1_Acc_Yeg-1         PPPVVDPPPATHRAPPVPRIPPVSGVPPAPFAPFAPLQPQQHPPPSPPLV 300
AT1G11130.1_Acc_ICE216        PPPVVDPPPATHRAPPVPRIPPVSGVPPAPFAPFAPLQPQQHPPPSPPLV 300
AT1G11130.1_Acc_ICE72         PPPVVDPPPATHRAPPVPRIPPVSGVPPAPFAPFAPLQPQQHPPPSPPLV 300
AT1G11130.1_Acc_ICE50         PPPVVDPPPATHRAPPVPRIPPVSGVPPAPFAPFAPLQPQQHPPPSPPLV 300
AT1G11130.1_Acc_ICE60         PPPVVDPPPATHRAPPVPRIPPVSGVPPAPFAPFAPLQPQQHPPPSPPLV 300
AT1G11130.1_Acc_Mer-6         PPPVVDPPPATHRAPPVPRIPPVSGVPPAPFAPFAPLQPQQHPPPSPPLV 300
AT1G11130.1_Acc_Nemrut-1      PPPVVDPPPATHRAPPVPRIPPVSGVPPAPFAPFAPLQPQQHPPPSPPLV 300
AT1G11130.1_Acc_Sha           PPPVVDPPPATHRAPPVPRIPPVSGVPPAPFAPFAPLQPQQHPPPSPPLV 300
AT1G11130.1_Acc_Rue3-1-31     PPPVVDPPPATHRAPPVPRIPPVSGVPPAPFAPFAPLQPQQHPPPSPPLV 300
AT1G11130.1_Acc_Leo-1         PPPVVDPPPATHRAPPVPRIPPVSGVPPAPFAPFAPLQPQQHPPPSPPLV 300
AT1G11130.1_Acc_Istisu-1      PPPVVDPPPATHRAPPVPRIPPVSGVPPAPFAPFAPLQPQQHPPPSPPLV 300
AT1G11130.1_Acc_ICE98         PPPVVDPPPATHRAPPVPRIPPVSGVPPAPFAPFAPLQPQQHPPPSPPLV 300
AT1G11130.1_Acc_ICE97         PPPVVDPPPATHRAPPVPRIPPVSGVPPAPFAPFAPLQPQQHPPPSPPLV 300
AT1G11130.1_Acc_ICE92         PPPVVDPPPATHRAPPVPRIPPVSGVPPAPFAPFAPLQPQQHPPPSPPLV 300
AT1G11130.1_Acc_ICE79         PPPVVDPPPATHRAPPVPRIPPVSGVPPAPFAPFAPLQPQQHPPPSPPLV 300
AT1G11130.1_Acc_ICE75         PPPVVDPPPATHRAPPVPRIPPVSGVPPAPFAPFAPLQPQQHPPPSPPLV 300
AT1G11130.1_Acc_ICE71         PPPVVDPPPATHRAPPVPRIPPVSGVPPAPFAPFAPLQPQQHPPPSPPLV 300
AT1G11130.1_Acc_ICE7          PPPVVDPPPATHRAPPVPRIPPVSGVPPAPFAPFAPLQPQQHPPPSPPLV 300
AT1G11130.1_Acc_ICE49         PPPVVDPPPATHRAPPVPRIPPVSGVPPAPFAPFAPLQPQQHPPPSPPLV 300
AT1G11130.1_Acc_ICE36         PPPVVDPPPATHRAPPVPRIPPVSGVPPAPFAPFAPLQPQQHPPPSPPLV 300
AT1G11130.1_Acc_ICE33         PPPVVDPPPATHRAPPVPRIPPVSGVPPAPFAPFAPLQPQQHPPPSPPLV 300
AT1G11130.1_Acc_ICE228        PPPVVDPPPATHRAPPVPRIPPVSGVPPAPFAPFAPLQPQQHPPPSPPLV 300
AT1G11130.1_Acc_ICE213        PPPVVDPPPATHRAPPVPRIPPVSGVPPAPFAPFAPLQPQQHPPPSPPLV 300
AT1G11130.1_Acc_ICE212        PPPVVDPPPATHRAPPVPRIPPVSGVPPAPFAPFAPLQPQQHPPPSPPLV 300
AT1G11130.1_Acc_ICE21         PPPVVDPPPATHRAPPVPRIPPVSGVPPAPFAPFAPLQPQQHPPPSPPLV 300
AT1G11130.1_Acc_ICE173        PPPVVDPPPATHRAPPVPRIPPVSGVPPAPFAPFAPLQPQQHPPPSPPLV 300
AT1G11130.1_Acc_ICE169        PPPVVDPPPATHRAPPVPRIPPVSGVPPAPFAPFAPLQPQQHPPPSPPLV 300
AT1G11130.1_Acc_ICE153        PPPVVDPPPATHRAPPVPRIPPVSGVPPAPFAPFAPLQPQQHPPPSPPLV 300
AT1G11130.1_Acc_ICE150        PPPVVDPPPATHRAPPVPRIPPVSGVPPAPFAPFAPLQPQQHPPPSPPLV 300
AT1G11130.1_Acc_ICE138        PPPVVDPPPATHRAPPVPRIPPVSGVPPAPFAPFAPLQPQQHPPPSPPLV 300
AT1G11130.1_Acc_ICE134        PPPVVDPPPATHRAPPVPRIPPVSGVPPAPFAPFAPLQPQQHPPPSPPLV 300
AT1G11130.1_Acc_ICE130        PPPVVDPPPATHRAPPVPRIPPVSGVPPAPFAPFAPLQPQQHPPPSPPLV 300
AT1G11130.1_Acc_ICE119        PPPVVDPPPATHRAPPVPRIPPVSGVPPAPFAPFAPLQPQQHPPPSPPLV 300
AT1G11130.1_Acc_ICE111        PPPVVDPPPATHRAPPVPRIPPVSGVPPAPFAPFAPLQPQQHPPPSPPLV 300
AT1G11130.1_Acc_ICE106        PPPVVDPPPATHRAPPVPRIPPVSGVPPAPFAPFAPLQPQQHPPPSPPLV 300
AT1G11130.1_Acc_ICE104        PPPVVDPPPATHRAPPVPRIPPVSGVPPAPFAPFAPLQPQQHPPPSPPLV 300
AT1G11130.1_Acc_ICE1          PPPVVDPPPATHRAPPVPRIPPVSGVPPAPFAPFAPLQPQQHPPPSPPLV 300
AT1G11130.1_Acc_Fei-0         PPPVVDPPPATHRAPPVPRIPPVSGVPPAPFAPFAPLQPQQHPPPSPPLV 300
AT1G11130.1_Acc_Dog-4         PPPVVDPPPATHRAPPVPRIPPVSGVPPAPFAPFAPLQPQQHPPPSPPLV 300
AT1G11130.1_Acc_Cdm-0         PPPVVDPPPATHRAPPVPRIPPVSGVPPAPFAPFAPLQPQQHPPPSPPLV 300
AT1G11130.1_Acc_Bak-2         PPPVVDPPPATHRAPPVPRIPPVSGVPPAPFAPFAPLQPQQHPPPSPPLV 300
AT1G11130.1_Acc_Agu-1         PPPVVDPPPATHRAPPVPRIPPVSGVPPAPFAPFAPLQPQQHPPPSPPLV 300
AT1G11130.1_REF               PPPVVDPPPATHRAPPVPRIPPVSGVPPAPFAPFAPLQPQQHPPPSPPLV 300
AT1G11130.1_Acc_ICE120        PPPVVDPPPATHRAPPVPRIPPVSGVPPAPFAPFAPLQPQQHPPPSPPLV 300
AT1G11130.1_Acc_Tuescha-9     PPPVVDPPPATHRAPPVPRIPPVSGVPPAPFAPFAPLQPQQHPPPSPPLV 300
AT1G11130.1_Acc_TueWa1-2      PPPVVDPPPATHRAPPVPRIPPVSGVPPAPFAPFDPLQPQQHPPPSPPLV 300
AT1G11130.1_Acc_Vash-1        PPPVVDPPPATHRAPPVPRIPPVSGVPPAPFAPFDPLQPQQHPPPSPPLV 300
AT1G11130.1_Acc_Pra-6         PPPVVDPPPATHRAPPVPRIPPVSGVPPAPFAPLAPLQPQQHPPPSPPLV 300
AT1G11130.1_Acc_ICE102        PPPVVDPPPATHRAPPVPRIPPVSGVPPAPFAPFAPLQPQQHPPPSPPLV 300
AT1G11130.1_Acc_Qui-0         PPPVVDPPPATHRAPPVPRIPPVSGVPPAPFAPFAPLQPQQHPPPSPPLV 300
AT1G11130.1_Acc_ICE61         PPPVVDPPPATHRAPPVPRIPPVSGVPPAPFAPFAPLQPQQHPPPSPPLV 300
                              *********************************: ***************

AT1G11130.1_Acc_Del-10        WSPPSSDNGGGDPWNSVSGQPTLQISPPSGSGSGKFWSTQRIILVVSSVA 350
AT1G11130.1_Acc_ICE107        WSPPSSDNGGGDPWNSVSGQPTLQISPPSGSGSGKFWSTQRIILVVSSVA 350
AT1G11130.1_Acc_Nie1-2        WSPPSSDNGGGDPWNSVSGQPTLQISPPSGSGSGKFWSTQRIILVVSSVA 350
AT1G11130.1_Acc_Ped-0         WSPPSSDNGGGDPWNSVSGQPTLQISPPSGSGSGKFWSTQRIILVVSSVA 350
AT1G11130.1_Acc_Koch-1        WSPPSSDNGGGDPWNSVSGQPTLQISPPSGSGSGKFWSTQRIILVVSSVA 350
AT1G11130.1_Acc_TueSB30-3     WSPPSSDNGGGDPWNSVSGQPTLQISPPSGSGSGKFWSTQRIILVVSSVA 350
AT1G11130.1_Acc_Yeg-1         WSPPSSDNGGGDPWNSVSGQPTLQISPPSGSGSGKFWSTQRIILVVSSVA 350
AT1G11130.1_Acc_ICE216        WSPPSSDNGGGDPWNSVSGQPTLQISPPSGSGSGKFWSTQRIILVVSSVA 350
AT1G11130.1_Acc_ICE72         WSPPSSDNGGGDPWNSVSGQPTLQISPPSGSGSGKFWSTQRIILVVSSVA 350
AT1G11130.1_Acc_ICE50         WSPPSSDNGGGDPWNSVSGQPTLQISPPSGSGSGKFWSTQRIILVVSSVA 350
AT1G11130.1_Acc_ICE60         WSPPSSDNGGGDPWNSVSGQPTLQISPPSGSGSGKFWSTQRIILVVSSVA 350
AT1G11130.1_Acc_Mer-6         WSPPSSDNGGGDPWNSVSGQPTLQISPPSGSGSGKFWSTQRIILVVSSVA 350
AT1G11130.1_Acc_Nemrut-1      WSPPSSDNGGGDPWNSVSGQPTLQISPPSGSGSGKFWSTQRIILVVSSVA 350
AT1G11130.1_Acc_Sha           WSPPSSDNGGGDPWNSVSGQPTLQISPPSGSGSGKFWSTQRIILVVSSVA 350
AT1G11130.1_Acc_Rue3-1-31     WSPPSSDNGGGDPWNSVSGQPTLQISPPSGSGSGKFWSTQRIILVVSSVA 350
AT1G11130.1_Acc_Leo-1         WSPPSSDNGGGDPWNSVSGQPTLQISPPSGSGSGKFWSTQRIILVVSSVA 350
AT1G11130.1_Acc_Istisu-1      WSPPSSDNGGGDPWNSVSGQPTLQISPPSGSGSGKFWSTQRIILVVSSVA 350
AT1G11130.1_Acc_ICE98         WSPPSSDNGGGDPWNSVSGQPTLQISPPSGSGSGKFWSTQRIILVVSSVA 350
AT1G11130.1_Acc_ICE97         WSPPSSDNGGGDPWNSVSGQPTLQISPPSGSGSGKFWSTQRIILVVSSVA 350
```

```
AT1G11130.1_Acc_ICE92        WSPPSSDNGGGDPWNSVSGQPTLQISPPSGSGSGKFWSTQRIILVVSSVA 350
AT1G11130.1_Acc_ICE79        WSPPSSDNGGGDPWNSVSGQPTLQISPPSGSGSGKFWSTQRIILVVSSVA 350
AT1G11130.1_Acc_ICE75        WSPPSSDNGGGDPWNSVSGQPTLQISPPSGSGSGKFWSTQRIILVVSSVA 350
AT1G11130.1_Acc_ICE71        WSPPSSDNGGGDPWNSVSGQPTLQISPPSGSGSGKFWSTQRIILVVSSVA 350
AT1G11130.1_Acc_ICE7         WSPPSSDNGGGDPWNSVSGQPTLQISPPSGSGSGKFWSTQRIILVVSSVA 350
AT1G11130.1_Acc_ICE49        WSPPSSDNGGGDPWNSVSGQPTLQISPPSGSGSGKFWSTQRIILVVSSVA 350
AT1G11130.1_Acc_ICE36        WSPPSSDNGGGDPWNSVSGQPTLQISPPSGSGSGKFWSTQRIILVVSSVA 350
AT1G11130.1_Acc_ICE33        WSPPSSDNGGGDPWNSVSGQPTLQISPPSGSGSGKFWSTQRIILVVSSVA 350
AT1G11130.1_Acc_ICE228       WSPPSSDNGGGDPWNSVSGQPTLQISPPSGSGSGKFWSTQRIILVVSSVA 350
AT1G11130.1_Acc_ICE213       WSPPSSDNGGGDPWNSVSGQPTLQISPPSGSGSGKFWSTQRIILVVSSVA 350
AT1G11130.1_Acc_ICE212       WSPPSSDNGGGDPWNSVSGQPTLQISPPSGSGSGKFWSTQRIILVVSSVA 350
AT1G11130.1_Acc_ICE21        WSPPSSDNGGGDPWNSVSGQPTLQISPPSGSGSGKFWSTQRIILVVSSVA 350
AT1G11130.1_Acc_ICE173       WSPPSSDNGGGDPWNSVSGQPTLQISPPSGSGSGKFWSTQRIILVVSSVA 350
AT1G11130.1_Acc_ICE169       WSPPSSDNGGGDPWNSVSGQPTLQISPPSGSGSGKFWSTQRIILVVSSVA 350
AT1G11130.1_Acc_ICE153       WSPPSSDNGGGDPWNSVSGQPTLQISPPSGSGSGKFWSTQRIILVVSSVA 350
AT1G11130.1_Acc_ICE150       WSPPSSDNGGGDPWNSVSGQPTLQISPPSGSGSGKFWSTQRIILVVSSVA 350
AT1G11130.1_Acc_ICE138       WSPPSSDNGGGDPWNSVSGQPTLQISPPSGSGSGKFWSTQRIILVVSSVA 350
AT1G11130.1_Acc_ICE134       WSPPSSDNGGGDPWNSVSGQPTLQISPPSGSGSGKFWSTQRIILVVSSVA 350
AT1G11130.1_Acc_ICE130       WSPPSSDNGGGDPWNSVSGQPTLQISPPSGSGSGKFWSTQRIILVVSSVA 350
AT1G11130.1_Acc_ICE119       WSPPSSDNGGGDPWNSVSGQPTLQISPPSGSGSGKFWSTQRIILVVSSVA 350
AT1G11130.1_Acc_ICE111       WSPPSSDNGGGDPWNSVSGQPTLQISPPSGSGSGKFWSTQRIILVVSSVA 350
AT1G11130.1_Acc_ICE106       WSPPSSDNGGGDPWNSVSGQPTLQISPPSGSGSGKFWSTQRIILVVSSVA 350
AT1G11130.1_Acc_ICE104       WSPPSSDNGGGDPWNSVSGQPTLQISPPSGSGSGKFWSTQRIILVVSSVA 350
AT1G11130.1_Acc_ICE1         WSPPSSDNGGGDPWNSVSGQPTLQISPPSGSGSGKFWSTQRIILVVSSVA 350
AT1G11130.1_Acc_Fei-0        WSPPSSDNGGGDPWNSVSGQPTLQISPPSGSGSGKFWSTQRIILVVSSVA 350
AT1G11130.1_Acc_Dog-4        WSPPSSDNGGGDPWNSVSGQPTLQISPPSGSGSGKFWSTQRIILVVSSVA 350
AT1G11130.1_Acc_Cdm-0        WSPPSSDNGGGDPWNSVSGQPTLQISPPSGSGSGKFWSTQRIILVVSSVA 350
AT1G11130.1_Acc_Bak-2        WSPPSSDNGGGDPWNSVSGQPTLQISPPSGSGSGKFWSTQRIILVVSSVA 350
AT1G11130.1_Acc_Agu-1        WSPPSSDNGGGDPWNSVSGQPTLQISPPSGSGSGKFWSTQRIILVVSSVA 350
AT1G11130.1_REF              WSPPSSDNGGGDPWNSVSGQPTLQISPPSGSGSGKFWSTQRIILVVSSVA 350
AT1G11130.1_Acc_ICE120       WSPPSSDNGGGDPWNSVSGQPTLQISPPSGSGSGKFWSTQRIILVVSSMA 350
AT1G11130.1_Acc_Tuescha-9    WSPPSSDNGGGDPWNSVSGQPTLQISPPSGSGSGKFWSTQRIILVVSSVA 350
AT1G11130.1_Acc_TueWa1-2     WSPPSSDNGGGDPWNSVSGQPTLQISPPSGSGSGKFWSTQRIILVVSSVA 350
AT1G11130.1_Acc_Vash-1       WSPPSSDNGGGDPWNSVSGQPTLQISPPSGSGSGKFWSTQRIILVVSSVA 350
AT1G11130.1_Acc_Pra-6        WSPPSSDNGGGDPWNSVSGQPTLQISPPSGSGSGKFWSTQRIILVVSSVA 350
AT1G11130.1_Acc_ICE102       WSPPSSDNGGGDPWNSVSGQPTLQISPPSGSGSGKFWSTQRIILVVSSVA 350
AT1G11130.1_Acc_Qui-0        WSPPSSDNGGGDPWNSVSGQPTLQISPPSGSGSGKFWSTQRIILVVSSVA 350
AT1G11130.1_Acc_ICE61        WSPPSSDNGGGDPWNSVSGQPTLQISPPSGSGSGKFWSTQRIILVVSSVA 350
                             ************************************************:*

AT1G11130.1_Acc_Del-10       IIVLVSGLCVTLWRCCRSKIYNRYYSGARKDLQRPYFNKPPSQPTPTMGK 400
AT1G11130.1_Acc_ICE107       IIVLVSGLCVTLWRCCRSKIYNRYYSGARKDLQRPYFNKPPSQPTPTMGK 400
AT1G11130.1_Acc_Nie1-2       IIVLVSGLCVTLWRCCRSKIYNRYYSGARKDLQRPYFNKPPSQPTPTMGK 400
AT1G11130.1_Acc_Ped-0        IIVLVSGLCVTLWRCCRSKIYNRYYSGARKDLQRPYFNKPPSQPTPTMGK 400
AT1G11130.1_Acc_Koch-1       IIVLVSGLCVTLWRCCRSEIYNRYYSGARKDLQRPYFNKPPSQPTPTMGK 400
AT1G11130.1_Acc_TueSB30-3    IIVLVSGLCVTLWRCCRSKIYNRYYSGARKDLQRPYFNKPPSQPTPTMGK 400
AT1G11130.1_Acc_Yeg-1        IIVLVSGLCVTLWRCCRSKIYNRYYSGARKDLQRPYFNKPPSQPTPTMGK 400
AT1G11130.1_Acc_ICE216       IIVLVSGLCVTLWRCCRSKIYNRYYSGARKDLQRPYFNKPPSQPTPTMGK 400
AT1G11130.1_Acc_ICE72        IIVLVSGLCVTLWRCCRSKIYNRYYSGARKDLQRPYFNKPPSQPTPTMGK 400
AT1G11130.1_Acc_ICE50        IIVLVSGLCVTLWRCCRSKIYNRYYSGARKDLQRPYFNKPPSQPTPTMGK 400
AT1G11130.1_Acc_ICE60        IIVLVSGLCVTLWRCCRSKIYNRYYSGARKDLQRPYFNKPPSQPTPTMGK 400
AT1G11130.1_Acc_Mer-6        IIVLVSGLCVTLWRCCRSKIYNRYYSGARKDLQRPYFNKPPSQPTPTMGK 400
AT1G11130.1_Acc_Nemrut-1     IIVLVSGLCVTLWRCCRSKIYNRYYSGARKDLQRPYFNKPPSQPTPTMGK 400
AT1G11130.1_Acc_Sha          IIVLVSGLCVTLWRCCRSKIYNRYYSGARKDLQRPYFNKPPSQPTPTMGK 400
AT1G11130.1_Acc_Rue3-1-31    IIVLVSGLCVTLWRCCRSKIYNRYYSGARKDLQRPYFNKPPSQPTPTMGK 400
AT1G11130.1_Acc_Leo-1        IIVLVSGLCVTLWRCCRSKIYNRYYSGARKDLQRPYFNKPPSQPTPTMGK 400
AT1G11130.1_Acc_Istisu-1     IIVLVSGLCVTLWRCCRSKIYNRYYSGARKDLQRPYFNKPPSQPTPTMGK 400
AT1G11130.1_Acc_ICE98        IIVLVSGLCVTLWRCCRSKIYNRYYSGARKDLQRPYFNKPPSQPTPTMGK 400
AT1G11130.1_Acc_ICE97        IIVLVSGLCVTLWRCCRSKIYNRYYSGARKDLQRPYFNKPPSQPTPTMGK 400
AT1G11130.1_Acc_ICE92        IIVLVSGLCVTLWRCCRSKIYNRYYSGARKDLQRPYFNKPPSQPTPTMGK 400
AT1G11130.1_Acc_ICE79        IIVLVSGLCVTLWRCCRSKIYNRYYSGARKDLQRPYFNKPPSQPTPTMGK 400
AT1G11130.1_Acc_ICE75        IIVLVSGLCVTLWRCCRSKIYNRYYSGARKDLQRPYFNKPPSQPTPTMGK 400
AT1G11130.1_Acc_ICE71        IIVLVSGLCVTLWRCCRSKIYNRYYSGARKDLQRPYFNKPPSQPTPTMGK 400
AT1G11130.1_Acc_ICE7         IIVLVSGLCVTLWRCCRSKIYNRYYSGARKDLQRPYFNKPPSQPTPTMGK 400
AT1G11130.1_Acc_ICE49        IIVLVSGLCVTLWRCCRSKIYNRYYSGARKDLQRPYFNKPPSQPTPTMGK 400
AT1G11130.1_Acc_ICE36        IIVLVSGLCVTLWRCCRSKIYNRYYSGARKDLQRPYFNKPPSQPTPTMGK 400
AT1G11130.1_Acc_ICE33        IIVLVSGLCVTLWRCCRSKIYNRYYSGARKDLQRPYFNKPPSQPTPTMGK 400
AT1G11130.1_Acc_ICE228       IIVLVSGLCVTLWRCCRSKIYNRYYSGARKDLQRPYFNKPPSQPTPTMGK 400
AT1G11130.1_Acc_ICE213       IIVLVSGLCVTLWRCCRSKIYNRYYSGARKDLQRPYFNKPPSQPTPTMGK 400
AT1G11130.1_Acc_ICE212       IIVLVSGLCVTLWRCCRSKIYNRYYSGARKDLQRPYFNKPPSQPTPTMGK 400
AT1G11130.1_Acc_ICE21        IIVLVSGLCVTLWRCCRSKIYNRYYSGARKDLQRPYFNKPPSQPTPTMGK 400
AT1G11130.1_Acc_ICE173       IIVLVSGLCVTLWRCCRSKIYNRYYSGARKDLQRPYFNKPPSQPTPTMGK 400
AT1G11130.1_Acc_ICE169       IIVLVSGLCVTLWRCCRSKIYNRYYSGARKDLQRPYFNKPPSQPTPTMGK 400
AT1G11130.1_Acc_ICE153       IIVLVSGLCVTLWRCCRSKIYNRYYSGARKDLQRPYFNKPPSQPTPTMGK 400
AT1G11130.1_Acc_ICE150       IIVLVSGLCVTLWRCCRSKIYNRYYSGARKDLQRPYFNKPPSQPTPTMGK 400
AT1G11130.1_Acc_ICE138       IIVLVSGLCVTLWRCCRSKIYNRYYSGARKDLQRPYFNKPPSQPTPTMGK 400
AT1G11130.1_Acc_ICE134       IIVLVSGLCVTLWRCCRSKIYNRYYSGARKDLQRPYFNKPPSQPTPTMGK 400
AT1G11130.1_Acc_ICE130       IIVLVSGLCVTLWRCCRSKIYNRYYSGARKDLQRPYFNKPPSQPTPTMGK 400
AT1G11130.1_Acc_ICE119       IIVLVSGLCVTLWRCCRSKIYNRYYSGARKDLQRPYFNKPPSQPTPTMGK 400
AT1G11130.1_Acc_ICE111       IIVLVSGLCVTLWRCCRSKIYNRYYSGARKDLQRPYFNKPPSQPTPTMGK 400
AT1G11130.1_Acc_ICE106       IIVLVSGLCVTLWRCCRSKIYNRYYSGARKDLQRPYFNKPPSQPTPTMGK 400
AT1G11130.1_Acc_ICE104       IIVLVSGLCVTLWRCCRSKIYNRYYSGARKDLQRPYFNKPPSQPTPTMGK 400
AT1G11130.1_Acc_ICE1         IIVLVSGLCVTLWRCCRSKIYNRYYSGARKDLQRPYFNKPPSQPTPTMGK 400
AT1G11130.1_Acc_Fei-0        IIVLVSGLCVTLWRCCRSKIYNRYYSGARKDLQRPYFNKPPSQPTPTMGK 400
AT1G11130.1_Acc_Dog-4        IIVLVSGLCVTLWRCCRSKIYNRYYSGARKDLQRPYFNKPPSQPTPTMGK 400
AT1G11130.1_Acc_Cdm-0        IIVLVSGLCVTLWRCCRSKIYNRYYSGARKDLQRPYFNKPPSQPTPTMGK 400
AT1G11130.1_Acc_Bak-2        IIVLVSGLCVTLWRCCRSKIYNRYYSGARKDLQRPYFNKPPSQPTPTMGK 400
AT1G11130.1_Acc_Agu-1        IIVLVSGLCVTLWRCCRSKIYNRYYSGARKDLQRPYFNKPPSQPTPTMGK 400
AT1G11130.1_REF              IIVLVSGLCVTLWRCCRSKIYNRYYSGARKDLQRPYFNKPPSQPTPTMGK 400
AT1G11130.1_Acc_ICE120       IIVLVSGLCVTLWRCCRSKIYNRYYSGARKDLQRPYFNKPPSQPTPTMGK 400
AT1G11130.1_Acc_Tuescha-9    IIVLVSGLCVTLWRCCRSKIYNRYYSGARKDLQRPYFNKPPSQPTPTMGK 400
AT1G11130.1_Acc_TueWa1-2     IIVLVSGLCVTLWRCCRSKIYNRYYSGARKDLQRPYFNKPPSQPTPTMGK 400
AT1G11130.1_Acc_Vash-1       IIVLVSGLCVTLWRCCRSKIYNRYYSGARKDLQRPYFNKPPSQPTPTMGK 400
AT1G11130.1_Acc_Pra-6        IIVLVSGLCVTLWRCCRSKIYNRYYSGARKDLQRPYFNKPPSQPTPTMGK 400
```

```
AT1G11130.1_Acc_ICE102         IIVLVSGLCVTLWRCCRSKIYNRYYSGARKDLQRPYFNKPPSQPTPTMGK 400
AT1G11130.1_Acc_Qui-0          IIVLVSGLCVTLWRCCRSKIYNRYYSGARKDLQRPYFNKPPSQPTPTMGK 400
AT1G11130.1_Acc_ICE61          IIVLVSGLCVTLWRCCRSKIYNRYYSGARKDLQRPYFNKPPSQPTPTMGK 400
                               ******************:*******************************

AT1G11130.1_Acc_Del-10         VSREPMVKPFDGYGAGDRKYGYPMPQRAEESRRAMPPTSYYNKDVNTPQK 450
AT1G11130.1_Acc_ICE107         VSREPMVKPFDGYGAGDRKYGYPMPQRAEESRRAMPPTSYYNKDVNTPQK 450
AT1G11130.1_Acc_Nie1-2         VSREPMVKPFDGYGAGDRKYGYPMPQRAEESRRAMPPTSYYNKDVNTPQK 450
AT1G11130.1_Acc_Ped-0          VSREPMVKPFDGYGAGDRKYGYPMPQRAEESRRAMPPTSYYNKDVNTPQK 450
AT1G11130.1_Acc_Koch-1         VSREPMVKPFDGYGAGDRKYGYPMPQRAEESRRAMPPTSYYNKDVNTPQK 450
AT1G11130.1_Acc_TueSB30-3      VSREPMVKPFDGYGAGDRKYGYPMPQRAEESRRAMPPTSYYNKDVNTPQK 450
AT1G11130.1_Acc_Yeg-1          VSREPMVKPFDGYGAGDRKYGYPMPQRAEESRRAMPPTSYYNKDVNTPQK 450
AT1G11130.1_Acc_ICE216         VSREPMVKPFDGYGAGDRKYGYPMPQRAEESRRAMPPTSYYNKDVNTPQK 450
AT1G11130.1_Acc_ICE72          VSREPMVKPFDGYGAGDRKYGYPMPQRAEESRRAMPPTSYYNKDVNTPQK 450
AT1G11130.1_Acc_ICE50          VSREPMVKPFDGYGAGDRKYGYPMPQRAEESRRAMPPTSYYNKDVNTPQK 450
AT1G11130.1_Acc_ICE60          VSREPMVKPFDGYGAGDRKYGYPMPQRAEESRRAMPPTSYYNKDVNTPQK 450
AT1G11130.1_Acc_Mer-6          VSREPMVKPFDGYGAGDRKYGYPMPQRAEESRRAMPPTSYYNKDVNTPQK 450
AT1G11130.1_Acc_Nemrut-1       VSREPMVKPFDGYGAGDRKYGYPMPQRAEESRRAMPPTSYYNKDVNTPQK 450
AT1G11130.1_Acc_Sha            VSREPMVKPFDGYGAGDRKYGYPMPQRAEESRRAMPPTSYYNKDVNTPQK 450
AT1G11130.1_Acc_Rue3-1-31      VSREPMVKPFDGYGAGDRKYGYPMPQRAEESRRAMPPTSYYNKDVNTPQK 450
AT1G11130.1_Acc_Leo-1          VSREPMVKPFDGYGAGDRKYGYPMPQRAEESRRAMPPTSYYNKDVNTPQK 450
AT1G11130.1_Acc_Istisu-1       VSREPMVKPFDGYGAGDRKYGYPMPQRAEESRRAMPPTSYYNKDVNTPQK 450
AT1G11130.1_Acc_ICE98          VSREPMVKPFDGYGAGDRKYGYPMPQRAEESRRAMPPTSYYNKDVNTPQK 450
AT1G11130.1_Acc_ICE97          VSREPMVKPFDGYGAGDRKYGYPMPQRAEESRRAMPPTSYYNKDVNTPQK 450
AT1G11130.1_Acc_ICE92          VSREPMVKPFDGYGAGDRKYGYPMPQRAEESRRAMPPTSYYNKDVNTPQK 450
AT1G11130.1_Acc_ICE79          VSREPMVKPFDGYGAGDRKYGYPMPQRAEESRRAMPPTSYYNKDVNTPQK 450
AT1G11130.1_Acc_ICE75          VSREPMVKPFDGYGAGDRKYGYPMPQRAEESRRAMPPTSYYNKDVNTPQK 450
AT1G11130.1_Acc_ICE71          VSREPMVKPFDGYGAGDRKYGYPMPQRAEESRRAMPPTSYYNKDVNTPQK 450
AT1G11130.1_Acc_ICE7           VSREPMVKPFDGYGAGDRKYGYPMPQRAEESRRAMPPTSYYNKDVNTPQK 450
AT1G11130.1_Acc_ICE49          VSREPMVKPFDGYGAGDRKYGYPMPQRAEESRRAMPPTSYYNKDVNTPQK 450
AT1G11130.1_Acc_ICE36          VSREPMVKPFDGYGAGDRKYGYPMPQRAEESRRAMPPTSYYNKDVNTPQK 450
AT1G11130.1_Acc_ICE33          VSREPMVKPFDGYGAGDRKYGYPMPQRAEESRRAMPPTSYYNKDVNTPQK 450
AT1G11130.1_Acc_ICE228         VSREPMVKPFDGYGAGDRKYGYPMPQRAEESRRAMPPTSYYNKDVNTPQK 450
AT1G11130.1_Acc_ICE213         VSREPMVKPFDGYGAGDRKYGYPMPQRAEESRRAMPPTSYYNKDVNTPQK 450
AT1G11130.1_Acc_ICE212         VSREPMVKPFDGYGAGDRKYGYPMPQRAEESRRAMPPTSYYNKDVNTPQK 450
AT1G11130.1_Acc_ICE21          VSREPMVKPFDGYGAGDRKYGYPMPQRAEESRRAMPPTSYYNKDVNTPQK 450
AT1G11130.1_Acc_ICE173         VSREPMVKPFDGYGAGDRKYGYPMPQRAEESRRAMPPTSYYNKDVNTPQK 450
AT1G11130.1_Acc_ICE169         VSREPMVKPFDGYGAGDRKYGYPMPQRAEESRRAMPPTSYYNKDVNTPQK 450
AT1G11130.1_Acc_ICE153         VSREPMVKPFDGYGAGDRKYGYPMPQRAEESRRAMPPTSYYNKDVNTPQK 450
AT1G11130.1_Acc_ICE150         VSREPMVKPFDGYGAGDRKYGYPMPQRAEESRRAMPPTSYYNKDVNTPQK 450
AT1G11130.1_Acc_ICE138         VSREPMVKPFDGYGAGDRKYGYPMPQRAEESRRAMPPTSYYNKDVNTPQK 450
AT1G11130.1_Acc_ICE134         VSREPMVKPFDGYGAGDRKYGYPMPQRAEESRRAMPPTSYYNKDVNTPQK 450
AT1G11130.1_Acc_ICE130         VSREPMVKPFDGYGAGDRKYGYPMPQRAEESRRAMPPTSYYNKDVNTPQK 450
AT1G11130.1_Acc_ICE119         VSREPMVKPFDGYGAGDRKYGYPMPQRAEESRRAMPPTSYYNKDVNTPQK 450
AT1G11130.1_Acc_ICE111         VSREPMVKPFDGYGAGDRKYGYPMPQRAEESRRAMPPTSYYNKDVNTPQK 450
AT1G11130.1_Acc_ICE106         VSREPMVKPFDGYGAGDRKYGYPMPQRAEESRRAMPPTSYYNKDVNTPQK 450
AT1G11130.1_Acc_ICE104         VSREPMVKPFDGYGAGDRKYGYPMPQRAEESRRAMPPTSYYNKDVNTPQK 450
AT1G11130.1_Acc_ICE1           VSREPMVKPFDGYGAGDRKYGYPMPQRAEESRRAMPPTSYYNKDVNTPQK 450
AT1G11130.1_Acc_Fei-0          VSREPMVKPFDGYGAGDRKYGYPMPQRAEESRRAMPPTSYYNKDVNTPQK 450
AT1G11130.1_Acc_Dog-4          VSREPMVKPFDGYGAGDRKYGYPMPQRAEESRRAMPPTSYYNKDVNTPQK 450
AT1G11130.1_Acc_Cdm-0          VSREPMVKPFDGYGAGDRKYGYPMPQRAEESRRAMPPTSYYNKDVNTPQK 450
AT1G11130.1_Acc_Bak-2          VSREPMVKPFDGYGAGDRKYGYPMPQRAEESRRAMPPTSYYNKDVNTPQK 450
AT1G11130.1_Acc_Agu-1          VSREPMVKPFDGYGAGDRKYGYPMPQRAEESRRAMPPTSYYNKDVNTPQK 450
AT1G11130.1_REF                VSREPMVKPFDGYGAGDRKYGYPMPQRAEESRRAMPPTSYYNKDVNTPQK 450
AT1G11130.1_Acc_ICE120         VSREPMVKPFDGYGAGDRKYGYPMPQRAEESRRAMPPTSYYNKDVNTPQK 450
AT1G11130.1_Acc_Tuescha-9      VSREPMVKPFDGYGAGDRKYGYPMPQRAEESRRAMPPTSYYNKDVNTPQK 450
AT1G11130.1_Acc_TueWa1-2       VSREPMVKPFDGYGAGDRKYGYPMPQRAEESRRAMPPTSYYNKDVNTPQK 450
AT1G11130.1_Acc_Vash-1         VSREPMVKPFDGYGAGDRKYGYPMPQRAEESRRAMPPTSYYNKDVNTPQK 450
AT1G11130.1_Acc_Pra-6          VSREPMVKPFDGYGAGDRKYGYPMPQRAEESRRAMPPTSYYNKDVNTPQK 450
AT1G11130.1_Acc_ICE102         VSREPMVKPFDGYGAGDRKYGYPMPQRAEESRRAMPPTSYYNKDVNTPQK 450
AT1G11130.1_Acc_Qui-0          VSREPMVKPFDGYGAGDRKYGYPMPQRAEESRRAMPPTSYYNKDVNTPQK 450
AT1G11130.1_Acc_ICE61          VSREPMVKPFDGYGAGDRKYGYPMPRRAEESRRAMPPTSYYNKDVNTPQK 450
                               *************************:************************

AT1G11130.1_Acc_Del-10         PLQQPPRQFQSNDTASKRAAHFPPGLNSSSSATVFTIASLQQYTNNFSEE 500
AT1G11130.1_Acc_ICE107         PLQQPPRQFQSNDTASKRAAHFPPGLNSSSSATVFTIASLQQYTNNFSEE 500
AT1G11130.1_Acc_Nie1-2         PLQQPPRQFQSNDTASKRAAHFPPGLNSSSSATVFTIASLQQYTNNFSEE 500
AT1G11130.1_Acc_Ped-0          PLQQPPRQFQSNDTASKRAAHFPPGLNSSSSATVFTIASLQQYTNNFSEE 500
AT1G11130.1_Acc_Koch-1         PLQQPPRQFQSNDTASKRAAHFPPGLNSSSSATVFTIASLQQYTNNFSEE 500
AT1G11130.1_Acc_TueSB30-3      PLQQPPRQFQSNDTASKRAAHFPPGLNSSSSATVFTIASLQQYTNNFSEE 500
AT1G11130.1_Acc_Yeg-1          PLQQPPRQFQSNDTASKRAAHFPPGLNSSSSATVFTIASLQQYTNNFSEE 500
AT1G11130.1_Acc_ICE216         PLQQPPRQFQSNDTASKRAAHFPPGLNSSSSATVFTIASLQQYTNNFSEE 500
AT1G11130.1_Acc_ICE72          PLQQPLRQFQSNDTASKRAAHFPPGLNSSSSATVFTIASLQQYTNNFSEE 500
AT1G11130.1_Acc_ICE50          PLQQPPRQFQSNDTASKRAAHFPPGLNSSSSATVFTIASLQQYTNNFSEE 500
AT1G11130.1_Acc_ICE60          PLQQPPRQFQSNDTASKRAAHFPPGLNSSSSATVFTIASLQQYTNNFSEE 500
AT1G11130.1_Acc_Mer-6          PLQQPPRQFQSNDTASKRAAHFPPGLNSSSSATVFTIASLQQYTNNFSEE 500
AT1G11130.1_Acc_Nemrut-1       PLQQPPRQFQSNDTASKRAAHFPPGLNSSSSATVFTIASLQQYTNNFSEE 500
AT1G11130.1_Acc_Sha            PLQQPPRQFQSNDTASKRAAHFPPGLNSSSSATVFTIASLQQYTNNFSEE 500
AT1G11130.1_Acc_Rue3-1-31      PLQQPPRQFQSNDTASKRAAHFPPGLNSSSSATVFTIASLQQYTNNFSEE 500
AT1G11130.1_Acc_Leo-1          PLQQPPRQFQSNDTASKRAAHFPPGLNSSSSATVFTIASLQQYTNNFSEE 500
AT1G11130.1_Acc_Istisu-1       PLQQPPRQFQSNDTASKRAAHFPPGLNSSSSATVFTIASLQQYTNNFSEE 500
AT1G11130.1_Acc_ICE98          PLQQPPRQFQSNDTASKRAAHFPPGLNSSSSATVFTIASLQQYTNNFSEE 500
AT1G11130.1_Acc_ICE97          PLQQPPRQFQSNDTASKRAAHFPPGLNSSSSATVFTIASLQQYTNNFSEE 500
AT1G11130.1_Acc_ICE92          PLQQPPRQFQSNDTASKRAAHFPPGLNSSSSATVFTIASLQQYTNNFSEE 500
AT1G11130.1_Acc_ICE79          PLQQPPRQFQSNDTASKRAAHFPPGLNSSSSATVFTIASLQQYTNNFSEE 500
AT1G11130.1_Acc_ICE75          PLQQPPRQFQSNDTASKRAAHFPPGLNSSSSATVFTIASLQQYTNNFSEE 500
AT1G11130.1_Acc_ICE71          PLQQPPRQFQSNDTASKRAAHFPPGLNSSSSATVFTIASLQQYTNNFSEE 500
AT1G11130.1_Acc_ICE7           PLQQPPRQFQSNDTASKRAAHFPPGLNSSSSATVFTIASLQQYTNNFSEE 500
AT1G11130.1_Acc_ICE49          PLQQPPRQFQSNDTASKRAAHFPPGLNSSSSATVFTIASLQQYTNNFSEE 500
AT1G11130.1_Acc_ICE36          PLQQPPRQFQSNDTASKRAAHFPPGLNSSSSATVFTIASLQQYTNNFSEE 500
AT1G11130.1_Acc_ICE33          PLQQPPRQFQSNDTASKRAAHFPPGLNSSSSATVFTIASLQQYTNNFSEE 500
AT1G11130.1_Acc_ICE228         PLQQPPRQFQSNDTASKRAAHFPPGLNSSSSATVFTIASLQQYTNNFSEE 500
AT1G11130.1_Acc_ICE213         PLQQPPRQFQSNDTASKRAAHFPPGLNSSSSATVFTIASLQQYTNNFSEE 500
AT1G11130.1_Acc_ICE212         PLQQPPRQFQSNDTASKRAAHFPPGLNSSSSATVFTIASLQQYTNNFSEE 500
```

```
AT1G11130.1_Acc_ICE21         PLQQPPRQFQSNDTASKRAAHFPPGLNSSSSATVFTIASLQQYTNNFSEE 500
AT1G11130.1_Acc_ICE173        PLQQPPRQFQSNDTASKRAAHFPPGLNSSSSATVFTIASLQQYTNNFSEE 500
AT1G11130.1_Acc_ICE169        PLQQPPRQFQSNDTASKRAAHFPPGLNSSSSATVFTIASLQQYTNNFSEE 500
AT1G11130.1_Acc_ICE153        PLQQPPRQFQSNDTASKRAAHFPPGLNSSSSATVFTIASLQQYTNNFSEE 500
AT1G11130.1_Acc_ICE150        PLQQPPRQFQSNDTASKRAAHFPPGLNSSSSATVFTIASLQQYTNNFSEE 500
AT1G11130.1_Acc_ICE138        PLQQPPRQFQSNDTASKRAAHFPPGLNSSSSATVFTIASLQQYTNNFSEE 500
AT1G11130.1_Acc_ICE134        PLQQPPRQFQSNDTASKRAAHFPPGLNSSSSATVFTIASLQQYTNNFSEE 500
AT1G11130.1_Acc_ICE130        PLQQPPRQFQSNDTASKRAAHFPPGLNSSSSATVFTIASLQQYTNNFSEE 500
AT1G11130.1_Acc_ICE119        PLQQPPRQFQSNDTASKRAAHFPPGLNSSSSATVFTIASLQQYTNNFSEE 500
AT1G11130.1_Acc_ICE111        PLQQPPRQFQSNDTASKRAAHFPPGLNSSSSATVFTIASLQQYTNNFSEE 500
AT1G11130.1_Acc_ICE106        PLQQPPRQFQSNDTASKRAAHFPPGLNSSSSATVFTIASLQQYTNNFSEE 500
AT1G11130.1_Acc_ICE104        PLQQPPRQFQSNDTASKRAAHFPPGLNSSSSATVFTIASLQQYTNNFSEE 500
AT1G11130.1_Acc_ICE1          PLQQPPRQFQSNDTASKRAAHFPPGLNSSSSATVFTIASLQQYTNNFSEE 500
AT1G11130.1_Acc_Fei-0         PLQQPPRQFQSNDTASKRAAHFPPGLNSSSSATVFTIASLQQYTNNFSEE 500
AT1G11130.1_Acc_Dog-4         PLQQPPRQFQSNDTASKRAAHFPPGLNSSSSATVFTIASLQQYTNNFSEE 500
AT1G11130.1_Acc_Cdm-0         PLQQPPRQFQSNDTASKRAAHFPPGLNSSSSATVFTIASLQQYTNNFSEE 500
AT1G11130.1_Acc_Bak-2         PLQQPPRQFQSNDTASKRAAHFPPGLNSSSSATVFTIASLQQYTNNFSEE 500
AT1G11130.1_Acc_Agu-1         PLQQPPRQFQSNDTASKRAAHFPPGLNSSSSATVFTIASLQQYTNNFSEE 500
AT1G11130.1_REF               PLQQPPRQFQSNDTASKRAAHFPPGLNSSSSATVFTIASLQQYTNNFSEE 500
AT1G11130.1_Acc_ICE120        PLQQPPRQFQSNDTGSKRAAHFPPGLNSSSSATVFTIASLQQYTNNFSEE 500
AT1G11130.1_Acc_Tuescha-9     PLQQPPRQFQSNDTGSKRAAHFPPGLNSSSSATVFTIASLQQYTNNFSEE 500
AT1G11130.1_Acc_TueWa1-2      PLQQPPRQFQSNDTASKRAAHFPPGLNSSSSATVFTIASLQQYTNNFSEE 500
AT1G11130.1_Acc_Vash-1        PLQQPPRQFQSNDTASKRAAHFPPGLNSSSSATVFTIASLQQYTNNFSEE 500
AT1G11130.1_Acc_Pra-6         PLQQPPRQFQSNDTASKRAAHFPPGLNSSSSATVFTIASLQQYTNNFSEE 500
AT1G11130.1_Acc_ICE102        PLQQPPRQFQSNDTASKRAAHFPPGLNSSSSATVFTIASLQQYTNNFSEE 500
AT1G11130.1_Acc_Qui-0         PLQQPPRQFQSNDTASKRAAHFPPGLNSSSSATVFTIASLQQYTNNFSEE 500
AT1G11130.1_Acc_ICE61         PLQQPPRQFQSNDTASKRAAHFPPGLNSSSSATVFTIASLQQYTNNFSEE 500
                              ***** ********.***********************************

AT1G11130.1_Acc_Del-10        NIIGEGSIGNVYRAELRHGKFLAVKKLSNTINRTQSDGEFLNLVSNVLKL 550
AT1G11130.1_Acc_ICE107        NIIGEGSIGNVYRAELRHGKFLAVKKLSNTINRTQSDGEFLNLVSNVLKL 550
AT1G11130.1_Acc_Nie1-2        NIIGEGSIGNVYRAELRHGKFLAVKKLSNTINRTQSDGEFLNLVSNVLKL 550
AT1G11130.1_Acc_Ped-0         NIIGEGSIGNVYRAELRHGKFLAVKKLSNTINRTQSDGEFLNLVSNVLKL 550
AT1G11130.1_Acc_Koch-1        NIIGEGSIGNVYRAELRHGKFLAVKKLSNTINRTQSDGEFLNLVSNVLKL 550
AT1G11130.1_Acc_TueSB30-3     NIIGEGSIGNVYRAELRHGKFLAVKKLSNTINRTQSDGEFLNLVSNVLKL 550
AT1G11130.1_Acc_Yeg-1         NIIGEGSIGNVYRAELRHGKFLAVKKLSNTINRTQSDGEFLNLVSNVLKL 550
AT1G11130.1_Acc_ICE216        NIIGEGSIGNVYRAELRHGKFLAVKKLSNTINRTQSDGEFLNLVSNVLKL 550
AT1G11130.1_Acc_ICE72         NIIGEGSIGNVYRAELRHGKFLAVKKLSNTINRTQSDGEFLNLVSNVLKL 550
AT1G11130.1_Acc_ICE50         NIIGEGSIGNVYRAELRHGKFLAVKKLSNTINRTQSDGEFLNLVSNVLKL 550
AT1G11130.1_Acc_ICE60         NIIGEGSIGNVYRAELRHGKFLAVKKLSNTINRTQSDGEFLNLVSNVLKL 550
AT1G11130.1_Acc_Mer-6         NIIGEGSIGNVYRAELRHGKFLAVKKLSNTINRTQSDGEFLNLVSNVLKL 550
AT1G11130.1_Acc_Nemrut-1      NIIGEGSIGNVYRAELRHGKFLAVKKLSNTINRTQSDGEFLNLVSNVLKL 550
AT1G11130.1_Acc_Sha           NIIGEGSIGNVYRAELRHGKFLAVKKLSNTINRTQSDGEFLNLVSNVLKL 550
AT1G11130.1_Acc_Rue3-1-31     NIIGEGSIGNVYRAELRHGKFLAVKKLSNTINRTQSDGEFLNLVSNVLKL 550
AT1G11130.1_Acc_Leo-1         NIIGEGSIGNVYRAELRHGKFLAVKKLSNTINRTQSDGEFLNLVSNVLKL 550
AT1G11130.1_Acc_Istisu-1      NIIGEGSIGNVYRAELRHGKFLAVKKLSNTINRTQSDGEFLNLVSNVLKL 550
AT1G11130.1_Acc_ICE98         NIIGEGSIGNVYRAELRHGKFLAVKKLSNTINRTQSDGEFLNLVSNVLKL 550
AT1G11130.1_Acc_ICE97         NIIGEGSIGNVYRAELRHGKFLAVKKLSNTINRTQSDGEFLNLVSNVLKL 550
AT1G11130.1_Acc_ICE92         NIIGEGSIGNVYRAELRHGKFLAVKKLSNTINRTQSDGEFLNLVSNVLKL 550
AT1G11130.1_Acc_ICE79         NIIGEGSIGNVYRAELRHGKFLAVKKLSNTINRTQSDGEFLNLVSNVLKL 550
AT1G11130.1_Acc_ICE75         NIIGEGSIGNVYRAELRHGKFLAVKKLSNTINRTQSDGEFLNLVSNVLKL 550
AT1G11130.1_Acc_ICE71         NIIGEGSIGNVYRAELRHGKFLAVKKLSNTINRTQSDGEFLNLVSNVLKL 550
AT1G11130.1_Acc_ICE7          NIIGEGSIGNVYRAELRHGKFLAVKKLSNTINRTQSDGEFLNLVSNVLKL 550
AT1G11130.1_Acc_ICE49         NIIGEGSIGNVYRAELRHGKFLAVKKLSNTINRTQSDGEFLNLVSNVLKL 550
AT1G11130.1_Acc_ICE36         NIIGEGSIGNVYRAELRHGKFLAVKKLSNTINRTQSDGEFLNLVSNVLKL 550
AT1G11130.1_Acc_ICE33         NIIGEGSIGNVYRAELRHGKFLAVKKLSNTINRTQSDGEFLNLVSNVLKL 550
AT1G11130.1_Acc_ICE228        NIIGEGSIGNVYRAELRHGKFLAVKKLSNTINRTQSDGEFLNLVSNVLKL 550
AT1G11130.1_Acc_ICE213        NIIGEGSIGNVYRAELRHGKFLAVKKLSNTINRTQSDGEFLNLVSNVLKL 550
AT1G11130.1_Acc_ICE212        NIIGEGSIGNVYRAELRHGKFLAVKKLSNTINRTQSDGEFLNLVSNVLKL 550
AT1G11130.1_Acc_ICE21         NIIGEGSIGNVYRAELRHGKFLAVKKLSNTINRTQSDGEFLNLVSNVLKL 550
AT1G11130.1_Acc_ICE173        NIIGEGSIGNVYRAELRHGKFLAVKKLSNTINRTQSDGEFLNLVSNVLKL 550
AT1G11130.1_Acc_ICE169        NIIGEGSIGNVYRAELRHGKFLAVKKLSNTINRTQSDGEFLNLVSNVLKL 550
AT1G11130.1_Acc_ICE153        NIIGEGSIGNVYRAELRHGKFLAVKKLSNTINRTQSDGEFLNLVSNVLKL 550
AT1G11130.1_Acc_ICE150        NIIGEGSIGNVYRAELRHGKFLAVKKLSNTINRTQSDGEFLNLVSNVLKL 550
AT1G11130.1_Acc_ICE138        NIIGEGSIGNVYRAELRHGKFLAVKKLSNTINRTQSDGEFLNLVSNVLKL 550
AT1G11130.1_Acc_ICE134        NIIGEGSIGNVYRAELRHGKFLAVKKLSNTINRTQSDGEFLNLVSNVLKL 550
AT1G11130.1_Acc_ICE130        NIIGEGSIGNVYRAELRHGKFLAVKKLSNTINRTQSDGEFLNLVSNVLKL 550
AT1G11130.1_Acc_ICE119        NIIGEGSIGNVYRAELRHGKFLAVKKLSNTINRTQSDGEFLNLVSNVLKL 550
AT1G11130.1_Acc_ICE111        NIIGEGSIGNVYRAELRHGKFLAVKKLSNTINRTQSDGEFLNLVSNVLKL 550
AT1G11130.1_Acc_ICE106        NIIGEGSIGNVYRAELRHGKFLAVKKLSNTINRTQSDGEFLNLVSNVLKL 550
AT1G11130.1_Acc_ICE104        NIIGEGSIGNVYRAELRHGKFLAVKKLSNTINRTQSDGEFLNLVSNVLKL 550
AT1G11130.1_Acc_ICE1          NIIGEGSIGNVYRAELRHGKFLAVKKLSNTINRTQSDGEFLNLVSNVLKL 550
AT1G11130.1_Acc_Fei-0         NIIGEGSIGNVYRAELRHGKFLAVKKLSNTINRTQSDGEFLNLVSNVLKL 550
AT1G11130.1_Acc_Dog-4         NIIGEGSIGNVYRAELRHGKFLAVKKLSNTINRTQSDGEFLNLVSNVLKL 550
AT1G11130.1_Acc_Cdm-0         NIIGEGSIGNVYRAELRHGKFLAVKKLSNTINRTQSDGEFLNLVSNVLKL 550
AT1G11130.1_Acc_Bak-2         NIIGEGSIGNVYRAELRHGKFLAVKKLSNTINRTQSDGEFLNLVSNVLKL 550
AT1G11130.1_Acc_Agu-1         NIIGEGSIGNVYRAELRHGKFLAVKKLSNTINRTQSDGEFLNLVSNVLKL 550
AT1G11130.1_REF               NIIGEGSIGNVYRAELRHGKFLAVKKLSNTINRTQSDGEFLNLVSNVLKL 550
AT1G11130.1_Acc_ICE120        NIIGEGSIGNVYRAELRHGKFLAVKKLSNTINRTQSDGEFLNLVSNVLKL 550
AT1G11130.1_Acc_Tuescha-9     NIIGEGSIGNVYRAELRHGKFLAVKKLSNTINRTQSDGEFLNLVSNVLKL 550
AT1G11130.1_Acc_TueWa1-2      NIIGEGSIGNVYRAELRHGKFLAVKKLSNTINRTQSDGEFLNLVSNVLKL 550
AT1G11130.1_Acc_Vash-1        NIIGEGSIGNVYRAELRHGKFLAVKKLSNTINRTQSDGEFLNLVSNVLKL 550
AT1G11130.1_Acc_Pra-6         NIIGEGSIGNVYRAELRHGKFLAVKKLSNTINRTQSDGEFLNLVSNVLKL 550
AT1G11130.1_Acc_ICE102        NIIGEGSIGNVYRAELRHGKFLAVKKLSNTINRTQSDGEFLNLVSNVLKL 550
AT1G11130.1_Acc_Qui-0         NIIGEGSIGNVYRAELRHGKFLAVKKLSNTINRTQSDGEFLNLVSNVLKL 550
AT1G11130.1_Acc_ICE61         NIIGEGSIGNVYRAELRHGKFLAVKKLSNTINRTQSDGEFLNLVSNVLKL 550
                              **************************************************

AT1G11130.1_Acc_Del-10        KRGHILELLGYCNEFGQRLLVYEYCPNGSLQDALHLDRKLHKKLNWNVRI 600
AT1G11130.1_Acc_ICE107        KRGHILELLGYCNEFGQRLLVYEYCPNGSLQDALHLDRKLHKKLNWNVRI 600
AT1G11130.1_Acc_Nie1-2        KRGHILELLGYCNEFGQRLLVYEYCPNGSLQDALHLDRKLHKKLNWNVRI 600
AT1G11130.1_Acc_Ped-0         KRGHILELLGYCNEFGQRLLVYEYCPNGSLQDALHLDRKLHKKLNWNVRI 600
AT1G11130.1_Acc_Koch-1        KRGHILELLGYCNEFGQRLLVYEYCPNGSLQDALHLDRKLHKKLNWNVRI 600
AT1G11130.1_Acc_TueSB30-3     KRGHILELLGYCNEFGQRLLVYEYCPNGSLQDALHLDRKLHKKLTWNVRI 600
```

```
AT1G11130.1_Acc_Yeg-1          KRGHILELLGYCNEFGQRLLVYEYCPNGSLQDALHLDRKLHKKLTWNVRI 600
AT1G11130.1_Acc_ICE216         KRGHILELLGYCNEFGQRLLVYEYCPNGSLQDALHLDRKLHKKLTWNVRI 600
AT1G11130.1_Acc_ICE72          KRGHILELLGYCNEFGQRLLVYEYCPNGSLQDALHLDRKLHKKLTWNVRI 600
AT1G11130.1_Acc_ICE50          KRGHILELLGYCNEFGQRLLVYEYCPNGSLQDALHLDRKLHKKLTWNVRI 600
AT1G11130.1_Acc_ICE60          KRGHILELLGYCNEFGQRLLVYEYCPNGSLQDALHLDRKLHKKLTWNVRI 600
AT1G11130.1_Acc_Mer-6          KRGHILELLGYCNEFGQRLLVYEYCPNGSLQDALHLDRKLHKKLTWNVRI 600
AT1G11130.1_Acc_Nemrut-1       KRGHILELLGYCNEFGQRLLVYEYCPNGSLQDALHLDRKLHKKLTWNVRI 600
AT1G11130.1_Acc_Sha            KRGHILELLGYCNEFGQRLLVYEYCPNGSLQDALHLDRKLHKKLTWNVRI 600
AT1G11130.1_Acc_Rue3-1-31      KRGHILELLGYCNEFGQRLLVYEYCPNGSLQDALHLDRKLHKKLTWNVRI 600
AT1G11130.1_Acc_Leo-1          KRGHILELLGYCNEFGQRLLVYEYCPNGSLQDALHLDRKLHKKLTWNVRI 600
AT1G11130.1_Acc_Istisu-1       KRGHILELLGYCNEFGQRLLVYEYCPNGSLQDALHLDRKLHKKLTWNVRI 600
AT1G11130.1_Acc_ICE98          KRGHILELLGYCNEFGQRLLVYEYCPNGSLQDALHLDRKLHKKLTWNVRI 600
AT1G11130.1_Acc_ICE97          KRGHILELLGYCNEFGQRLLVYEYCPNGSLQDALHLDRKLHKKLTWNVRI 600
AT1G11130.1_Acc_ICE92          KRGHILELLGYCNEFGQRLLVYEYCPNGSLQDALHLDRKLHKKLTWNVRI 600
AT1G11130.1_Acc_ICE79          KRGHILELLGYCNEFGQRLLVYEYCPNGSLQDALHLDRKLHKKLTWNVRI 600
AT1G11130.1_Acc_ICE75          KRGHILELLGYCNEFGQRLLVYEYCPNGSLQDALHLDRKLHKKLTWNVRI 600
AT1G11130.1_Acc_ICE71          KRGHILELLGYCNEFGQRLLVYEYCPNGSLQDALHLDRKLHKKLTWNVRI 600
AT1G11130.1_Acc_ICE7           KRGHILELLGYCNEFGQRLLVYEYCPNGSLQDALHLDRKLHKKLTWNVRI 600
AT1G11130.1_Acc_ICE49          KRGHILELLGYCNEFGQRLLVYEYCPNGSLQDALHLDRKLHKKLTWNVRI 600
AT1G11130.1_Acc_ICE36          KRGHILELLGYCNEFGQRLLVYEYCPNGSLQDALHLDRKLHKKLTWNVRI 600
AT1G11130.1_Acc_ICE33          KRGHILELLGYCNEFGQRLLVYEYCPNGSLQDALHLDRKLHKKLTWNVRI 600
AT1G11130.1_Acc_ICE228         KRGHILELLGYCNEFGQRLLVYEYCPNGSLQDALHLDRKLHKKLTWNVRI 600
AT1G11130.1_Acc_ICE213         KRGHILELLGYCNEFGQRLLVYEYCPNGSLQDALHLDRKLHKKLTWNVRI 600
AT1G11130.1_Acc_ICE212         KRGHILELLGYCNEFGQRLLVYEYCPNGSLQDALHLDRKLHKKLTWNVRI 600
AT1G11130.1_Acc_ICE21          KRGHILELLGYCNEFGQRLLVYEYCPNGSLQDALHLDRKLHKKLTWNVRI 600
AT1G11130.1_Acc_ICE173         KRGHILELLGYCNEFGQRLLVYEYCPNGSLQDALHLDRKLHKKLTWNVRI 600
AT1G11130.1_Acc_ICE169         KRGHILELLGYCNEFGQRLLVYEYCPNGSLQDALHLDRKLHKKLTWNVRI 600
AT1G11130.1_Acc_ICE153         KRGHILELLGYCNEFGQRLLVYEYCPNGSLQDALHLDRKLHKKLTWNVRI 600
AT1G11130.1_Acc_ICE150         KRGHILELLGYCNEFGQRLLVYEYCPNGSLQDALHLDRKLHKKLTWNVRI 600
AT1G11130.1_Acc_ICE138         KRGHILELLGYCNEFGQRLLVYEYCPNGSLQDALHLDRKLHKKLTWNVRI 600
AT1G11130.1_Acc_ICE134         KRGHILELLGYCNEFGQRLLVYEYCPNGSLQDALHLDRKLHKKLTWNVRI 600
AT1G11130.1_Acc_ICE130         KRGHILELLGYCNEFGQRLLVYEYCPNGSLQDALHLDRKLHKKLTWNVRI 600
AT1G11130.1_Acc_ICE119         KRGHILELLGYCNEFGQRLLVYEYCPNGSLQDALHLDRKLHKKLTWNVRI 600
AT1G11130.1_Acc_ICE111         KRGHILELLGYCNEFGQRLLVYEYCPNGSLQDALHLDRKLHKKLTWNVRI 600
AT1G11130.1_Acc_ICE106         KRGHILELLGYCNEFGQRLLVYEYCPNGSLQDALHLDRKLHKKLTWNVRI 600
AT1G11130.1_Acc_ICE104         KRGHILELLGYCNEFGQRLLVYEYCPNGSLQDALHLDRKLHKKLTWNVRI 600
AT1G11130.1_Acc_ICE1           KRGHILELLGYCNEFGQRLLVYEYCPNGSLQDALHLDRKLHKKLTWNVRI 600
AT1G11130.1_Acc_Fei-0          KRGHILELLGYCNEFGQRLLVYEYCPNGSLQDALHLDRKLHKKLTWNVRI 600
AT1G11130.1_Acc_Dog-4          KRGHILELLGYCNEFGQRLLVYEYCPNGSLQDALHLDRKLHKKLTWNVRI 600
AT1G11130.1_Acc_Cdm-0          KRGHILELLGYCNEFGQRLLVYEYCPNGSLQDALHLDRKLHKKLTWNVRI 600
AT1G11130.1_Acc_Bak-2          KRGHILELLGYCNEFGQRLLVYEYCPNGSLQDALHLDRKLHKKLTWNVRI 600
AT1G11130.1_Acc_Agu-1          KRGHILELLGYCNEFGQRLLVYEYCPNGSLQDALHLDRKLHKKLTWNVRI 600
AT1G11130.1_REF                KRGHILELLGYCNEFGQRLLVYEYCPNGSLQDALHLDRKLHKKLTWNVRI 600
AT1G11130.1_Acc_ICE120         KRGHILELLGYCNEFGQRLLVYEYCPNGSLQDALHLDRKLHKKLTWNVRI 600
AT1G11130.1_Acc_Tuescha-9      KRGHILELLGYCNEFGQRLLVYEYCPNGSLQDALHLDRKLHKKLTWNVRI 600
AT1G11130.1_Acc_TueWa1-2       KRGHILELLGYCNEFGQRLLVYEYCPNGSLQDALHLDRKLHKKLTWNVRI 600
AT1G11130.1_Acc_Vash-1         KRGHILELLGYCNEFGQRLLVYEYCPNGSLQDALHLDRKLHKKLTWNVRI 600
AT1G11130.1_Acc_Pra-6          KRGHILELLGYCNEFGQRLLVYEYCPNGSLQDALHLDRKLHKKLTWNVRI 600
AT1G11130.1_Acc_ICE102         KRGHILELLGYCNEFGQRLLVYEYCPNGSLQDALHLDRKLHKKLTWNVRI 600
AT1G11130.1_Acc_Qui-0          KRGHILELLGYCNEFGQRLLVYEYCPNGSLQDALHLDRKLHKKLTWNVRI 600
AT1G11130.1_Acc_ICE61          KRGHILELLGYCNEFGQRLLVYEYCPNGSLQDALHLDRKLHKKLTWNVRI 600
                               ********************************************.*****

AT1G11130.1_Acc_Del-10         NIALGASKALQFLHEVCQPPVVHQNFKSSKVLLDGKLSVRVADSGLAYML 650
AT1G11130.1_Acc_ICE107         NIALGASKALQFLHEVCQPPVVHQNFKSSKVLLDGKLSVRVADSGLAYML 650
AT1G11130.1_Acc_Nie1-2         NIALGASKALQFLHEVCQPPVVHQNFKSSKVLLDGKLSVRVADSGLAYML 650
AT1G11130.1_Acc_Ped-0          NIALGASKALQFLHEVCQPPVVHQNFKSSKVLLDGKLSVRVADSGLAYML 650
AT1G11130.1_Acc_Koch-1         NIALGASKALQFLHEVCQPPVVHQNFKSSKVLLDGKLSVRVADSGLAYML 650
AT1G11130.1_Acc_TueSB30-3      NIALGASKALQFLHEVCQPPVVHQNFKSSKVLLDGKLSVRVADSGLAYML 650
AT1G11130.1_Acc_Yeg-1          NIALGASKALQFLHEVCQPPVVHQNFKSSKVLLDGKLSVRVADSGLAYML 650
AT1G11130.1_Acc_ICE216         NIALGASKALQFLHEVCQPPVVHQNFKSSKVLLDGKLSVRVADSGLAYML 650
AT1G11130.1_Acc_ICE72          NIALGASKALQFLHEVCQPPVVHQNFKSSKVLLDGKLSVRVADSGLAYML 650
AT1G11130.1_Acc_ICE50          NIALGASKALQFLHEVCQPPVVHQNFKSSKVLLDGKLSVRVADSGLAYML 650
AT1G11130.1_Acc_ICE60          NIALGASKALQFLHEVCQPPVVHQNFKSSKVLLDGKLSVRVADSGLAYML 650
AT1G11130.1_Acc_Mer-6          NIALGASKALQFLHEVCQPPVVHQNFKSSKVLLDGKLSVRVADSGLAYML 650
AT1G11130.1_Acc_Nemrut-1       NIALGASKALQFLHEVCQPPVVHQNFKSSKVLLDGKLSVRVADSGLAYML 650
AT1G11130.1_Acc_Sha            NIALGASKALQFLHEVCQPPVVHQNFKSSKVLLDGKLSVRVADSGLAYML 650
AT1G11130.1_Acc_Rue3-1-31      NIALGASKALQFLHEVCQPPVVHQNFKSSKVLLDGKLSVRVADSGLAYML 650
AT1G11130.1_Acc_Leo-1          NIALGASKALQFLHEVCQPPVVHQNFKSSKVLLDGKLSVRVADSGLAYML 650
AT1G11130.1_Acc_Istisu-1       NIALGASKALQFLHEVCQPPVVHQNFKSSKVLLDGKLSVRVADSGLAYML 650
AT1G11130.1_Acc_ICE98          NIALGASKALQFLHEVCQPPVVHQNFKSSKVLLDGKLSVRVADSGLAYML 650
AT1G11130.1_Acc_ICE97          NIALGASKALQFLHEVCQPPVVHQNFKSSKVLLDGKLSVRVADSGLAYML 650
AT1G11130.1_Acc_ICE92          NIALGASKALQFLHEVCQPPVVHQNFKSSKVLLDGKLSVRVADSGLAYML 650
AT1G11130.1_Acc_ICE79          NIALGASKALQFLHEVCQPPVVHQNFKSSKVLLDGKLSVRVADSGLAYML 650
AT1G11130.1_Acc_ICE75          NIALGASKALQFLHEVCQPPVVHQNFKSSKVLLDGKLSVRVADSGLAYML 650
AT1G11130.1_Acc_ICE71          NIALGASKALQFLHEVCQPPVVHQNFKSSKVLLDGKLSVRVADSGLAYML 650
AT1G11130.1_Acc_ICE7           NIALGASKALQFLHEVCQPPVVHQNFKSSKVLLDGKLSVRVADSGLAYML 650
AT1G11130.1_Acc_ICE49          NIALGASKALQFLHEVCQPPVVHQNFKSSKVLLDGKLSVRVADSGLAYML 650
AT1G11130.1_Acc_ICE36          NIALGASKALQFLHEVCQPPVVHQNFKSSKVLLDGKLSVRVADSGLAYML 650
AT1G11130.1_Acc_ICE33          NIALGASKALQFLHEVCQPPVVHQNFKSSKVLLDGKLSVRVADSGLAYML 650
AT1G11130.1_Acc_ICE228         NIALGASKALQFLHEVCQPPVVHQNFKSSKVLLDGKLSVRVADSGLAYML 650
AT1G11130.1_Acc_ICE213         NIALGASKALQFLHEVCQPPVVHQNFKSSKVLLDGKLSVRVADSGLAYML 650
AT1G11130.1_Acc_ICE212         NIALGASKALQFLHEVCQPPVVHQNFKSSKVLLDGKLSVRVADSGLAYML 650
AT1G11130.1_Acc_ICE21          NIALGASKALQFLHEVCQPPVVHQNFKSSKVLLDGKLSVRVADSGLAYML 650
AT1G11130.1_Acc_ICE173         NIALGASKALQFLHEVCQPPVVHQNFKSSKVLLDGKLSVRVADSGLAYML 650
AT1G11130.1_Acc_ICE169         NIALGASKALQFLHEVCQPPVVHQNFKSSKVLLDGKLSVRVADSGLAYML 650
AT1G11130.1_Acc_ICE153         NIALGASKALQFLHEVCQPPVVHQNFKSSKVLLDGKLSVRVADSGLAYML 650
AT1G11130.1_Acc_ICE150         NIALGASKALQFLHEVCQPPVVHQNFKSSKVLLDGKLSVRVADSGLAYML 650
AT1G11130.1_Acc_ICE138         NIALGASKALQFLHEVCQPPVVHQNFKSSKVLLDGKLSVRVADSGLAYML 650
AT1G11130.1_Acc_ICE134         NIALGASKALQFLHEVCQPPVVHQNFKSSKVLLDGKLSVRVADSGLAYML 650
AT1G11130.1_Acc_ICE130         NIALGASKALQFLHEVCQPPVVHQNFKSSKVLLDGKLSVRVADSGLAYML 650
AT1G11130.1_Acc_ICE119         NIALGASKALQFLHEVCQPPVVHQNFKSSKVLLDGKLSVRVADSGLAYML 650
AT1G11130.1_Acc_ICE111         NIALGASKALQFLHEVCQPPVVHQNFKSSKVLLDGKLSVRVADSGLAYML 650
AT1G11130.1_Acc_ICE106         NIALGASKALQFLHEVCQPPVVHQNFKSSKVLLDGKLSVRVADSGLAYML 650
```

```
AT1G11130.1_Acc_ICE104        NIALGASKALQFLHEVCQPPVVHQNFKSSKVLLDGKLSVRVADSGLAYML 650
AT1G11130.1_Acc_ICE1          NIALGASKALQFLHEVCQPPVVHQNFKSSKVLLDGKLSVRVADSGLAYML 650
AT1G11130.1_Acc_Fei-0         NIALGASKALQFLHEVCQPPVVHQNFKSSKVLLDGKLSVRVADSGLAYML 650
AT1G11130.1_Acc_Dog-4         NIALGASKALQFLHEVCQPPVVHQNFKSSKVLLDGKLSVRVADSGLAYML 650
AT1G11130.1_Acc_Cdm-0         NIALGASKALQFLHEVCQPPVVHQNFKSSKVLLDGKLSVRVADSGLAYML 650
AT1G11130.1_Acc_Bak-2         NIALGASKALQFLHEVCQPPVVHQNFKSSKVLLDGKLSVRVADSGLAYML 650
AT1G11130.1_Acc_Agu-1         NIALGASKALQFLHEVCQPPVVHQNFKSSKVLLDGKLSVRVADSGLAYML 650
AT1G11130.1_REF               NIALGASKALQFLHEVCQPPVVHQNFKSSKVLLDGKLSVRVADSGLAYML 650
AT1G11130.1_Acc_ICE120        NIALGASKALQFLHEVCQPPVVHQNFKSSKVLLDGKLSVRVADSGLAYML 650
AT1G11130.1_Acc_Tuescha-9     NIALGASKALQFLHEVCQPPVVHQNFKSSKVLLDGKLSVRVADSGLAYML 650
AT1G11130.1_Acc_TueWa1-2      NIALGASKALQFLHEVCQPPVVHQNFKSSKVLLDGKLSVRVADSGLAYML 650
AT1G11130.1_Acc_Vash-1        NIALGASKALQFLHEVCQPPVVHQNFKSSKVLLDGKLSVRVADSGLAYML 650
AT1G11130.1_Acc_Pra-6         NIALGASKALQFLHEVCQPPVVHQNFKSSKVLLDGKLSVRVADSGLAYML 650
AT1G11130.1_Acc_ICE102        NIALGASKALQFLHEVCQPPVVHQNFKSSKVLLDGKLSVRVADSGLAYML 650
AT1G11130.1_Acc_Qui-0         NIALGASKALQFLHEVCQPPVVHQNFKSSKVLLDGKLSVRVADSGLAYML 650
AT1G11130.1_Acc_ICE61         NIALGASKALQFLHEVCQPPVVHQNFKSSKVLLDGKLSVRVADSGLAYML 650
                              **************************************************

AT1G11130.1_Acc_Del-10        PPRPTSQMAGYAAPEVEYGSYTCQSDVFSLGVVMLELLTGRRPFDRTRPR 700
AT1G11130.1_Acc_ICE107        PPRPTSQMAGYAAPEVEYGSYTCQSDVFSLGVVMLELLTGRRPFDRTRPR 700
AT1G11130.1_Acc_Nie1-2        PPRPTSQMAGYAAPEVEYGSYTCQSDVFSLGVVMLELLTGRRPFDRTRPR 700
AT1G11130.1_Acc_Ped-0         PPRPTSQMAGYAAPEVEYGSYTCQSDVFSLGVVMLELLTGRRPFDRTRPR 700
AT1G11130.1_Acc_Koch-1        PPRPTSQMAGYAAPEVEYGSYTCQSDVFSLGVVMLELLTGRRPFDRTRPR 700
AT1G11130.1_Acc_TueSB30-3     PPRPTSQMAGYAAPEVEYGSYTCQSDVFSLGVVMLELLTGRRPFDRTRPR 700
AT1G11130.1_Acc_Yeg-1         PPRPTSQMAGYAAPEVEYGSYTCQSDVFSLGVVMLELLTGRRPFDRTRPR 700
AT1G11130.1_Acc_ICE216        PPRPTSQMAGYAAPEVEYGSYTCQSDVFSLGVVMLELLTGRRPFDRTRPR 700
AT1G11130.1_Acc_ICE72         PPRPTSQMAGYAAPEVDYGSYTCQSDVFSLGVVMLELLTGRRPFDRTRPR 700
AT1G11130.1_Acc_ICE50         PPRPTSQMAGYAAPEVEYGSYTCQSDVFSLGVVMLELLTGRRPFDRTRPR 700
AT1G11130.1_Acc_ICE60         PPRPTSQMAGYAAPEVEYGSYTCQSDVFSLGVVMLELLTGRRPFDRTRPR 700
AT1G11130.1_Acc_Mer-6         PPRPTSQMAGYAAPEVEYGSYTCQSDVFSLGVVMLELLTGRRPFDRTRPR 700
AT1G11130.1_Acc_Nemrut-1      PPRPTSQMAGYAAPEVEYGSYTCQSDVFSLGVVMLELLTGRRPFDRTRPR 700
AT1G11130.1_Acc_Sha           PPRPTSQMAGYAAPEVEYGSYTCQSDVFSLGVVMLELLTGRRPFDRTRPR 700
AT1G11130.1_Acc_Rue3-1-31     PPRPTSQMAGYAAPEVEYGSYTCQSDVFSLGVVMLELLTGRRPFDRTRPR 700
AT1G11130.1_Acc_Leo-1         PPRPTSQMAGYAAPEVEYGSYTCQSDVFSLGVVMLELLTGRRPFDRTRPR 700
AT1G11130.1_Acc_Istisu-1      PPRPTSQMAGYAAPEVEYGSYTCQSDVFSLGVVMLELLTGRRPFDRTRPR 700
AT1G11130.1_Acc_ICE98         PPRPTSQMAGYAAPEVEYGSYTCQSDVFSLGVVMLELLTGRRPFDRTRPR 700
AT1G11130.1_Acc_ICE97         PPRPTSQMAGYAAPEVEYGSYTCQSDVFSLGVVMLELLTGRRPFDRTRPR 700
AT1G11130.1_Acc_ICE92         PPRPTSQMAGYAAPEVEYGSYTCQSDVFSLGVVMLELLTGRRPFDRTRPR 700
AT1G11130.1_Acc_ICE79         PPRPTSQMAGYAAPEVEYGSYTCQSDVFSLGVVMLELLTGRRPFDRTRPR 700
AT1G11130.1_Acc_ICE75         PPRPTSQMAGYAAPEVEYGSYTCQSDVFSLGVVMLELLTGRRPFDRTRPR 700
AT1G11130.1_Acc_ICE71         PPRPTSQMAGYAAPEVEYGSYTCQSDVFSLGVVMLELLTGRRPFDRTRPR 700
AT1G11130.1_Acc_ICE7          PPRPTSQMAGYAAPEVEYGSYTCQSDVFSLGVVMLELLTGRRPFDRTRPR 700
AT1G11130.1_Acc_ICE49         PPRPTSQMAGYAAPEVEYGSYTCQSDVFSLGVVMLELLTGRRPFDRTRPR 700
AT1G11130.1_Acc_ICE36         PPRPTSQMAGYAAPEVEYGSYTCQSDVFSLGVVMLELLTGRRPFDRTRPR 700
AT1G11130.1_Acc_ICE33         PPRPTSQMAGYAAPEVEYGSYTCQSDVFSLGVVMLELLTGRRPFDRTRPR 700
AT1G11130.1_Acc_ICE228        PPRPTSQMAGYAAPEVEYGSYTCQSDVFSLGVVMLELLTGRRPFDRTRPR 700
AT1G11130.1_Acc_ICE213        PPRPTSQMAGYAAPEVEYGSYTCQSDVFSLGVVMLELLTGRRPFDRTRPR 700
AT1G11130.1_Acc_ICE212        PPRPTSQMAGYAAPEVEYGSYTCQSDVFSLGVVMLELLTGRRPFDRTRPR 700
AT1G11130.1_Acc_ICE21         PPRPTSQMAGYAAPEVEYGSYTCQSDVFSLGVVMLELLTGRRPFDRTRPR 700
AT1G11130.1_Acc_ICE173        PPRPTSQMAGYAAPEVEYGSYTCQSDVFSLGVVMLELLTGRRPFDRTRPR 700
AT1G11130.1_Acc_ICE169        PPRPTSQMAGYAAPEVEYGSYTCQSDVFSLGVVMLELLTGRRPFDRTRPR 700
AT1G11130.1_Acc_ICE153        PPRPTSQMAGYAAPEVEYGSYTCQSDVFSLGVVMLELLTGRRPFDRTRPR 700
AT1G11130.1_Acc_ICE150        PPRPTSQMAGYAAPEVEYGSYTCQSDVFSLGVVMLELLTGRRPFDRTRPR 700
AT1G11130.1_Acc_ICE138        PPRPTSQMAGYAAPEVEYGSYTCQSDVFSLGVVMLELLTGRRPFDRTRPR 700
AT1G11130.1_Acc_ICE134        PPRPTSQMAGYAAPEVEYGSYTCQSDVFSLGVVMLELLTGRRPFDRTRPR 700
AT1G11130.1_Acc_ICE130        PPRPTSQMAGYAAPEVEYGSYTCQSDVFSLGVVMLELLTGRRPFDRTRPR 700
AT1G11130.1_Acc_ICE119        PPRPTSQMAGYAAPEVEYGSYTCQSDVFSLGVVMLELLTGRRPFDRTRPR 700
AT1G11130.1_Acc_ICE111        PPRPTSQMAGYAAPEVEYGSYTCQSDVFSLGVVMLELLTGRRPFDRTRPR 700
AT1G11130.1_Acc_ICE106        PPRPTSQMAGYAAPEVEYGSYTCQSDVFSLGVVMLELLTGRRPFDRTRPR 700
AT1G11130.1_Acc_ICE104        PPRPTSQMAGYAAPEVEYGSYTCQSDVFSLGVVMLELLTGRRPFDRTRPR 700
AT1G11130.1_Acc_ICE1          PPRPTSQMAGYAAPEVEYGSYTCQSDVFSLGVVMLELLTGRRPFDRTRPR 700
AT1G11130.1_Acc_Fei-0         PPRPTSQMAGYAAPEVEYGSYTCQSDVFSLGVVMLELLTGRRPFDRTRPR 700
AT1G11130.1_Acc_Dog-4         PPRPTSQMAGYAAPEVEYGSYTCQSDVFSLGVVMLELLTGRRPFDRTRPR 700
AT1G11130.1_Acc_Cdm-0         PPRPTSQMAGYAAPEVEYGSYTCQSDVFSLGVVMLELLTGRRPFDRTRPR 700
AT1G11130.1_Acc_Bak-2         PPRPTSQMAGYAAPEVEYGSYTCQSDVFSLGVVMLELLTGRRPFDRTRPR 700
AT1G11130.1_Acc_Agu-1         PPRPTSQMAGYAAPEVEYGSYTCQSDVFSLGVVMLELLTGRRPFDRTRPR 700
AT1G11130.1_REF               PPRPTSQMAGYAAPEVEYGSYTCQSDVFSLGVVMLELLTGRRPFDRTRPR 700
AT1G11130.1_Acc_ICE120        PPRPTSQMAGYAAPEVEYGSYTCQSDVFSLGVVMLELLTGRRPFDRTRPR 700
AT1G11130.1_Acc_Tuescha-9     PPRPTSQMAGYAAPEVEYGSYTCQSDVFSLGVVMLELLTGRRPFDRTRPR 700
AT1G11130.1_Acc_TueWa1-2      PPRPTSQMAGYAAPEVEYGSYTCQSDVFSLGVVMLELLTGRRPFDRTRPR 700
AT1G11130.1_Acc_Vash-1        PPRPTSQMAGYAAPEVEYGSYTCQSDVFSLGVVMLELLTGRRPFDRTRPR 700
AT1G11130.1_Acc_Pra-6         PPRPTSQMAGYAAPEVEYGSYTCQSDVFSLGVVMLELLTGRRPFDRTRPR 700
AT1G11130.1_Acc_ICE102        PPRPTSQMAGYAAPEVEYGSYTCQSDVFSLGVVMLELLTGRRPFDRTRPR 700
AT1G11130.1_Acc_Qui-0         PPRPTSQMAGYAAPEVEYGSYTCQSDVFSLGVVMLELLTGRRPFDRTRPR 700
AT1G11130.1_Acc_ICE61         PPRPTSQMAGYAAPEVEYGSYTCQSDVFSLGVVMLELLTGRRPFDRTRPR 700
                              ****************:*********************************

AT1G11130.1_Acc_Del-10        GHQTLAQWAIPRLHDIDALTRMVDPSLHGAYPMKSLSRFADIISRSLQME 750
AT1G11130.1_Acc_ICE107        GHQTLAQWAIPRLHDIDALTRMVDPSLHGAYPMKSLSRFADIISRSLQME 750
AT1G11130.1_Acc_Nie1-2        GHQTLAQWAIPRLHDIDALTRMVDPSLHGAYPMKSLSRFADIISRSLQME 750
AT1G11130.1_Acc_Ped-0         GHQTLAQWAIPRLHDIDALTRMVDPSLHGAYPMKSLSRFADIISRSLQME 750
AT1G11130.1_Acc_Koch-1        GHQTLAQWAIPRLHDIDALTRMVDPSLHGAYPMKSLSRFADIISRSLQME 750
AT1G11130.1_Acc_TueSB30-3     GHQTLAQWAIPRLHDIDALTRMVDPSLHGAYPMKSLSRFADIISRSLQME 750
AT1G11130.1_Acc_Yeg-1         GHQTLAQWAIPRLHDIDALTRMVDPSLHGAYPMKSLSRFADIISRSLQME 750
AT1G11130.1_Acc_ICE216        GHQTLAQWAIPRLHDIDALTRMVDPSLHGAYPMKSLSRFADIISRSLQME 750
AT1G11130.1_Acc_ICE72         GHQTLAQWAIPRLHDIDALTRMVDPSLHGAYPMKSLSRFADIISRSLQME 750
AT1G11130.1_Acc_ICE50         GHQTLAQWAIPRLHDIDALTRMVDPSLHGAYPMKSLSRFADIISRSLQME 750
AT1G11130.1_Acc_ICE60         GHQTLAQWAIPRLHDIDALTRMVDPSLHGAYPMKSLSRFADIISRSLQME 750
AT1G11130.1_Acc_Mer-6         GHQTLAQWAIPRLHDIDALTRMVDPSLHGAYPMKSLSRFADIISRSLQME 750
AT1G11130.1_Acc_Nemrut-1      GHQTLAQWAIPRLHDIDALTRMVDPSLHGAYPMKSLSRFADIISRSLQME 750
AT1G11130.1_Acc_Sha           GHQTLAQWAIPRLHDIDALTRMVDPSLHGAYPMKSLSRFADIISRSLQME 750
AT1G11130.1_Acc_Rue3-1-31     GHQTLAQWAIPRLHDIDALTRMVDPSLHGAYPMKSLSRFADIISRSLQME 750
AT1G11130.1_Acc_Leo-1         GHQTLAQWAIPRLHDIDALTRMVDPSLHGAYPMKSLSRFADIISRSLQME 750
AT1G11130.1_Acc_Istisu-1      GHQTLAQWAIPRLHDIDALTRMVDPSLHGAYPMKSLSRFADIISRSLQME 750
```

```
AT1G11130.1_Acc_ICE98         GHQTLAQWAIPRLHDIDALTRMVDPSLHGAYPMKSLSRFADIISRSLQME 750
AT1G11130.1_Acc_ICE97         GHQTLAQWAIPRLHDIDALTRMVDPSLHGAYPMKSLSRFADIISRSLQME 750
AT1G11130.1_Acc_ICE92         GHQTLAQWAIPRLHDIDALTRMVDPSLHGAYPMKSLSRFADIISRSLQME 750
AT1G11130.1_Acc_ICE79         GHQTLAQWAIPRLHDIDALTRMVDPSLHGAYPMKSLSRFADIISRSLQME 750
AT1G11130.1_Acc_ICE75         GHQTLAQWAIPRLHDIDALTRMVDPSLHGAYPMKSLSRFADIISRSLQME 750
AT1G11130.1_Acc_ICE71         GHQTLAQWAIPRLHDIDALTRMVDPSLHGAYPMKSLSRFADIISRSLQME 750
AT1G11130.1_Acc_ICE7          GHQTLAQWAIPRLHDIDALTRMVDPSLHGAYPMKSLSRFADIISRSLQME 750
AT1G11130.1_Acc_ICE49         GHQTLAQWAIPRLHDIDALTRMVDPSLHGAYPMKSLSRFADIISRSLQME 750
AT1G11130.1_Acc_ICE36         GHQTLAQWAIPRLHDIDALTRMVDPSLHGAYPMKSLSRFADIISRSLQME 750
AT1G11130.1_Acc_ICE33         GHQTLAQWAIPRLHDIDALTRMVDPSLHGAYPMKSLSRFADIISRSLQME 750
AT1G11130.1_Acc_ICE228        GHQTLAQWAIPRLHDIDALTRMVDPSLHGAYPMKSLSRFADIISRSLQME 750
AT1G11130.1_Acc_ICE213        GHQTLAQWAIPRLHDIDALTRMVDPSLHGAYPMKSLSRFADIISRSLQME 750
AT1G11130.1_Acc_ICE212        GHQTLAQWAIPRLHDIDALTRMVDPSLHGAYPMKSLSRFADIISRSLQME 750
AT1G11130.1_Acc_ICE21         GHQTLAQWAIPRLHDIDALTRMVDPSLHGAYPMKSLSRFADIISRSLQME 750
AT1G11130.1_Acc_ICE173        GHQTLAQWAIPRLHDIDALTRMVDPSLHGAYPMKSLSRFADIISRSLQME 750
AT1G11130.1_Acc_ICE169        GHQTLAQWAIPRLHDIDALTRMVDPSLHGAYPMKSLSRFADIISRSLQME 750
AT1G11130.1_Acc_ICE153        GHQTLAQWAIPRLHDIDALTRMVDPSLHGAYPMKSLSRFADIISRSLQME 750
AT1G11130.1_Acc_ICE150        GHQTLAQWAIPRLHDIDALTRMVDPSLHGAYPMKSLSRFADIISRSLQME 750
AT1G11130.1_Acc_ICE138        GHQTLAQWAIPRLHDIDALTRMVDPSLHGAYPMKSLSRFADIISRSLQME 750
AT1G11130.1_Acc_ICE134        GHQTLAQWAIPRLHDIDALTRMVDPSLHGAYPMKSLSRFADIISRSLQME 750
AT1G11130.1_Acc_ICE130        GHQTLAQWAIPRLHDIDALTRMVDPSLHGAYPMKSLSRFADIISRSLQME 750
AT1G11130.1_Acc_ICE119        GHQTLAQWAIPRLHDIDALTRMVDPSLHGAYPMKSLSRFADIISRSLQME 750
AT1G11130.1_Acc_ICE111        GHQTLAQWAIPRLHDIDALTRMVDPSLHGAYPMKSLSRFADIISRSLQME 750
AT1G11130.1_Acc_ICE106        GHQTLAQWAIPRLHDIDALTRMVDPSLHGAYPMKSLSRFADIISRSLQME 750
AT1G11130.1_Acc_ICE104        GHQTLAQWAIPRLHDIDALTRMVDPSLHGAYPMKSLSRFADIISRSLQME 750
AT1G11130.1_Acc_ICE1          GHQTLAQWAIPRLHDIDALTRMVDPSLHGAYPMKSLSRFADIISRSLQME 750
AT1G11130.1_Acc_Fei-0         GHQTLAQWAIPRLHDIDALTRMVDPSLHGAYPMKSLSRFADIISRSLQME 750
AT1G11130.1_Acc_Dog-4         GHQTLAQWAIPRLHDIDALTRMVDPSLHGAYPMKSLSRFADIISRSLQME 750
AT1G11130.1_Acc_Cdm-0         GHQTLAQWAIPRLHDIDALTRMVDPSLHGAYPMKSLSRFADIISRSLQME 750
AT1G11130.1_Acc_Bak-2         GHQTLAQWAIPRLHDIDALTRMVDPSLHGAYPMKSLSRFADIISRSLQME 750
AT1G11130.1_Acc_Agu-1         GHQTLAQWAIPRLHDIDALTRMVDPSLHGAYPMKSLSRFADIISRSLQME 750
AT1G11130.1_REF               GHQTLAQWAIPRLHDIDALTRMVDPSLHGAYPMKSLSRFADIISRSLQME 750
AT1G11130.1_Acc_ICE120        GHQTLAQWAIPRLHDIDALTRMVDPSLHGAYPMKSLSRFADIISRSLQME 750
AT1G11130.1_Acc_Tuescha-9     GHQTLAQWAIPRLHDIDALTRMVDPSLHGAYPMKSLSRFADIISRSLQME 750
AT1G11130.1_Acc_TueWa1-2      GHQTLAQWAIPRLHDIDALTRMVDPSLHGAYPMKSLSRFADIISRSLQME 750
AT1G11130.1_Acc_Vash-1        GHQTLAQWAIPRLHDIDALTRMVDPSLHGAYPMKSLSRFADIISRSLQME 750
AT1G11130.1_Acc_Pra-6         GHQTLAQWAIPRLHDIDALTRMVDPSLHGAYPMKSLSRFADIISRSLQME 750
AT1G11130.1_Acc_ICE102        GHQTLAQWAIPRLHDIDALTRMVDPSLHGAYPMKSLSRFADIISRSLQME 750
AT1G11130.1_Acc_Qui-0         GHQTLAQWAIPRLHDIDALTRMVDPSLHGAYPMKSLSRFADIISRSLQME 750
AT1G11130.1_Acc_ICE61         GHQTLAQWAIPRLHDIDALTRMVDPSLHGAYPMKSLSRFADIISRSLQME 750
                              **************************************************

AT1G11130.1_Acc_Del-10        PGFRPPISEIVQDLQHMI 768
AT1G11130.1_Acc_ICE107        PGFRPPISEIVQDLQHMI 768
AT1G11130.1_Acc_Nie1-2        PGFRPPISEIVQDLQHMI 768
AT1G11130.1_Acc_Ped-0         PGFRPPISEIVQDLQHMI 768
AT1G11130.1_Acc_Koch-1        PGFRPPISEIVQDLQHMI 768
AT1G11130.1_Acc_TueSB30-3     PGFRPPISEIVQDLQHMI 768
AT1G11130.1_Acc_Yeg-1         PGFRPPISEIVQDLQHMI 768
AT1G11130.1_Acc_ICE216        PGFRPPISEIVQDLQHMI 768
AT1G11130.1_Acc_ICE72         PGFRPPISEIVQDLQHMI 768
AT1G11130.1_Acc_ICE50         PGFRPPISEIVQDLQHMI 768
AT1G11130.1_Acc_ICE60         PGFRPPISEIVQDLQHMI 768
AT1G11130.1_Acc_Mer-6         PGFRPPISEIVQDLQHMI 768
AT1G11130.1_Acc_Nemrut-1      PGFRPPISEIVQDLQHMI 768
AT1G11130.1_Acc_Sha           PGFRPPISEIVQDLQHMI 768
AT1G11130.1_Acc_Rue3-1-31     PGFRPPISEIVQDLQHMI 768
AT1G11130.1_Acc_Leo-1         PGFRPPISEIVQDLQHMI 768
AT1G11130.1_Acc_Istisu-1      PGFRPPISEIVQDLQHMI 768
AT1G11130.1_Acc_ICE98         PGFRPPISEIVQDLQHMI 768
AT1G11130.1_Acc_ICE97         PGFRPPISEIVQDLQHMI 768
AT1G11130.1_Acc_ICE92         PGFRPPISEIVQDLQHMI 768
AT1G11130.1_Acc_ICE79         PGFRPPISEIVQDLQHMI 768
AT1G11130.1_Acc_ICE75         PGFRPPISEIVQDLQHMI 768
AT1G11130.1_Acc_ICE71         PGFRPPISEIVQDLQHMI 768
AT1G11130.1_Acc_ICE7          PGFRPPISEIVQDLQHMI 768
AT1G11130.1_Acc_ICE49         PGFRPPISEIVQDLQHMI 768
AT1G11130.1_Acc_ICE36         PGFRPPISEIVQDLQHMI 768
AT1G11130.1_Acc_ICE33         PGFRPPISEIVQDLQHMI 768
AT1G11130.1_Acc_ICE228        PGFRPPISEIVQDLQHMI 768
AT1G11130.1_Acc_ICE213        PGFRPPISEIVQDLQHMI 768
AT1G11130.1_Acc_ICE212        PGFRPPISEIVQDLQHMI 768
AT1G11130.1_Acc_ICE21         PGFRPPISEIVQDLQHMI 768
AT1G11130.1_Acc_ICE173        PGFRPPISEIVQDLQHMI 768
AT1G11130.1_Acc_ICE169        PGFRPPISEIVQDLQHMI 768
AT1G11130.1_Acc_ICE153        PGFRPPISEIVQDLQHMI 768
AT1G11130.1_Acc_ICE150        PGFRPPISEIVQDLQHMI 768
AT1G11130.1_Acc_ICE138        PGFRPPISEIVQDLQHMI 768
AT1G11130.1_Acc_ICE134        PGFRPPISEIVQDLQHMI 768
AT1G11130.1_Acc_ICE130        PGFRPPISEIVQDLQHMI 768
AT1G11130.1_Acc_ICE119        PGFRPPISEIVQDLQHMI 768
AT1G11130.1_Acc_ICE111        PGFRPPISEIVQDLQHMI 768
AT1G11130.1_Acc_ICE106        PGFRPPISEIVQDLQHMI 768
AT1G11130.1_Acc_ICE104        PGFRPPISEIVQDLQHMI 768
AT1G11130.1_Acc_ICE1          PGFRPPISEIVQDLQHMI 768
AT1G11130.1_Acc_Fei-0         PGFRPPISEIVQDLQHMI 768
AT1G11130.1_Acc_Dog-4         PGFRPPISEIVQDLQHMI 768
AT1G11130.1_Acc_Cdm-0         PGFRPPISEIVQDLQHMI 768
AT1G11130.1_Acc_Bak-2         PGFRPPISEIVQDLQHMI 768
AT1G11130.1_Acc_Agu-1         PGFRPPISEIVQDLQHMI 768
AT1G11130.1_REF               PGFRPPISEIVQDLQHMI 768
AT1G11130.1_Acc_ICE120        PGFRPPISEIVQDLQHMI 768
AT1G11130.1_Acc_Tuescha-9     PGFRPPISEIVQDLQHMI 768
AT1G11130.1_Acc_TueWa1-2      PGFRPPISEIVQDLQHMI 768
```

```
AT1G11130.1_Acc_Vash-1         PGFRPPISEIVQDLQHMI 768
AT1G11130.1_Acc_Pra-6          PGFRPPISEIVQDLQHMI 768
AT1G11130.1_Acc_ICE102         PGFRPPISEIVQDLQHMI 768
AT1G11130.1_Acc_Qui-0          PGFRPPISEIVQDLQHMI 768
AT1G11130.1_Acc_ICE61          PGFRPPISEIVQDLQHMI 768
                               ******************
```
