# Supplementary material for: Structure-Function Analysis of STRUBBELIG, an Arabidopsis Atypical Receptor-Like Kinase Involved in Tissue Morphogenesis
Source: PLoS One. 2011 May 16;6(5):e19730. doi: 10.1371/journal.pone.0019730 (PMC3095605; doi:10.1371/journal.pone.0019730)
Supplement: Figure S4 — Quality assessment of the SUB kinase domain homology model. (PDF) [file pone.0019730.s004.pdf]

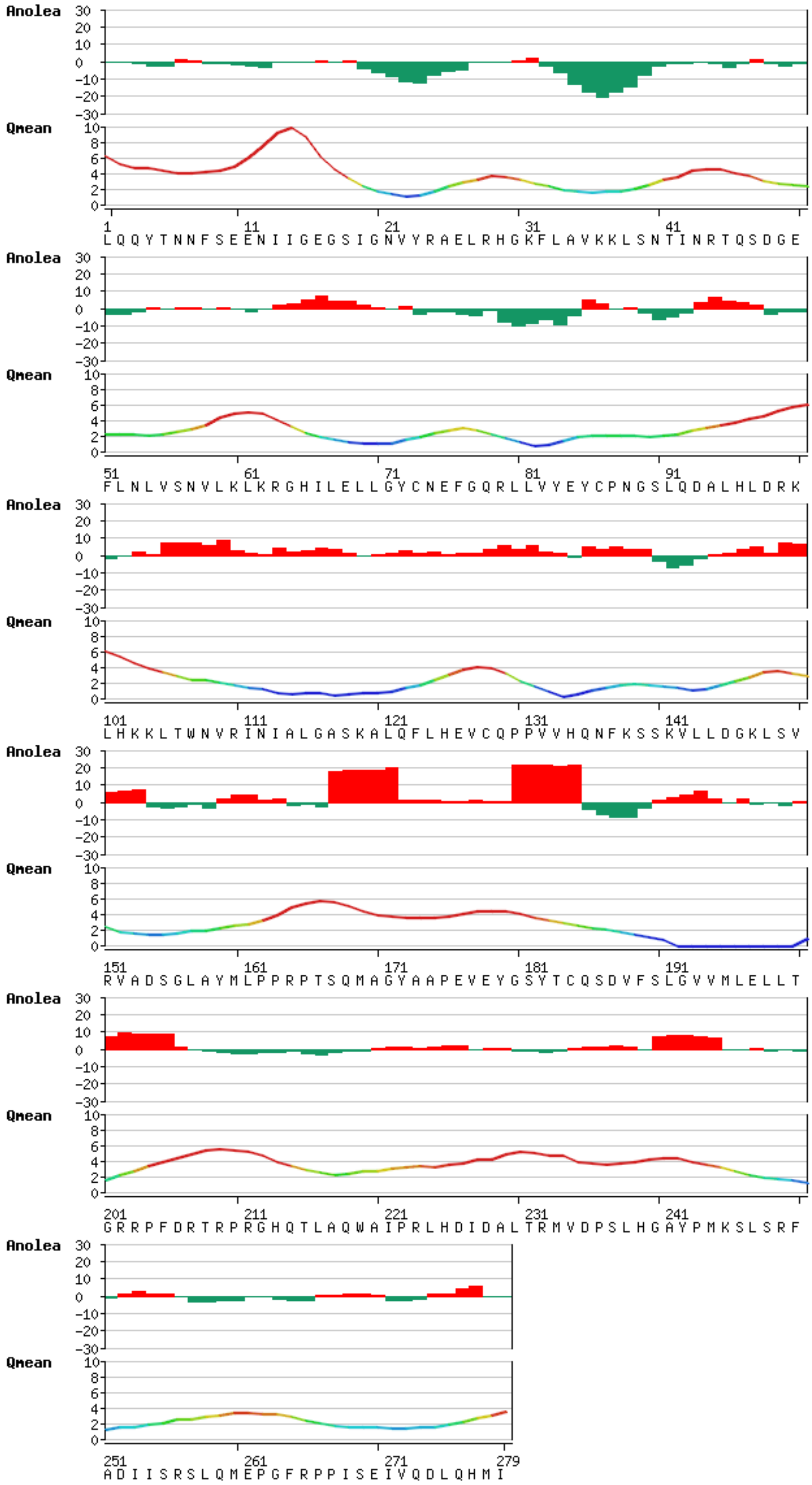

If you publish results using ANOLEA, please cite the following paper:
Melo, F. and Feytmans, E. (1998) Assessing Protein Structures with a Non-local Atomic Interaction Energy. Journal of Molecular Biology 277, 1141-1152

If you publish results from QMEAN, please cite the following paper:
Benkert, P., Schwede, T. and Tosatto, S.C.E. (2009). QMEANclust: Estimation of protein model quality by # combining a composite scoring function with structural density information. BMC Struct Biol. 2009 May 20;9:35.

Global Scores:

DFire: top

```
dfire_energy:   -332.64
```

If you publish results from DFire, please cite the following paper:
Zhou, H., and Zhou, Y. (2002). Distance-scaled, finite ideal-gas reference state improves structure-derived potentials of mean force for structure selection and stability prediction. Protein Sci. 11:2714-2726.

QMEAN: top

Global scores			Local scores	
QMEANscore6	Estimated absolute model quality	Score components	Coloring by residue error	Residue error plot
0.659	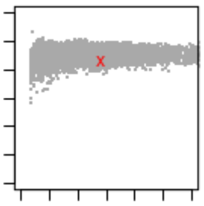 Z-Score: -1.207<br>Plot 1: [save png]<br>Plot 2: [save png]	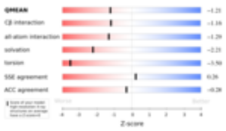 [save png]	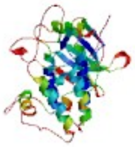	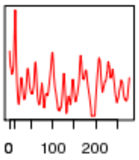 [save png]
			All residues error:<br>[save jpg] [save pdb]	Energy profile:<br>[save raw scores]

The QMEAN6 score is a composite score consisting of a linear combination of 6 terms (estimated model reliability between 0-1). The pseudo-energies of the contributing terms are given below together with their Z-scores with respect to scores obtained for high-resolution experimental structures of similar size solved by X-ray crystallography:		
Scoring function term	Raw score	Z-score
C_beta interaction energy	-74.39	-1.16
All-atom pairwise energy	-5563.18	-1.29
Solvation energy	-7.37	-2.21
Torsion angle energy	-14.64	-3.50
Secondary structure agreement	83.5%	0.26
Solvent accessibility agreement	78.9%	-0.28
QMEAN6 score	0.659	-1.21

If you publish results from QMEAN, please cite the following paper:
Benkert P, Biasini M, Schwede T. (2011). "Toward the estimation of the absolute quality of individual protein structure models." Bioinformatics, 27(3):343-50.

If you publish results using SWISS-MODEL, please cite the following papers:
Arnold K., Bordoli L., Kopp J., and Schwede T. (2006). The SWISS-MODEL Workspace: A web-based environment for protein structure homology modelling. Bioinformatics, 22,195-201.

* Disclaimer and user agreement: The use of SWISS-MODEL Server and SWISS-MODEL Workspace is free for both academic and commercial users. However, some of the tools provided on this page may require a valid license for some users. If you are in doubt, please check carefully the licensing conditions for each tools. Links to the original sites are provided in the help file. By using SWISS-MODEL workspace, you confirm that you are legally entitled to use these tools. You also agree to cite SWISS-MODEL Workspace AND the corresponding reference of these tools in any of your publications reporting results obtained from this service.

SWISS-MODEL is developed by the Protein Structure Bioinformatics group at the SIB - Swiss Institute of Bioinformatics & the Biozentrum University of Basel. © 2010.
