# Supplementary material for: Structure-Function Analysis of STRUBBELIG, an Arabidopsis Atypical Receptor-Like Kinase Involved in Tissue Morphogenesis
Source: PLoS One. 2011 May 16;6(5):e19730. doi: 10.1371/journal.pone.0019730 (PMC3095605; doi:10.1371/journal.pone.0019730)
Supplement: Table S1 — Primers used in this study. (DOC) [file pone.0019730.s005.doc]

**Table S1. Primers used in this study.**

| **Primer name** | **Sequence** |  |
| --- | --- | --- |
| SUB_cmyc_F | 5’-cctaggaggcgcgccatgagctttacaagatgggaagtgttc-3’ |  |
| SUB_cmyc_R | 5’-gagaccgacgtcagggccccgatcatatgttgaagatcttgg-3’ |  |
| SUB-Genomic2_F | 5’-GGCAAGTTTTCTCTGTTTCACACTTTGGAGACG-3’ |  |
| SUB-Genomic2_R | 5’-GCCCTCTGTGGAGCAGATTGATTTGAAGTTTA-3’ |  |
| Sig:SUB_Xba1_R | 5’-gctctagaccgacgtcagggc-3’ |  |
| SUBC57Yf | 5’-GCTTTTGGAGGAGACCCTTATGGAGAAAAGTGGCAAGGTG-3’ |  |
| SUBC57Yr | 5’-CACCTTGCCACTTTTCTCCATAAGGGTCTCCTCCAAAACG-3’ |  |
| 35SsubV64Mf | 5’-GAGAAAAGTGGCAAGGTATGGTGTGTGACTCCTCA-3’ |  |
| 35SsubV64Mr | 5’-TGAGGAGTCACACACCATACCTTGCCACTTTTCTC-3’ |  |
| SUB_C66Y_F | 5’-GGCAAGGTGTGGTGTATGACTCCTCAAACATC-3’ |  |
| SUB_C66Y_R | 5’-GATGTTTGAGGAGTCATACACCACACCTTGCC-3’ |  |
| SUB_C57-66Y_F | 5’-GGAGGAGACCCTTATGGAGAAAAGTGGCAAGGTGTGGTGTATGACTCCTCAAAC-3’ |  |
| SUB_C57-66Y_R | 5’-GTTTGAGGAGTCATACACCACACCTTGCCACTTTTCTCCATAAGGGTCTCCTCC-3’ |  |
| SUBC365-6Af | 5’-GTGTGTTACACTTTGGAGATATTACAGAAGTAAAATATATAACCG-3’ |  |
| SUBC365-6Ar | 5’-CGGTTATATATTTTACTTCTGCAACATCTCCAAAGTGTAACAC-3’ |  |
| G357S_SP | 5’-GCCATAATTGTTCTTGTATCCAGCTTGTGTGTTACACTTTGGAG-3’ |  |
| G357S_ASP | 5’-CTCCAAAGTGTAACACACAAGCTGGATACAAGAACAATTATGGC-3’ |  |
| SUBT486Af | 5’-CCTCATCTTCTGCTACTGTTTTCGCCATTGCTTCACTTCAGC -3’ |  |
| SUBT486Ar | 5’-GCTGAAGTGAAGCAATGGCGAAAACAGTAGCAGAAGATGA GG-3’ |  |
| SUBT486Ef | 5’-CCTCATCTTCTGCTACTGTTTTCGAAATTGCTTCACTTCAGC -3’ |  |
| SUBT486Er | 5’-GCTGAAGTGAAGCAATTTCGAAAACAGTAGCAGAAGATGA GG-3’ |  |
| SUBK525Ef | 5’-GGAAAGTTTCTTGCGGTGGAGAAGCTGAGCAATACCATCAAC-3’ |  |
| SUBK525Er | 5’-GTTGATGGTATTGCTCAGCTTCTCCACCGCAAGAAACTTTCC-3’ |  |
| SUBE539A35Sf | 5’-AGAACACAGAGTGACGGCGCATTCCTCAATCTAGTCTCC-3’ |  |
| SUBE539A35Sr | 5’-GGAGACTAGATTGAGGAATGCGCCGTCACTCTGTGTTCTG-3’ |  |
| 35SsubR599Cf | 5’-AAGAAGCTCACTTGGAATGTATGTATAAATATTGCATTAGG AGCTTC-3’ |  |
| 35SsubR599Cr | 5’-GAAGCTCCTAATGCAATATTTATACATACATTCCAAGTGAG CTTCTT-3’ |  |
| SUBT494Af | 5’-GCTTCACTTCAGCAATACGCAAATAATTTCTCAGAAGAG-3’ |  |
| SUBT494Ar | 5’-CTCTTCTGAGAAATTATTTGCGTATTGCTGAAGTGAAGC-3’ |  |
| SUBT494Ef | 5’-GCTTCACTTCAGCAATACGAAAATAATTTCTCAGAAGAG-3’ |  |
| SUBT494Er | 5’-CTCTTCTGAGAAATTATTTTCGTATTGCTGAAGTGAAGC-3 |  |
| 35S-extra-myc-rev | 5’-CCG ACG TCA GGG CCC CGA ATT TTC CTG ATC CTG AAC CTG A-3’ |  |
| 35S-TMmyc-rev | 5’-CCG ACG TCA GGG CCC CTC GGT TAT ATA TTT TAC TTC TGC A-3’ |  |
| JuxtraAatII-R | 5’-AAG ACG TCA GGG CCC CAT TAT TTG TGT ATT GCT GA-3’ |  |
| Alalinksignal-rev | 5’-GGC GGC GGC GGC GGC AAC TCC AGC TGA GAA AGG CAT TG-3’ |  |
| Alalink-TM-intra-for | 5’-GCC GCC GCC GCC GCC GCC TCA GGT TCA GGA TCA GGA AAA T-3’ |  |
| AscIntra-F | 5’-TGG CGC GCC ATG AGA TGT TGC AGA A-3’ |  |
